# Effectiveness of mobile apps in the secondary prevention of Chronic Coronary Syndrome: a systematic review and usability analysis^*^

**DOI:** 10.1590/1518-8345.8117.4982

**Published:** 2026-11-13

**Authors:** Taís Lins Severo da Silva, Gabriela Aparecida de Andrade Silva, Ligia Cristina Câmara Cunha, Vinicius Batista Santos, Juliana de Lima Lopes

**Affiliations:** 1 Universidade Federal de São Paulo, Escola Paulista de Enfermagem, São Paulo, SP, Brazil.

**Keywords:** Mobile Applications, Health Services Accessibility, Self Care, Coronary Disease, Healthy Lifestyle, Secondary Prevention.

## Abstract

**Objective:**

to identify evidence regarding the effectiveness of using mobile apps as a strategy to maintain healthy behaviors in patients with Chronic Coronary Syndrome and to analyze the usability of the best rated apps in Google Play^®^, using the System Usability Scale.

**Method:**

a methodological study conducted in two stages. The first one consisted in a systematic review, following the JBI recommendations. The GRADE tool was used to analyze the quality of the evidence corresponding to the outcomes. In the second stage, the mobile apps available in the two main Brazilian online stores were identified and the usability of those best rated by users in Google Play^®^ was analyzed, after due approval from a Research Ethics Committee.

**Results:**

eleven (79%) of the 14 articles showed that mobile apps are effective in promoting healthy behaviors for secondary prevention. Depending on the outcome evaluated, the methodological quality and risk of bias corresponding to the studies were considered between moderate and low. In relation to usability, only one was classified as average; the others were assessed as good, excellent or the best imaginable.

**Conclusion:**

despite the scarcity of studies about the effectiveness of these apps, most of them showed positive results, reinforcing their potential as a secondary prevention strategy.

## Highlights

**(1)** Mobile apps proved to be efficient in CCS secondary prevention.

**(2)** Their simple usability favors greater acceptance among users.

**(3)** Apps with feedback and intuitive navigation reach better results.

**(4)** Lack of guidance about sexual activity and gaps in the apps analyzed.

**(5)** Most of the apps are evaluated as good, excellent or the best imaginable.

## Introduction

Chronic non-communicable diseases (CNCDs), including cardiovascular diseases (CVDs), neoplasms, Diabetes Mellitus (DM) and chronic respiratory diseases, constitute the main causes of global morbidity and mortality, exerting significant impacts on quality of life and imposing high costs on health services^1^. It is estimated that CNCDs are responsible for nearly 41 million deaths per year, which corresponds to approximately 70% of the global cases and to more than 700,000 deaths in Brazil alone^1^ ^-^ ^2^.

CVDs stand out among CNCDs as the main cause of hospitalizations and deaths, accounting for approximately 17.9 million deaths per year worldwide^3^ ^-^ ^4^. In the Brazilian context, CVDs were responsible for almost 1.3 million hospitalizations in the Unified Health System (*Sistema Único de Saúde*, SUS) and for nearly 107,000 deaths in 2023, demanding high public system resources^5^ ^-^ ^7^.

In order to face this problem, the World Health Organization (WHO) created the Global Action Plan for Non-Communicable Diseases (2013-2030), aiming to reduce premature deaths due to CNCDs by one-third before 2030, in addition to expanding access to prevention, treatment and rehabilitation services^8^. The following stand out among the specific targets: 30% increase in drug therapies and counseling for CVD secondary prevention, as well as 50% expansion in the coverage of counseling programs for cardiovascular and pulmonary diseases^8^. Such targets converge with the United Nations (UN) Sustainable Development Goals (SDGs), which prioritize health and well-being promotion through prevention and effective treatments^9^.

One of the diseases that exert the most effect on premature mortality due to CNCDs is Chronic Coronary Syndrome (CCS), defined as the steady manifestation of coronary artery disease^10^. CCS is associated with various risk factors, turning secondary prevention (grounded on blood pressure and lipid control) into therapeutic adherence and into adopting healthy habits, something essential to reduce the number of ischemic complications^3^ ^,^ ^10^. Currently, the development of digital technologies targeted at health education and self-care has shown to be promising for CNCD secondary prevention. Mobile apps stand out among them as efficient tools for health monitoring and to promote healthy behaviors^11^ ^-^ ^18^. However, most of these solutions are focused on conditions such as DM, obesity and asthma, where they already exert a positive impact on engagement and on behavioral change^11^ ^,^ ^14^ ^-^ ^18^.

In the scope of cardiovascular diseases, mHealth interventions (including app use) have predominantly been targeted at managing isolated risk factors or heart failure, paying less attention to CCS^16^ ^-^ ^18^. It is noted that people with CCS face additional challenges to engage in secondary prevention activities, such as the following ones: treatment complexity, fatigue, dyspnea and fear of new ischemic events, factors that can demotivate practicing physical activity and adhering to treatments. These individuals also frequently present anxiety and depression, which exert a negative interference on perceived self-efficacy and, consequently, on adherence to secondary prevention measures^3^ ^,^ ^16^. In addition, low social support and the belief that pharmacotherapy alone is enough are added to this, which impairs comprehensive self-care^3^ ^,^ ^16^.

Thus, it becomes necessary to know the apps available, as well as the studies analyzing them, identifying the best theoretical grounds for care self-management, such as the Theory of Planned Behavior or the Transtheoretical Model, in addition to the functionalities and usability related to improving adherence to secondary prevention measures^16^ ^-^ ^25^. Given this scenario, the objective of this study is to combine a systematic literature review (devoted to analyzing the diverse evidence on the effectiveness of apps in adopting and maintaining CCS secondary prevention measures) with the usability analysis corresponding to the best rated tools available in Google Play^®^. By simultaneously analyzing effectiveness and usability, it is sought to identify the elements that favor engagement and self-care, expanding the application potential of these technologies in the clinical practice, particularly in nurse-mediated health education.

## Method

### Study design

This study was conducted in two stages. The first one consisted in a systematic literature review comprised by randomized clinical trials, following the Preferred Reporting Items for Systematic Reviews and Meta-Analyses (PRISMA 2020 Statement) guidelines^26^.

The second stage involved identifying the mobile apps available in the two main Brazilian online stores, targeted at promoting and maintaining healthy habits in people with CCS. Subsequently, the apps best rated by the users in Google Play^®^ were submitted to a usability analysis in order to verify their suitability and ease of use for this population group.

### Data collection setting

The systematic literature review was conducted in the MEDLINE/PubMed, SciELO, CINAHL, LILACS and Embase databases with no restrictions regarding time, age group or clinical conditions specific to CCS. The search strategies combined controlled descriptors (DeCS/MeSH/Emtree) with free terms related to CCS, to mobile apps and to secondary prevention, using AND and OR Boolean operators to expand sensitivity and specificity of the searches. In addition to the databases, a search was made in the grey literature (including theses, dissertations and technical reports), as well as a manual search in the lists of references from the articles selected, with the objective of identifying other potentially relevant studies.

The mobile apps targeted at CCS secondary prevention were identified in the two main Brazilian online stores: Google Play^®^ and Apple Store^®^. The apps from Google Play^®^ selected were subsequently analyzed as for their usability at a tertiary-level public hospital located in the city of São Paulo.

### Period

The systematic literature review was initially conducted on February 5^th^, 2024, and updated on November 5^th^, 2025, encompassing the previously defined electronic databases. This search was complemented on November 6^th^, 2025, with a manual exploration of the references in the articles selected and with a grey literature analysis. Referring to the identification of apps and to the usability analysis, the subsequent stage was developed from February to August 2024.

### Population

One of the researchers was in charge of selecting the apps in the online stores; in turn, the inclusion or exclusion analysis was performed independently by another two researchers. When in disagreement, a third researcher evaluated the situation and made the final decision. Finally, usability of the apps selected was analyzed by patients with CCS.

### Selection criteria

The review followed this guiding question: “Which is the scientific evidence available on the impact exerted by mobile apps on maintaining healthy habits targeted at secondary prevention in patients with Chronic Coronary Syndrome (CCS)?”.

This question was structured based on the PICO strategy, as follows: P (Patients) - individuals with Chronic Coronary Syndrome; I (Intervention) - mobile app use; C (Comparison) - other health education strategies; and O (Outcome) - maintaining healthy behaviors aimed at secondary prevention of the disease.

Studies published in English, Spanish and Portuguese were included, assessing the effectiveness of mobile apps in promoting healthy behaviors targeted at CCS secondary prevention. The search strategies combined controlled descriptors (DeCS, MeSH and Emtree) with free terms, associated by means of Boolean operators (AND, OR, NOT) and as detailed in Figure 1.

**Figure 1:** Search strategies according to each database. São Paulo, SP, Brazil, 2024

Database	Search strategy
PubMed	Keywords: (((mobile application[Title/Abstract])) AND ((Coronary Disease[Title/Abstract]) OR Coronary Artery Disease[Title/Abstract] OR Chronic Coronary Syndrome[Title/Abstract])). MeSH subject descriptors: (((mobile application[MeSH Terms]) OR (mobile health[MeSH Terms])) AND ((coronary occlusion[MeSH Terms]) OR (Coronary Thrombosis[MeSH Terms]) OR (Coronary Disease[MeSH Terms]) OR (Coronary Artery Disease[MeSH Terms])))
SciELO	Keywords: (((Mobile Application) OR (Aplicativos Móveis) OR (Aplicaciones Móviles) OR (Mobile Health)) AND ((Coronary Disease) OR (Coronary Artery Disease) OR (Doença coronariana) OR (Doença Arterial Coronariana) OR (Enfermedad de la Arteria Coronaria) OR (Enfermedad Coronaria)))
CINAHL	Keywords: (((Mobile Application) OR (Aplicativos Móveis) OR (Aplicaciones Móviles) OR (Mobile Health)) AND ((Coronary Disease) OR (Coronary Artery Disease) OR (Doença coronariana) OR (Doença Arterial Coronariana) OR (Enfermedad de la Arteria Coronaria) OR (Enfermedad Coronaria)))
MEDLINE/ LILACS	(mh:”Mobile Applications” OR mh:”Telemedicine” OR tw:”Mobile Application” OR tw:”Mobile Applications” OR tw:”Aplicativo Móvel” OR tw:”Aplicativos Móveis” OR tw:”mHealth” OR tw:”mobile health” OR tw:”smartphone” OR tw:”mobile app”) AND (mh:”Coronary Disease” OR mh:”Coronary Artery Disease” OR tw:”Coronary Disease” OR tw:”Coronary Artery Disease” OR tw:”Doença Arterial Coronariana” OR tw:”Doença Coronariana”) AND (mh:”Secondary Prevention” OR mh:”Life Style” OR tw:”Secondary Prevention” OR tw:”Prevenção Secundária” OR tw:”Healthy Lifestyle” OR tw:”Estilo de Vida Saudável”)
Embase	(‘mobile application’:ab,ti OR ‘aplicativos móveis’:ab,ti) AND (‘coronary disease’:ab,ti OR ‘coronary artery disease’:ab,ti OR ‘chronic coronary syndrome’:ab,ti OR ‘doença coronariana’:ab,ti OR ‘doença arterial coronariana’:ab,ti OR ‘síndrome coronariana crônica’:ab,ti) AND (‘secondary prevention’:ab,ti OR ‘healthy lifestyle’:ab,ti OR ‘prevenção secundária’:ab,ti OR ‘estilo de vida saudável’:ab,ti) AND (‘clinical article’/de OR ‘clinical study’/de OR ‘clinical trial’/de OR ‘comparative effectiveness’/de OR ‘controlled study’/de OR ‘evidence based practice’/de OR ‘major clinical study’/de OR ‘multicenter study’/de OR ‘phase 1 clinical trial’/de OR ‘phase 2 clinical trial’/de OR ‘phase 3 clinical trial’/de OR ‘randomized controlled trial’/de OR ‘randomized controlled trial topic’/de)

The following inclusion criteria were adopted to select apps in the online stores: free of charge; available in Portuguese; and presenting functionalities to maintain healthy habits targeted at CCS secondary prevention. The apps considered were those that addressed at least one of the topics recommended by the Brazilian secondary prevention guidelines, namely: adherence to drug therapies; practice of physical activity; healthy eating; stress, anxiety and depression management; control of risk factors (blood pressure, blood glucose, cholesterol and obesity); smoking cessation; alcohol consumption reduction; vaccination against influenza; and guidelines on sexual activity^10^.

In turn, the following terms were used for the search: “*doença arterial coronariana*” (“coronary artery disease”), “*síndrome coronariana crônica*” (“chronic coronary syndrome”), “*infarto agudo do miocárdio*” (“acute myocardial infarction”), “*angina*” and “*prevenção de doenças cardiovasculares*” (“prevention of cardiovascular diseases”), all included in the “Medicine” category of the Google Play^®^ and Apple Store^®^ online stores.

The inclusion criteria for the patients that took part in the usability assessment were as follows: no cognitive and/or visual deficits recorded in medical charts; sufficient literacy level to read and understand the instructions; and hospitalization in intensive care units or Cardiology wards (clinical or surgical) at a public hospital located in São Paulo.

### Definition of the sample

The materials included in the research were the studies selected in the systematic literature review and the apps found in the online stores for which two researchers reached agreement regarding their eligibility.

The sample used for the usability analysis was defined for convenience and was comprised by patients hospitalized in the Coronary unit during the data collection period. This choice is justified by the need to approach patients at a critical transitional care moment, the pre-discharge period, a stage where secondary prevention guidelines are intensified and when it is most opportune to incorporate digital self-management tools.

### Data collection

The search in the databases was in charge of a nurse researcher, PhD and professor at a Brazilian public university, who exported all the records identified to the Rayyan^®^ platform and automatically and manually removed all duplicates.

Subsequently, two independent reviewers (a Nursing student attending the final year of the course and a PhD student nurse) screened the studies by reading their titles and abstracts, assessing their eligibility based on the previously defined criteria. The potentially relevant articles were forwarded to the full-reading stage, where the same reviewers decided whether to include them or not.

In case of disagreement in any phase of the selection process, a third reviewer (the same professor responsible for the initial screening phase) evaluated the situation and made the final decision, ensuring reliability of the process and reducing biases.

Following the previously prepared standardized instrument, one of the researchers extracted the data from the studies included. The results were synthesized from a qualitative point of view, ensuring traceability of all the information and methodological transparency.

In the second stage, one of the researchers searched for apps in the Google Play^®^ and Apple Store^®^ online stores. The apps found were evaluated independently by another two reviewers, who defined their inclusion or not according to the previously established criteria. When in disagreement, a third reviewer analyzed the apps.

The eligible apps were classified according to the recommendations set forth in the Brazilian CCS secondary prevention guidelines, contemplating the following domains: adherence to drug therapies; practice of physical activity; adequate eating; anxiety reduction; obesity and overweight control; smoking cessation; blood pressure control; blood glucose control; alcohol consumption reduction; vaccination against influenza; education on knowledge about heart disease; and finally, cholesterol control^10^.

Once the apps had been categorized, the two apps best rated by the users in each of these categories were selected for the usability analysis stage, simultaneously considering the mean number of stars and the number of raters. That strategy was adopted to reduce biases resulting from overestimated ratings due to low number of assessments. At the end of this screening process, 24 and 22 apps meeting the criteria defined were identified in Google Play^®^ and Apple Store^®^, respectively.

Therefore, the 24 apps from Google Play^®^ were forwarded to the subsequent study stage, devoted to the usability analysis. The decision to exclusively evaluate apps from this online store was grounded on the predominance of the Android operating system among Brazilian users, which confers greater representativeness to the sample and enhance clinical applicability of the results. The apps exclusive to Apple Store^®^ were mapped but excluded from this stage due to the lower reach of this operating system among the population served and to logistic limitations to access the electronic devices required for testing.

In addition, the usability analysis was performed by one of the researchers along with the patients diagnosed with CCS that comprised the study target population. All the participants were provided with standardized instructions referring to the research objectives and procedures, ensuring uniformity in application and minimizing possible biases resulting from interpretation differences. After reading and signing the Free and Informed Consent Form (FICF), the patients were invited to handle the apps in a tablet for as long as they deemed necessary.

Subsequently, the participants filled in the usability assessment instrument. Each participant assessed three different apps, and each app was analyzed by six patients, in line with the recommendations found in the literature, according to which assessments by five to eight users are enough to identify most usability problems, ensuring representativeness and practical viability in the hospital context^27^.

### Study variables

The variables analyzed in the systematic review were as follows: name of the author(s); publication year and country; study objective; sample size; educational strategy used in the Control Group; name of the app(s); outcomes analyzed; and main results. In turn, the variables considered for the apps identified in the online stores were the following: title of the app(s); relationship with the topics from the CCS secondary prevention guidelines; number of users; and mean rating assigned by the users. In the final study stage, the variable analyzed was usability of the apps from Google Play^®^ best rated by the users.

### Instruments used to collect the information

The instrument used to extract the data from the articles selected in the systematic review and from the apps chosen in the online stores was created by the researchers and included the previously described variables.

Usability of the apps selected was analyzed with the System Usability Scale (SUS). Created in 1986 by John Brooke, SUS allows evaluating products and services, presenting the advantage of fast application for users and researchers alike. This scale is divided into three pillars: 1) Effectiveness: the user manages to achieve the goals; 2) Efficiency: how much effort and resources are required to achieve the goals; and 3) Satisfaction: it assesses whether the experience was satisfactory or not. It is divided into 10 questions with positive and negative assessment options and uses a five-point Likert scale from 1 (I totally disagree) to 5 (I totally agree)^28^ ^-^ ^29^. The final score is calculated as follows: 1 point is subtracted from the score indicated by the user in the case of the odd-numbered assertions; in turn, the value corresponding to the user answer score is subtracted from 5 in the even-numbered assertions. For example: if a user chooses 4 in question 1 (odd-numbered assertion), the value for this question will be 3; in turn, if 4 is selected for question 2 (even-numbered assertion), the score to be considered for that answer will be 1. After obtaining the individual score for each question, the values are added up and the result is multiplied by 2.5. Usability is classified as follows: the worst imaginable (up to 20.5), poor (from 21 to 38.5), average (from 39 to 52.5), good (from 53 to 73.5), excellent (from 74 to 85.5) or the best imaginable (from 86 to 100)^28^ ^-^ ^29^. The translation and transcultural adaptation process for the SUS scale to Brazilian Portuguese was conducted in 2021 by a group of researchers from *Universidade Estadual de Campinas* (UNICAMP), verifying its adequate psychometric properties^28^ ^-^ ^29^.

### Data treatment and analysis

The data extracted from the articles selected were organized in a Microsoft Excel^®^ spreadsheet containing the previously described variables. The analysis was performed from a qualitative point of view with the objective of identifying patterns and trends about the effectiveness of using mobile apps in maintaining healthy behaviors targeted at CCS secondary prevention. The assessment regarding methodological quality and risk of bias of the studies included was performed using the Grading of Recommendations Assessment, Development and Evaluation (GRADE) system, which classifies confidence in the evidence based on criteria referring to consistency, precision, directionality and applicability of the results. Such evaluation was performed independently by two reviewers, and any and all disagreements were solved by consensus. Having applied the GRADE system allowed ranking confidence in the evidence of the outcomes evaluated as high, moderate, low or very low, considering criteria such as risk of bias, inconsistency, indirect evidence, imprecision and publication bias. This stage conferred more transparency, methodological rigor and reliability to the synthesis of the results^30^.

It is also worth noting that the studies were categorized according to the educational strategy used, the type of app and the outcomes evaluated. In addition, all the information referring to the apps was also organized in an Excel^®^ spreadsheet, performing descriptive and quantitative analyses to verify their popularity based on the number of users and, consequently, on the mean rating assigned. The apps were also analyzed from a qualitative point of view and classified according to the CCS secondary prevention topics they addressed.

For this usability analysis stage, the scores obtained by means of the SUS scale were organized in a Microsoft Excel^®^ spreadsheet and submitted to descriptive statistics, including calculation of mean values, standard deviations, frequencies and percentages of the usability classification categories. A correlation analysis was also performed in Excel with the objective of verifying the relationship between the SUS scores and the characteristics of the apps evaluated, namely: app category; mean rating assigned by the users in the online stores; and number of assessments recorded. A 5% significance level (p<0.05) was adopted in all analyses.

### Ethical aspects

The protocol of this review was registered on the PROSPERO (International Prospective Register of Systematic Reviews) platform on October 24^th^, 2024, under code CRD420251176142 and with the following title: “Effectiveness of Mobile Applications in the Secondary Prevention of Chronic Coronary Syndrome: A Systematic Review and Usability Analysis”. The retrospective analysis was performed due to the need to update the protocol, including detailing the search strategies and the inclusion criteria. It is noted that the review was updated after due registration to thus ensure including the most recent studies and consistency with the reviewed protocol. These adjustments were of an exclusively organizational and methodological nature and did not alter the main outcomes, implementation of the analysis or interpretation of the results. Consequently, late registration reflects the final version of the protocol adopted to conduct the review, ensuring transparency and methodological rigor.

The research project was submitted to and approved by the Committee of Ethics in Research (*Comitê de Ética em Pesquisa*, CEP) with Human Beings belonging to *Universidade Federal de São Paulo*, under Opinion No. 6,620,843. Data collection was only initiated after obtaining that approval.

The participants who accepted to take part in the study signed a FICF, in compliance with the current ethical guidelines.

## Results

### First stage - Systematic literature review

*Identification and selection of studies*

The search was conducted in the MEDLINE/PubMed, SciELO, CINAHL, LILACS and Embase databases and was complemented by record repositories and a manual search in the references of the studies included. Two reviewers independently screened the studies in the Rayyan^®^ platform by reading their titles, abstracts and full texts. The process followed to identify, select and include studies is presented in the PRISMA flowchart (Figure 2).

**Figure 2:**
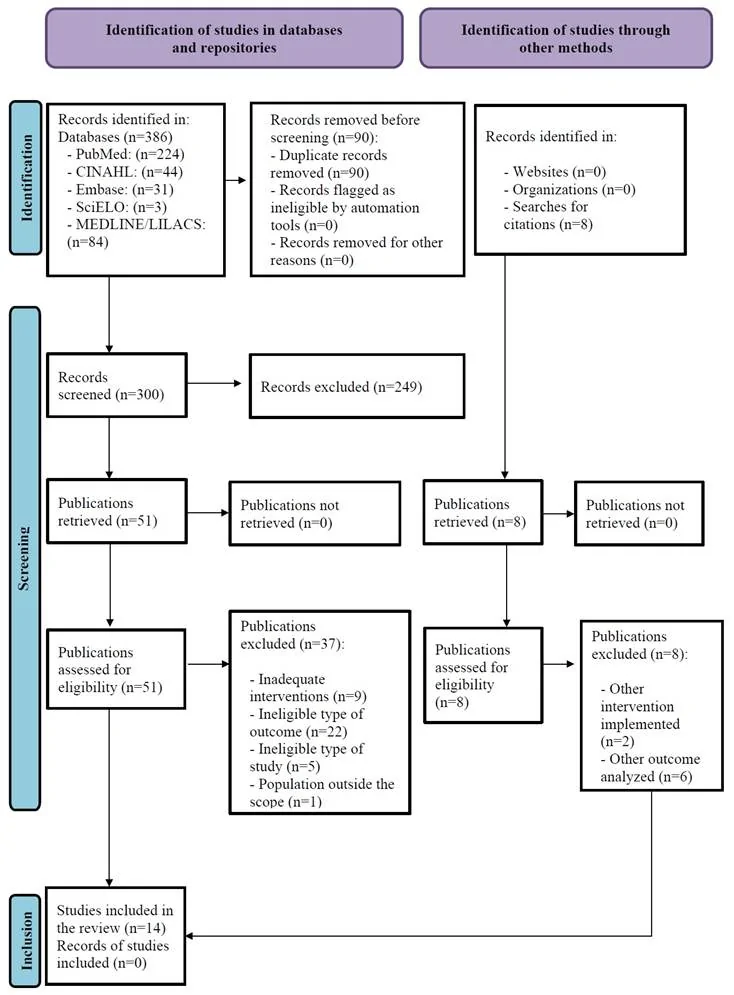
Flowchart showing the selection process for the articles included in the systematic literature review. São Paulo, SP, Brazil, 2025

*Characteristics of the studies included*

Figure 3 presents a synthesis corresponding to the main characteristics of the studies included in this systematic review, including publication year, objective, country, methodological design, sample size, interventions implemented, outcomes related to CCS secondary prevention and main results identified.

**Figure 3:** Synthesis of the main characteristics corresponding to the studies included in the systematic review. São Paulo, SP, Brazil, 2025

Author/Year	Objective	Country	Type of study	Sample	Interventions	Outcome(s)	Main result
Santo K, et al., 2019^31^	To assess the effectiveness of medication reminder apps to improve treatment adherence in patients with CAD*.	Australia	Three-armed randomized clinical trial.	156 patients with CAD* (56 in CG^†^, 54 in IG^‡^1, 53 in IG^‡^). *Mean age:* 57.9 years old *Gender:* 87.7% male *Comorbidity:* CAD*.	CG^†^: usual care with no app. IG^‡^1: app with basic reminders (daily reminders, with simple functions). IG^‡^2: app with advanced reminders (interactive and customizable resources).	Outcomes *- Adherence to drug therapies:* MMAS-8. *- Systolic BP* ^*§*^: digital record at rest, sitting down, mean of both measurements. *- Diastolic BP* ^*§*^: digital record at rest, sitting down, mean of both measurements. *- LDL-C* ^*∥*^: fasting blood sample. *- Total cholesterol:* fasting blood sample. Collection moments: At baseline and 3 months after randomization.	*- Adherence to drug therapies:* the mean MMAS-8 score was significantly higher (representing greater adherence to the medication) in the experimental groups when compared to the CG^†^ (mean difference: 0.47; 95%CI: 0.12; 0,82; p=0.008); *- Systolic BP* ^*§*^: no difference between the groups (mean difference: -0.89;95%CI: -6.97; 5.20; p=0.773); *- Diastolic BP* ^*§*^: no difference between the groups (mean difference: -0.87;95%CI: -6.97; 2.30; p=0.592); *- LDL-C* ^*∥*^: no difference between the groups (mean difference: 0.90; 95%CI: 0.73; 1.10; p=0.320); *- Total cholesterol:* no difference between the groups (mean difference: 0.00;95%CI: -0.31; 0.30; p=0.990).
Lunde P, et al., 2020^32^	To assess the effect exerted by individualized monitoring via a mobile app for one year after cardiac rehabilitation on physical exercise capacity and other outcomes in patients with heart disease.	Norway	Randomized and multicenter clinical trial, with blinded evaluators.	113 patients (57 in IG^‡^ and 56 in CG^†^) *Mean age:* not reported *Gender:* not reported *Comorbidity:* CAD*.	CG^†^: usual care. IG^‡^: smartphone app with 1-year individualized follow-up after cardiac rehabilitation.	Outcomes - *Practice of physical activity*: physical exercise performance, assessed as time elapsed until exhaustion, maximum tilt (%) and top speed (km/h), VO_2_^¶^ peak and effort perception as per the Borg scale in the cardiopulmonary test; physical exercise habits were assessed by evaluating the mean number of the exercise sessions per week in the last year. Each physical exercise session was defined as a structured activity lasting at least 30 minutes, which would make the person sweat and feel shortness of breath and leave the patient with the sensation that they would need to take a bath afterwards. - *Body weight:* measured barefoot and wearing sports clothes, before the cardiopulmonary test. *- BP* ^*§*^: measured at rest through manual methods, preferably on the left arm. *- Lipid profile*: blood sample collection. Collection moments: at baseline and after one year of cardiac rehabilitation.	*- Practice of physical activity:* a statistically significant difference was found in favor of the IG^‡^ in terms of VO_2_^¶^ peak between the groups after 1 year (mean difference: +2.2 ml/kg/minute; 95%CI: 0.9; 3.5; p<0.001); time elapsed until exhaustion (mean difference: 41 seconds; 95%CI: 9; 73; p=0.013) and maximum tilt (mean difference: 1.3%; 95%CI: 0.3; 2.4; p=0.014); physical exercise habit (mean difference +0.9 sessions/week; 95%CI: 0.4; 1,4; p<0.001). - There were no statistically significant differences between the groups in terms of body weight, BP^§^, lipid profile or other outcomes*.*
Song Y, et al., 2020^33^	To assess the application and effect of rehabilitation through physical exercise, telemonitored via smartphones among patients with CAD* in China.	China	Controlled, single-center and randomized clinical trial.	112 individuals with Coronary Artery Disease, aged less than 75 years old and with no physical or mental disorders (56 in IG^‡^ and 56 in CG^†^). *Mean age:* 54±9 years old *Gender:* 87% male *Comorbidities:* hypertension, dyslipidemia and diabetes *mellitus*.	CG^†^: routine treatment. IG^‡^: Telemonitoring app (MEMRS-CRS) and weekly contacts with the patients via text messages and telephone calls, providing feedback about performance in the physical exercises, solving doubts regarding how to use the devices and suggesting adjustments in the prescription of exercises.	Outcomes - *Practice of physical activity:* assessed by means of a questionnaire on periodicity, frequency and duration of each session and type of exercise, observing that the recommendations set forth in the 10^th^ Guidelines from the American College of Sports Medicine were met. *- BP* ^*§*^ values: measured with a sphygmomanometer (manual or automatic) at rest, before the physical exercise test. *- Blood glucose levels*: blood sample collection. *- Cholesterol levels*: blood sample collection. Collection moments: at baseline and after 6 follow-up months.	- *Practice of physical activity:* in favor of EG (93.8% versus 77.1%, p=0.021). *- BP* ^*§*^ values: no statistical data are presented, but it is described that there were no differences between the groups. *- Blood glucose levels*: no statistical data are presented, but it is described that there were no differences between the groups. *- Cholesterol levels*: no statistical data are presented, but it is described that there were no differences between the groups.
Yu C, et al., 2020^34^	To assess the effectiveness of a smartphone app in improving adherence to secondary prevention medications among patients after coronary revascularization.	China	Randomized, controlled and multi-center clinical trial.	1,000 participants subjected to myocardial revascularization (n=501 in IG^‡^, n=499 in CG^†^). *Mean age:* 57.28±9.09 years old *Gender:* 85.5% male*Comorbidity:* CAD*.	CG^†^: usual care with no app. IG^‡^: access to a smartphone app specifically developed for the study, with medication reminders and educational materials on cardiac health, risk factors and self-monitoring, during 6 months.	Outcomes *- Adherence to drug therapies:* MMAS-8 *- BP* ^*§*^: measured in-person. *- BMI* ^**^: calculated based on weight and height. *- Smoking status:* self-reported*.* Collection moments: At baseline and 6 months after randomization.	*- Adherence to drug therapies:* Higher proportion of participants with low adherence, but without differences between the groups (11.8% in IG^‡^ and 11.7% in CG^†^ (RR=1.005, 95%CI: 0.682; 1.480; p=1.000). - There were no significant differences in the secondary clinical outcomes (BP^§^, BMI^**^, smoking) between the groups).
Li Y, et al., 2022^35^	To Investigate the effect exerted by a Digital Therapy (DTx^††^) intervention based on a self-management mobile app on long-term use of secondary prevention medications in patients with coronary disease.	China	Randomized, open and single-center pilot clinical trial.	290 patients hospitalized with CAD* (143 in IG^‡^ and 147 in CG^†^) *Mean age:* not reported *Gender:* not reported*Comorbidity:* CAD*.	CG^†^: post-discharge conventional follow-up (usual hospitalization/outpatient care) with no app. IG^‡^: periodic monitoring associated with using a self-management mobile app (medication plan, lifestyle intervention plan, follow-up protocol, patient education materials and allowing self-monitoring at home).	Outcomes *- Adherence to drug therapies:* use percentage of all the medications recommended by the guideline for secondary prevention at 12 months; Adherence percentage corresponding to the secondary prevention medications at 6 months. *- BP* ^*§*^ levels: BP^§^ control rate. *- Cholesterol levels:* blood sample collection at 6 and 12 months. Collection moments: Baseline, 6 months, 12 months.	*- Adherence to drug therapies:* at 12 months, the IG presented a better use proportion of all the recommended medications, when compared to the CG (RR=1.34; 95%CI: 1.121;0.61; p=0.001) *- BP* ^*§*^ levels: the IG presented a higher proportion of participants that achieved BP^§^ control at 12 months (RR=1.45; 95%CI: 1.22; 1.72; p<0.001) *- Cholesterol levels:* the IG^‡^ presented a higher proportion of participants that achieved LDLC^∥^<1.8 mmol/L at 12 months (RR=1.40; 95%CI: 1.11; 1.75; p=0.004).
Ni Z, et al., 2022^36^	To assess the effectiveness of a mobile health (mHealth^‡‡^) intervention using two mobile apps to improve adherence to the medication and health outcomes (BP^§^, HR^§§^) in patients with CAD*.	China	Randomized, parallel, not blinded and two-armed clinical trial.	Recruited: 230 patients with Coronary Cardiac Disease, analyzing 196 of them (103 in IG^‡^ and 93 in CG^†^). *Mean age:* 61±11 years old *Gender:* 80.1% male*Comorbidity:* CAD*.	CG^†^: it was only provided with educational materials via an app (WeChat) without customized reminders. IG^‡^: two apps were used: WeChat to receive educational materials and *Message Express* to send customized medication reminders.	Outcomes *- Adherence to drug therapies:* Voils Extent Scale *- BP* ^*§*^ levels: measured in-person. Collection moments: At baseline, at 60 days (end of the intervention) and at 90 days (30 days after the end of the intervention).	*- Adherence to drug therapies:* the drug non-adherence score was significantly reduced in the IG^‡^, both at 60 days (p=0.04) and at 90 days (p<0.001). - *BP* ^*§*^: The difference in the variation rate for systolic BP^§^ (β=-0.08, p<0.001) and diastolic BP^§^ (β=-0.05, p=0.004) between both groups was statistically significant in favor of the IG^‡^. The difference as for the change in the proportion of participants with normal systolic BP between both groups was statistically significant (β=0.01, p=0.02); however, the difference between the groups was not statistically significant for the proportion of participants with normal diastolic BP^§^ (β=0.006, p=0.32).
Boszko M, et al., 2023^37^	To assess the effectiveness of a mobile health app to support patients after PCI^∥∥^, with a focus on reducing anxiety and stress and on promoting self-care and adherence to clinical monitoring.	Poland	Randomized and controlled clinical trial.	67 post-PCI patients^∥∥^ (34 in IG^‡^ and 33 in CG) *Mean age:* 61 years old *Gender:* 65 % male*Comorbidity:* CAD*, heart failure.	CG^†^: usual care, with in-person guidelines and a printed booklet. IG^‡^: use of the Cardio Patient mobile app, containing educational modules on coronary disease, instructions for after the PCI procedure^∥∥^, self-care and appointment reminders, symptom monitoring, health diary and motivational messages.	Outcomes *- Anxiety, depression and stress levels:* DASS-21 questionnaire. Collection moments: At baseline (before hospital discharge),30 and 90 days after the procedure.	*- Anxiety, depression and stress levels:* There were no significant differences between the groups at any of the moments evaluated.
Beckie TM, et al. 2024^38^	To assess the effect of an mHealth^‡‡^ intervention (HerBeat) in women with CAD*, comparing usual educational care in terms of physical exercise capacity and the outcomes reported by the patients.	United States of America	Randomized and pilot clinical trial.	47 women with CAD* (23 in the HerBeat group, 24 in the Educational Care group) *Mean age:* 61.2±9.1 years old *Gender:* 100% female*Comorbidity:* CAD*.	CG^†^: standardized cardiac rehabilitation manual. IG^‡^: HerBeat: smartphone, associated with “smartwatch” and “health coach” (behavioral change intervention).	Outcomes *- Anxiety:* self-reported. *- Eating confidence:* self-efficacy in eating healthy. *- BP* ^*§*^: measured in-person. *- Waist circumference:* measured in-person. *- Body weight:* measured in-person. *- BMI* ^**^: calculated based on the participants’ weight and height. *- Physical activity level:* physical exercise capacity assessed via the 6-minute Gait Test. Collection moments: At baseline and after 3 months of intervention.	- The IG presented a significant improvement in 6 MWT from baseline to 3 months (p=0.016; effect size: d=0.558). Although lacking statistical significance, the difference between the groups at 3 months was 38 meters. From baseline to 3 months, the IG^‡^ presented significant improvements in: - Anxiety level (p=0.021); - Eating confidence (p=0.028); - Diastolic BP^§^ (p=0.03) - Waist circumference (p=0.008).
Bernal-Jiménez MÁ, et al; 2024^39^	To assess the effect exerted by an interactive mobile app (EVITE) in patients with CAD* already subjected to PCI^∥∥^ on adherence to healthy lifestyles, therapeutic adherence and control of cardiovascular risk factors.	Spain	Randomized, controlled and parallel clinical trial, with blinded statistician and analyzers and open for the patients due to the nature of the intervention.	134 patients with CAD* subjected to PCI^∥∥^ with stent implantation (67 in IG^‡^ and 67 in CG). *Mean age:* 59.49±8.97 years old *Gender:* 71.9% male*Comorbidities:* CAD*, hypertension, diabetes mellitus.	CG^†^: usual care, counseling on the Mediterranean diet, physical activity, smoking cessation and adherence to drug therapies. IG: app use for at least 15 minutes/day, during 36 weeks. Functionalities: education in healthy lifestyles and therapeutic targets based on guidelines; self-control and monitoring of physical activity, eating habits, BP^§^, smoking, weight, blood glucose and adherence to drug therapies; motivation via automatic reminders; customized messages; progress charts; and colors indicating target fulfillment.	Outcomes *- Suitable diet:* adherence to the Mediterranean diet assessed by means of the *Mediterranean Diet Adherence Score*; food consumption frequency (self-reported). - *Practice of physical activity: Minnesota Physical Activity Survey*, minutes/week. - *Smoking*: self-reported; dependence level assessed by means of Fagerström’s nicotine addiction test. *- Knowledge level regarding risk factors and healthy lifestyles*: instrument created and validated by the authors. - *Adherence to drug therapies:* Morisky Green & Levine 4 items. Collection moments: At baseline and 36 weeks after the intervention. The questionnaires were applied via telephone calls due to the COVID-19 pandemic.	The outcomes were favorable in the IG. *- Suitable diet:* adherence to the Mediterranean diet (mean score of 11.83±1.74 versus 10.14±2.02, p<0.001); proportion of patients with good adherence to the diet (91% versus 75%; p=0.02). *- Practice of physical activity:* mean of 619±318 minutes/week versus 472±261 minutes/week; p=0.007). *- Smoking cessation:* 76% (IG) versus 42% (CG^†^); p=0.01; assessed by means of Fagerström’s test, addiction to nicotine was lower in the Experimental Group (0.93±2.29 versus 2.27±2.82; p=0.02). *- Knowledge regarding healthy lifestyles:* also higher in the IG^‡^ (118.7±2.65 versus 111.25± 9.05; p<0.001). *- Adherence to drug therapies:* similar improvement in both groups (84% in mHealth^‡‡^ versus 79% in CG; not significant).
Cruz-Cobo C, et al., 2024^40^	To compare the effectiveness of a mobile health (mHealth^‡‡^) intervention using the eMOTIVA app to habitual care, in relation to adherence to cardiac rehabilitation and considering lifestyle aspects, cardiovascular risk factors and satisfaction among patients with acute coronary syndrome.	Spain	Randomized and controlled clinical trial, with a parallel-groups design.	300 patients with CAD* subjected to PCI^∥∥^ with stent implantation after an acute coronary syndrome episode (150 in IG^‡^ and 150 in CG). *Mean age:* 62.5±8.7 years old *Gender:* 69.0% male*Comorbidity:* CAD*.	CG^†^ *:* usual care. IG^‡^: access to mHealth^‡‡^ (e-MOTIVA app). The app offers scientific information to instruct users in attaining the therapeutic targets recommended in clinical guidelines and in maintaining a healthy lifestyle.	Outcomes *- Practice of physical activity: International Physical Activity Questionnaire* (IPAQ) and 6-minute Gait Test; *- Maintaining healthy eating: Adherence to the Mediterranean diet* and a questionnaire on consumption frequency in the last week. *- Knowledge level regarding risk factors* (stress and anxiety, high BP^§^, blood glucose, cholesterol, obesity): scale created and validated by the authors, with 24 items. - *Smoking cessation:* self-report and Fagerström’s test. *- Body weight and height to calculate Body Mass Index (BMI)* ^**^: measured in-person. *- Waist circumference:* measured in-person. *- Systolic and diastolic BP* ^*§*^ and heart rate: measured in-person. *- Lipid values (total cholesterol, HDL-C* ^*¶¶*^ , LDL-C ^*∥*^ and triglycerides), HbA1c ^***^ and blood glucose: blood sample collection. Collection moments: At baseline and after 3 and 6 follow-up months.	The IG^‡^ performed significantly better than the CG^†^ in all the outcomes, with a statistically significant difference. *- Practice of physical activity:* physical activity (minutes/week) after 3 follow-up months (578.10 +326.14 versus 443,46+278.11, p<0.001) and after six months (614.51+332.26 versus 408.40 +274.49, p<0.001); physical activity (METs/week) after 3 follow-up months (1,991.74 +1,176.71 versus 1,490.48 +925.89, p<0.001) and after six months (2,112.66+1,196.67 versus 1,372.60+944.62, p<0.001); hours in a seated position per week after 3 follow-up months (8.57+1.89 versus 9.34+2.13, p=0.002) and after six months (8.38+1.88 versus 9.59+2.09, p<0.001); six-minute gait test after six months (473.49+102.28 versus 447.25+93.68, p=0.04). *- Maintaining healthy eating:* adherence to the Mediterranean diet after 3 follow-up months (11.63+1.70 versus 9.32+2.55, p<0.001) and after six months (11.92+1.70 versus 8.92+2.66, p<0.001); good diet adherence after 3 follow-up months (93.8% versus 67.6%, p<0.001) and after six months (94.4% versus 60.3%, p<0.001).
Cruz-Cobo C, et al., 2024^40^	To compare the effectiveness of a mobile health (mHealth^‡‡^) intervention using the eMOTIVA app to habitual care, in relation to adherence to cardiac rehabilitation and considering lifestyle aspects, cardiovascular risk factors and satisfaction among patients with acute coronary syndrome.	Spain	Randomized and controlled clinical trial, with a parallel-groups design.	300 patients with CAD* subjected to PCI^∥∥^ with stent implantation after an acute coronary syndrome episode (150 in IG^‡^ and 150 in CG). *Mean age:* 62.5±8.7 years old *Gender:* 69.0% male*Comorbidity:* CAD*.	CG^†^ *:* usual care. IG^‡^: access to mHealth^‡‡^ (e-MOTIVA app). The app offers scientific information to instruct users in attaining the therapeutic targets recommended in clinical guidelines and in maintaining a healthy lifestyle.	Outcomes *- Practice of physical activity: International Physical Activity Questionnaire* (IPAQ) and 6-minute Gait Test; *- Maintaining healthy eating: Adherence to the Mediterranean diet* and a questionnaire on consumption frequency in the last week. *- Knowledge level regarding risk factors* (stress and anxiety, high BP^§^, blood glucose, cholesterol, obesity): scale created and validated by the authors, with 24 items. - *Smoking cessation:* self-report and Fagerström’s test. *- Body weight and height to calculate Body Mass Index (BMI)* ^**^: measured in-person. *- Waist circumference:* measured in-person. *- Systolic and diastolic BP* ^*§*^ and heart rate: measured in-person. *- Lipid values (total cholesterol, HDL-C* ^*¶¶*^ , LDL-C ^*∥*^ and triglycerides), HbA1c ^***^ and blood glucose: blood sample collection. Collection moments: At baseline and after 3 and 6 follow-up months.	*- Knowledge level regarding risk factors:* after 3 follow-up months (116.14+4.23 versus 111.02+6.94, p<0.001) and after six months (117.85+3.83 versus 111.00+7.11, p<0.001). - *Smoking cessation:* After 3 follow-up months (62.3% versus 53.2%, p=0.35) and after six months (65.3% versus 46.8%, p=0.06); addiction to nicotine after 3 follow-up months (2.30+2.27 versus 4.14+2.96, p=0.03) and after six months (2.18+2.37 versus 3.64+3.02, p=0.10). *- BMI* ^**^: after 3 follow-up months (28.33±4.48 versus 28.24±4.40, p=0.86) and after six months (28.22±4.69 versus 28.33±4.74, p=0.69). - *Waist circumference:* after 3 follow-up months (102.56±11.53 versus 102.22±10.35, p<0.80) and after six months (101.59±11.87 versus 102.09±10.80, p=0.72). *- Systolic BP* ^*§*^: after 3 follow-up months (128.96±15.87 versus 133.27±14.85, p<0.001) and after six months (130.00±21.90 versus 135.78±16.73, p<0.001). *- Diastolic BP* ^*§*^: after 3 follow-up months (73.68±9.76 versus 76.05±11.42, p=0.06) and after six months (117.85+3.83 versus 111.00+7.11, p<0.001). *- HR* ^*§§*^: after 3 follow-up months (66.75±8.91 versus 71.93±9.86, p=0.001) and after six months (68.20±10.13 versus 70.43±10.80, p<0.07). *- Lipid profile (total cholesterol, HDL-C* ^*¶¶*^ , LDL-C ^*∥*^ and triglycerides): they presented significant reductions in both groups, but without significant differences between them at any moment*:* after 3 follow-up months. *- HbA1c* ^***^: after 3 follow-up months (6.61±1.40% us 6.19±0.84%0.25) and after six months (6.44±1.01% versus 6.69+1.02%; p=0.36). *- Blood glucose:* after 3 follow-up months (110.58±35.92 mg/dL versus 112.44±11.42 mg/L, p=0.48) and after six months (116.48±45.55 mg/dL versus 128.06+55.69 mg/dL, p=0.02).
Ghavami M, et al., 2024^17^	To assess the Green Heart mobile app, which includes modules for BP^§^, DLP^†††^ and smoking cessation management, as well as improved BP^§^ and lipid control in patients with CAD*.	Iran	Two-armed, randomized and parallel clinical trial, blinded for the outcome evaluators (single-blinded).	Total of eligible subjects: 1,590 patients with CAD*, including 1,114 hypertensive individuals and 1,488 participants with dyslipidemia. - For the hypertension analysis, the participants were 545 individuals from the CG^†^ and 546 and from the IG^‡^; in turn, 728 from the CG and 714 from the IG were included for dyslipidemia. *Mean age:* not reported *Gender:* not reported*Comorbidity:* CAD*.	CG^†^: traditional education on the same topics, in paper format. EG: use of an app with three main modules (BP^§^ control, DLP^†††^ control and smoking cessation) and provision of a place for self-monitoring, reminders, automatic feedback and education.	Outcomes *- BP* ^*§*^ control: BP^§^ successful control in hypertensive individuals; *- DLP* ^*†††*^ control: proportion of patients with dyslipidemia and LDLC^∥^<70 mg/dL. Collection moments: at baseline and 6 months after having analyzed the outcomes.	*- BP* ^*§*^ control: 88.6% of the IG^‡^ participants had their BP^§^ controlled, versus 78.5% in the CG^†^ (OR=2.13, 95%CI: 1.51; 3.03, p<0.001). *- DLP* ^*†††*^ control: 24.8% of the IG^‡^ participants had their BP^§^ controlled, versus 16.1% in the CG^†^ (OR=1.72, 95%CI: 1.32; 2.26, p<0.001).
Ghavami M, et al., 2024^41^	To assess the impact exerted by the smoking cessation module in the Green Heart mobile app on changes in the behavior of current smokers with CAD*.	Iran	Two-armed, randomized and parallel clinical trial, with blinded evaluators (single-blinded).	1,590 eligible individuals with CAD*, of which 668 were smokers. 332 from the CG^†^ and 102 from the control were analyzed. *Mean age:* not reported *Gender:* not reported*Comorbidity:* CAD*.	CG^†^: traditional education on the same topics (smoking, cardiovascular risk), in paper format and not using the app. EGE: use of the Green Heart app, which includes a specific module for smoking cessation, with automated nicotine addiction assessment, creation of a customized plan to quit smoking, educational contents, motivational messages and real-time feedback to support patient adherence.	Outcomes *- Smoking cessation:* uninterrupted abstinence for at least 4 weeks during follow-up; lower tobacco consumption (number of cigarettes) or change in smoking status. Collection moments: At baseline and 24 weeks after the beginning of the interventions.	In the intention-to-treat analysis, the IG^‡^ reached significantly higher smoking cessation rates and reductions in tobacco use when compared to the CG (OR=2.14, 95%CI: 1.16; 3.97; p=0.015).
Waranski M, et al., 2024^42^	To assess the effectiveness of the customized RehaPlus+ eHealth^‖^ program to promote periodic physical activity in patients with CAD* after rehabilitation Phase II.	Germany	Controlled trial with parallel groups.	169 patients with CAD* after stent implantation or revascularization, after Phase II rehabilitation (44 in the RehaPlus+ group; 61 in the “usual care” group). *Mean age:* not reported *Gender:* not reported*Comorbidity:* CAD*.	CG^†^: conventional outpatient care (24 strength and resistance sessions lasting 90 minutes each for 6 months). IG^‡^: RehaPlus+ eHealth program for 24 weeks, motivational messages twice a week + case manager support.	Outcomes *- Practice of physical activity (minutes/week) and ADLs* ^*‡‡‡*^: Bewegungs- und Sportaktivität questionnaire. Collection moments: at baseline and six months after follow-up.	- Physical activity (minutes/week): after 6 months, the RehaPlus+ group reached a mean of 182±208 minutes/week of physical activity versus 119±175 minutes/week in the Control Group (p=0.15). - ADLs^‡‡‡^ level: 442.79±469.1 minutes/week in the RehaPlus*+* group versus 308.27±411.52 in the CG (p=0.20).
Xu L, et al., 2024^43^	To investigate if a smartphone-based gamification intervention might increase participation in physical activities and other physical and psychological outcomes in patients with CAD*.	China	Three-armed, randomized and controlled clinical trial.	108 patients with CAD*, allocated to three groups: 34 participants in the CG^†^, 36 in the individual gamified IG^‡^ and 38 in the team IG^‡^ (gamification associated with social interaction). *Mean age:* not reported *Gender:* not reported*Comorbidity:* CAD*.	CG^†^: standard intervention (defining step targets via the app, without additional gamification) for 12 weeks. Individual IG: individual app use with gamification (step targets, rewards, feedback) for 12 weeks. Team IG^‡^: using the same gamification than in the individual IG, but associated with a team social component for 12 weeks.	Outcomes - *Physical activity level:* change in the number of daily steps walked; proportion of days in which the step target was achieved. *- BMI* ^**^ and waist circumference: measured in-person. Collection moments: At baseline and after 12 weeks of the interventions.	In the individual IG^‡^, the gamified intervention significantly increased the number of daily steps when compared to the CG^†^ (+988 steps; 95%CI: 259; 1,717; p<0.01); this effect was maintained during follow-up (+819 steps; 95%CI: 24; 1,613; p<0.01). In addition to that, statistically significant differences were observed in BMI^**^ and waist circumference between the CC^†^ and the individual group. In the team IG^‡^ (where gamification included social interaction), no significant increase was noticed in the physical activity level when compared to the CG^†^.

*CAD = Coronary Artery Disease; ^†^CG = Control Group; ^‡^IG = Intervention Group; ^§^BP = Blood Pressure; ^∥^LDL-C = Low-Density Lipoprotein Cholesterol; ^¶^VO_2_ = Maximum oxygen consumption; ^**^BMI = Body Mass Index; ^††^DTx = Digital Therapy; ^‡‡^mHealth = Mobile Health; ^§§^HR = Heart Rate; ^∥∥^PCI = Percutaneous Coronary Intervention; ^¶¶^HDL-C = High-Density Lipoprotein Cholesterol; ^***^HbA1c = Glycated Hemoglobin; ^†††^DLP = Dyslipidemia; ^‡‡‡^ADLs = Activities of daily living

The synthesis of all 14 randomized clinical trials included in this systematic review evidenced broad heterogeneity regarding digital interventions, outcomes evaluated and measuring methods, but with an overall trend of positive results in specific CCS secondary prevention components. Statistical effectiveness was observed in favor of mHealth in the control of clinical risk factors (54.5%), in adherence to drug therapies (60%) and, more significantly, in the promotion of physical activity (71.4%), in smoking cessation (100%) and in adherence to healthy diets (100%). In contrast, the mental health outcomes presented more modest and uncertain results (50%). It is important to note that, among the recommendations foreseen in the Brazilian guidelines, three topics were not addressed in any of the studies analyzed, namely: alcohol consumption reduction; vaccination against influenza; and guidelines related to sexual activity, which reveals relevant gaps in the development of apps targeted at the population with CCS.

The synthesis corresponding to the certainty of the evidence for each outcome analyzed (grounded on the GRADE system domains) is detailed in Figure 4.

**Figure 4:** Synthesis corresponding to quality of the evidence, by outcome and according to the GRADE methodology. São Paulo, SP, Brazil, 2025

Outcome	Risk of bias	Inconsistency	Indirect evidence	Imprecision	Publication bias	Global certainty
Control of risk factors^17^ ^,^ ^31^ ^-^ ^36^ ^,^ ^38^ ^-^ ^40^ ^,^ ^43^	🟡 Severe*	🟢 Not severe	🟢 Not severe	🟢 Not severe	🟢 Not suspected	🟡 Moderate
Adherence to drug therapies^31^ ^,^ ^34^ ^-^ ^36^ ^,^ ^39^	🟡 Severe*	🟢 Not severe	🟢 Not severe	🟢 Not severe	🟢 Not suspected	🟡 Moderate
Physical activity^32^ ^-^ ^33^ ^,^ ^38^ ^-^ ^40^ ^,^ ^42^ ^-^ ^43^	🟡 Severe*	🟡 Severe^†^	🟢 Not severe	🟡 Severe^‡^	🟡 Suspected^§^	🔴 Low
Smoking cessation^34^ ^,^ ^39^ ^-^ ^41^	🟡 Severe*	🟢 Not severe	🟡 Severe^||^	🟢 Not severe	🟢 Not suspected	🔴 Low
Healthy eating^39^ ^-^ ^40^	🟡 Severe*	🟢 Not severe	🟡 Severe^||^	🟡 Severe^‡^	🟢 Not suspected	🔴 Low
Anxiety/Depression/Stress^37^ ^-^ ^38^	🔴 Extremely severe^¶^	🟡 Severe^†^	🟡 Severe^||^	🟡 Severe^‡^	🟡 Suspected^§^	🔴 Low
Alcohol consumption reduction	-	-	-	-	-	-
Vaccination against influenza	-	-	-	-	-	-
Guidelines on sexual activity	-	-	-	-	-	-

*Downgraded due to the open (not blinded) nature of the mHealth intervention; ^†^Downgraded due to high statistical and clinical heterogeneity (different intervention and measuring methods); ^‡^Downgraded for having included pilot studies with small samples (n<50) and large confidence intervals; ^§^Suspected in behavioral outcomes due to absence of protocol records for studies with positive results in small samples; ^||^Downgraded for having used outcomes solely based on self-report with no biochemical validation or direct observation; ^¶^Downgraded two levels due to the combination of non-blinding in subjective outcomes and high attrition rates (>20%)

Assessed according to the GRADE methodology, certainty of the evidence varied from moderate to low across the outcomes analyzed, as synthesized in Figure 4. Controlling risk factors and adherence to drug therapies presented moderate certainty, only downgraded in the risk of bias domain due to the open (not blinded) nature inherent to mHealth interventions. On the other hand, the “physical activity”, “smoking cessation”, “healthy eating” and “mental health” (anxiety, depression and stress) outcomes were classified as with low certainty.

In turn, this downgrading was due to high heterogeneity (inconsistency), to using self-report measures without resorting to quantifiable data such as using a pedometer (indirect evidence) and to the imprecision resulting from pilot studies with small samples. In the mental health outcome, the risk of bias was considered as extremely severe due to the combination of non-blinding and high attrition rates (>20%). The analysis of each of the articles (by outcome) can be seen in the supplementary material (available from: https://doi.org/10.48331/SCIELODATA.CR4I8U).

### Second stage - Usability analysis for the apps selected

In this stage, a search for apps was initially made in Google Play^®^ and in Apple Store^®^, following the previously standardized search method. The best rated app was selected for each CCS secondary prevention category, simultaneously considering number of users’ ratings and mean number of stars, so as to reduce biases resulting from overestimated grading or with few assessments.

The apps identified addressed different CCS secondary prevention domains in a fragmented way, such as the following ones: adherence to drug therapies; practice of physical activity; adequate eating; blood pressure and blood glucose control; smoking cessation; alcohol consumption reduction; anxiety management, body weight control; and vaccination against influenza. In general, the apps related to physical activity, adherence to drug therapies and healthy eating presented more assessments and better mean scores in both platforms.

Therefore, widely used apps such as MyTherapy - *Lembrete de Remédios*, *Alarme de Medicamentos*, Strava and MyFitnessPal stood out, reflecting higher dissemination and acceptance among the users. On the other hand, domains such as Smoking cessation, Alcohol consumption control, Blood pressure control and Blood glucose control presented fewer apps and a lower volume of ratings, especially in Apple Store^®^. In the Vaccination against influenza domain, institutional apps such as *Meu SUS Digital* and *e-saúde SP* were mainly identified.

In addition, no specific apps targeted at sexual life or comprehensively addressing all the CCS secondary prevention domains were identified, evidencing that the digital solutions available are concentrated on isolated behaviors or risk factors.

After the selection stage, the apps available in Google Play^®^ best rated by the users in each category (My Therapy; *Treino em Casa & Exercícios*; *Alimente-se - Dieta e Nutrição*; *Rootd - Alívio da Ansiedade*; *Treino para Mulheres, Fitness*; *Parar de Fumar Assistente*; *Rastreador de Pressão Arterial*; mySugar *- Controle a Diabetes*; *Balcão de Sobriedade -* EasyQuit; *Meu SUS Digital*; *Dicas de Saúde e Alimentação*; and WeightWar *- Rastreador de Peso*) were analyzed regarding usability by 24 patients hospitalized with CCS medical diagnoses.

It is noted that half of the participants in this stage were male and that the majority (83%) was hospitalized due to acute myocardial infarctions. In addition to that, more than half (70%) were former smokers and presented Systemic Arterial Hypertension (SAH) (83%) and a family history of cardiovascular diseases (87%). In turn, alcoholics (7%), current smokers (23%) and DM patients (30%) were minorities.

The apps were randomly distributed among all 24 participants. Performed by means of the SUS scale, the usability analysis revealed important variations in the scores, as shown in Table 1.

**Table 1:** Minimum and maximum scores, mean, standard deviation and classification corresponding to usability of the apps. São Paulo, SP, Brazil, 2024

App	Minimum and maximum scores	Mean	Standard Deviation	SUS* classification
My Therapy	34 - 60	50.0	26.41	Average
*Treino em Casa & Exercícios*	87.5 - 55	69.58	14.78	Good
*Alimente-se - Dieta e nutrição*	90 - 47.5	53.33	18.82	Good
*Rootd - Alívio da Ansiedade*	90 - 15	54.58	25.02	Good
*Treino para Mulheres, Fitness*	100 - 37.5	77.50	27.48	Excellent
*Parar de Fumar Assistente*	85 - 52.5	72.08	12.39	Good
*Rastreador de Pressão arterial*	90 - 57.5	68.33	12.31	Good
mySugr *- Controle a diabetes*	87.5 - 42.5	69.17	18.55	Good
*Balcão de sobriedade -* EasyQui*t*	92.5 - 55	70.0	15.08	Good
*Meu SUS digital*	100 - 82.5	89.58	6.60	The best imaginable
*Dicas de Saúde e Alimentação*	100 - 82.5	90.0	10.12	The best imaginable
WeightWar *- Rastreador de peso*	100 - 35	79.17	23.96	Excellent

*SUS = System Usability Scale

The strengths of the apps’ usability were related to the desire to use a system frequently and to confidence in using such system. On the other hand, the main weakness identified was related to the complexity inherent to each system.

When performing the correlation analysis between the SUS scores and the characteristics of the apps (app category, mean rating assigned by the users in the online stores and number of assessments), it was noticed that there was no significant correlation between the users’ mean ratings and the SUS scores (r=0.12; p=0.58) and that the number of assessments did also not present any significant correlation (r=0.08; p=0.69). On the other hand, the app category exerted a partial influence on the usability perception: apps devoted to physical activity and eating presented higher mean scores (approximately 70-80) when compared to those targeted at blood glucose control or adherence to drug therapy (approximately 50-60). These findings indicate that the usability levels perceived by the patients are more dependent on each app’s functionalities and design than on popularity or overall rating in the online stores.

In addition to that, a subgroup analysis was performed to verify if the participants’ characteristics exerted any influence on their perception regarding usability. For such end, the mean SUS scores were compared between men and women and between participants with and without systemic arterial hypertension, diabetes *mellitus* and family history of cardiovascular diseases, as well as between patients hospitalized due to acute myocardial infarctions and those with other chronic coronary syndrome forms.

Finally, no significant differences between the groups were identified in the analysis, indicating that the participants’ demographic and clinical characteristics exerted no influence on perceived usability. These findings suggest that the apps evaluated perform consistently across different user profiles.

## Discussion

The current study was conducted in two complementary stages: a systematic review of randomized clinical trials (RCTs) and a usability assessment of apps available in the Google Play^®^ store, contemplating recommendations for Chronic Coronary Syndrome (CCS) secondary prevention. Performing an integrated analysis between the diverse evidence in the literature and the functionalities of mobile tools is fundamental to ensure effectiveness and safety of the digital health interventions in this population segment.

Despite the significant increase in the number of apps targeted at cardiovascular health, it is noticed that many of them lack solid scientific grounds, offering functionalities whose effectiveness has no clinical proof. Such fact was evidenced in the current study, where it was verified that, although various apps emphasize adopting healthy behaviors, few clinical trials published in the literature effectively validate the impact exerted by these tools on clinical outcomes.

In addition, the systematic review comprised by 14 RCTs showed that, despite the heterogeneity found as for functionalities, outcomes evaluated and duration of the interventions, there is a global trend in favor of using apps to change health behaviors and control cardiovascular risk factors. It is also noticed that digital interventions are consistently effective in self-management domains such as adherence to drug therapies and practicing physical activity. Some studies show that using customized reminders and self-care plans via apps not only improves scores in adherence scales but also results in a higher percentage of patients that attain blood pressure and lipid targets at 12 months^36^. This correlation suggests that app-based support can fill a critical gap during the post-discharge period, offering ongoing monitoring that the traditional care model (oftentimes episodic) just cannot sustain.

These results are corroborated by recent meta-analyses, such as the one that assessed app effectiveness in reducing unplanned hospital readmissions in patients after acute myocardial infarctions or percutaneous coronary interventions. The results showed that such interventions significantly reduced readmissions when compared to standard care (RR=0.45; 95%CI: 0.23; 0.89; p=0.0219), with more expressive benefits in prolonged follow-up studies^44^. Another meta-analysis (involving 34 clinical trials) evidenced that mobile apps can reduce the incidence of more severe cardiac adverse events (RR=0.68; p=0.03), hospital readmission rates (RR=0.56; p<0.001), total cholesterol (WMD=-0.19; p=0.03), triglycerides (WMD=-0.24; p<0.001) and abdominal circumference (WMD=-1.92; p=0.01), in addition to promoting improvements in anxiety and depression scores. Among some of the additional benefits we can mention increased left ventricular ejection fraction, higher maximum VO_2_, longer distance traveled in the six-minute gait test and better drug therapy adherence rates^45^.

Despite these positive impacts, the current review evidenced that the tool is not universally efficient for all outcomes. Whereas the behavioral variables presented significant improvements, anthropometric indicators such as Body Mass Index and waist circumference showed more resistance to isolated interventions via apps. Therefore, nurses and multiprofessional teams should use these devices as support elements that require human supervision in the clinical practice, integrating them to nutritional and physical in-person counseling. The association between strategies and gamification should also be considered to expand the results. A study that analyzed a 12-week intervention with gamification elements such as step targets, rewards and feedback showed that this approach was significantly superior in terms of increasing the number of daily steps when compared to the group that only used the conventional app. Therefore, this suggests that interactivity and positive reinforcement are essential to support the patients’ engagement^43^.

It is noted that CCS is a complex condition where patients face multiple prescriptions and the need to implement substantial changes in their lifestyle. That complexity contributes to low treatment adherence, rendering fundamental for digital tools to be created based on behavioral change theories. However, few studies report such theoretical grounds, and cognitive-social, target setting, self-efficacy and self-determination theories stand out among those that do so^46^. The literature points out that the most effective interventions are those built interactively, culturally adapted and capable of providing solid educational and motivational contents^47^ ^-^ ^50^.

Another relevant aspect that was identified both in the systematic review and in the analysis of the apps available is that none of them comprehensively addresses all the CCS secondary prevention domains. Tools centered on only one or two domains can fragment the care provided, not contemplating critical behaviors. Most of the apps and studies reviewed focus on quantifiable factors such as blood pressure and physical activity, as they are behaviors that are easy to monitor and measure. This effectiveness in measurable domains is corroborated by diverse evidence indicating improvements in physical exercise capacity and in drug therapy adherence^40^ ^,^ ^51^. On the other hand, domains such as Healthy eating, Stress management and Smoking cessation were explored less frequently and presented inconsistent results, which can be related to the complexity inherent to these behaviors, which oftentimes require in-person clinical support or individualized behavioral therapies^51^ ^-^ ^52^. Absence of these modules indicates that many apps still fail to fully incorporate the recommendations set forth in the most recent guidelines, which can impair self-management.

In addition, as for the usability feature analyzed in the study second stage, it was observed that most of the apps presented from good to excellent performance, with friendly designs and intuitive navigation. The technical problems and complexity inherent to each system were identified as abandonment factors, whereas automatic feedback was associated with better results^31^ ^-^ ^32^. In turn, the correlation analysis indicated that the users’ mean ratings in the online stores were not significantly correlated with the SUS scores, suggesting that perceived technical usability is more dependent on functionalities and design than on popularity of a given app. Among the apps evaluated, Connect SUS and *Dicas de Saúde e Alimentação* obtained higher scores, standing out because of their simplicity; in turn, *My Therapy* presented the lowest relative performance. A gap in the approach to sexual activity was also identified, reflecting health professionals’ discomfort and unpreparedness to discuss the topic^39^ ^,^ ^41^.

Consequently, the findings of this research are in consonance with the literature on mHealth, evidencing the apps’ potential to improve self-management and engagement^30^ ^-^ ^32^ ^,^ ^35^ ^,^ ^53^ ^-^ ^55^. In addition to that, the results can be interpreted from the perspective of theories such as the Transtheoretical Model or the Technology Acceptance Model (TAM), which suggest that ease of use and perceived usefulness exert a direct influence on adherence^44^ ^,^ ^56^. From the practical point of view, a rigorous curation process should precede recommending any given app, as complex interfaces can demotivate aged patients or those with lower digital literacy levels. In addition, the low integration of these systems with electronic medical records can also constitute a barrier for care continuity since, without bidirectional communication between patients’ records and what teams can see, apps remain as isolated resources and miss the opportunity of acting as early warning systems for cardiovascular complications.

In summary, when considering the limitations of the systematic review stage of this study, it is necessary to acknowledge the remarkable heterogeneity across the interventions and the selection bias inherent to using technologies. Most of the studies included populations with previous access to smartphones, which limits generalizing the findings to patients with lower digital literacy levels or more severe clinical frailty. In addition to that, the follow-up times (mostly not exceeding six months) precludes coming to a definite conclusion about whether these new habits are maintained in the long term. Besides, the variation in the apps’ functionalities (ranging from simple SMS reminders to complex gamification and coaching systems) hinders identifying the essential technological component for successful secondary prevention.

The limitations found in the second study stage include having used a convenience sample, which implies the sampling bias inherent to this strategy, in addition to the small number of participants. Having restricted the sample exclusively to hospitalized patients may have underestimated usability barriers and engagement challenges faced by outpatients in their everyday home contexts. In addition, the absence of tests with devices using the iOS system (Apple Store^®^) and the fact that the selection process was restricted to the best rated apps in the online stores preclude representing the totality of options available in the market. In addition to that, the brief use period may not reflect long-term use. External factors such as already knowing the technologies may also have interfered in the answers.

Finally, future research studies should include clinical trials with cost-effectiveness assessments and practical implementation studies, aiming to identify safer and more sustainable tools for cardiovascular self-management.

## Conclusion

In synthesis, the evidence shows that mobile technologies emerge as a coadjuvant strategy with high potential in CCS secondary prevention, presenting favorable results in promoting adherence to drug therapies, controlling risk factors and changing lifestyles. Employing these tools strengthens self-management and ongoing monitoring, filling the assistance gap between in-person consultations. However, the remarkable heterogeneity of functionalities and the predominance of short-term follow-ups indicate that technology should not be considered as an isolated solution to reduce complex clinical outcomes. For the clinical practice, it is concluded that successful mHealth implementation depends on its integration to hybrid care models in which digital devices work as a support element for health surveillance and education. That integration allows multiprofessional teams to target more intensive in-person interventions to the patients that fail to attain the therapeutic targets signaled in the system, optimizing care safety and customization.

## Data Availability

The dataset of this article is available on the RLAE page in the SciELO Data repository, at the link https://doi.org/10.48331/SCIELODATA.CR4I8U
