# Supplementary material for: Effectiveness of mobile apps in the secondary prevention of Chronic Coronary Syndrome: a systematic review and usability analysis
Source: Rev Lat Am Enfermagem. 2026 Nov 13;34:e4982. doi: 10.1590/1518-8345.8117.4982 (PMC13762694; doi:10.1590/1518-8345.8117.4982)
Supplement: Translation

# Translation: Eficácia de aplicativos móveis na prevenção secundária da síndrome coronariana crônica: revisão sistemática e análise de usabilidade^*^

**DOI:** 10.1590/1518-8345.8117.4983

**Authors:** Taís Lins Severo da Silva, Gabriela Aparecida de Andrade Silva, Ligia Cristina Câmara Cunha, Vinicius Batista Santos, Juliana de Lima Lopes

**Affiliations:** 1 Universidade Federal de São Paulo, Escola Paulista de Enfermagem, São Paulo, SP, Brazil.

**Keywords:** Aplicativos Móveis, Acessibilidade aos Serviços de Saúde, Autocuidado, Doença Coronariana, Estilo de Vida Saudável, Prevenção Secundária.

## Abstract

**Objetivo:**

identificar as evidências sobre a eficácia do uso de aplicativos móveis como estratégia para a manutenção de comportamentos saudáveis em pacientes com síndrome coronariana crônica e analisar a usabilidade dos aplicativos mais bem avaliados no Google Play^®^, utilizando a *System Usability Scale*.

**Método:**

estudo metodológico realizado em duas etapas. A primeira consistiu em uma revisão sistemática, seguindo as recomendações do JBI. Foi utilizada a ferramenta GRADE para análise da qualidade da evidência dos desfechos. Na segunda, foi realizada a identificação dos aplicativos móveis disponíveis nas duas principais lojas virtuais do Brasil e a análise da usabilidade dos aplicativos mais bem avaliados pelos usuários no Google Play^®^, após a aprovação do comitê de ética em pesquisa.

**Resultados:**

dos 14 artigos, 11 (79%) mostraram eficácia dos aplicativos na promoção do comportamento saudável para prevenção secundária. A qualidade metodológica e o risco de viés dos estudos foram considerados entre moderados a baixos, a depender do desfecho avaliado. Em relação à usabilidade, apenas um foi classificado como mediano, enquanto os demais receberam avaliações boas, excelentes ou melhor imagináveis.

**Conclusão:**

apesar da escassez de estudos sobre a eficácia desses aplicativos, a maioria mostrou resultados positivos, reforçando seu potencial como estratégia para prevenção secundária.

## Destaques

**(1)** Aplicativos móveis mostram eficácia na prevenção secundária da SCC.

**(2)** Usabilidade simples favorece maior aceitação dos aplicativos pelos usuários.

**(3)** Aplicativos com *feedback* e navegação intuitiva obtêm melhores resultados.

**(4)** Falta de orientação sobre atividade sexual é lacuna nos *apps* analisados.

**(5)** Maioria dos aplicativos teve avaliação boa, excelente ou melhor imaginável.

## Introdução

As doenças crônicas não transmissíveis (DCNT), incluindo doenças cardiovasculares (DCV), neoplasias, Diabetes Mellitus (DM) e doenças respiratórias crônicas, constituem as principais causas de morbimortalidade global, gerando impactos expressivos na qualidade de vida e elevados custos aos sistemas de saúde^1^. Estima-se que as DCNT sejam responsáveis por cerca de 41 milhões de mortes anuais, o que corresponde a aproximadamente 70% dos óbitos mundiais e a mais de 700 mil mortes no Brasil^1^ ^-^ ^2^.

Dentre as DCNT, as DCV destacam-se como a principal causa de internações e óbitos, respondendo por aproximadamente 17,9 milhões de mortes anuais no mundo^3^ ^-^ ^4^. No contexto brasileiro, em 2023, as DCV foram responsáveis por quase 1,3 milhão de internações no Sistema Único de Saúde (SUS) e cerca de 107 mil óbitos, demandando altos recursos do sistema público^5^ ^-^ ^7^.

Para enfrentar esse panorama, a Organização Mundial da Saúde (OMS) elaborou o Plano de Ação Global para Doenças Não Transmissíveis (2013-2030), visando reduzir em um terço as mortes prematuras por DCNT até 2030, além de ampliar o acesso aos serviços de prevenção, tratamento e reabilitação^8^. Entre as metas específicas, destacam-se o aumento de 30% no tratamento medicamentoso e aconselhamento para prevenção secundária das DCV, bem como a expansão de 50% na cobertura de programas de aconselhamento para doenças cardiovasculares e pulmonares^8^. Tais metas convergem com os Objetivos de Desenvolvimento Sustentável (ODS) da Organização das Nações Unidas (ONU), que priorizam a promoção da saúde e do bem-estar por meio da prevenção e do tratamento eficaz^9^.

Uma das doenças que mais impactam na mortalidade prematura por DCNT é a síndrome coronariana crônica (SCC), definida como a manifestação estável da doença arterial coronariana^10^. A SCC está associada a diversos fatores de risco, tornando a prevenção secundária, fundamentada no controle pressórico e lipídico, na adesão terapêutica e na adoção de hábitos saudáveis, essencial para reduzir complicações isquêmicas^3^ ^,^ ^10^. Atualmente, o desenvolvimento de tecnologias digitais voltadas à educação em saúde e ao autocuidado tem se mostrado promissor para prevenção secundária de DCNT. Entre elas, os aplicativos móveis destacam-se como ferramentas eficazes para o monitoramento da saúde e para a promoção de comportamentos saudáveis^11^ ^-^ ^18^. Entretanto, a maior parte dessas soluções concentra-se em condições como DM, obesidade e asma, nas quais já mostram impacto positivo no engajamento e na mudança comportamental^11^ ^,^ ^14^ ^-^ ^18^.

No âmbito das doenças cardiovasculares, as intervenções em *mHealth*, incluindo o uso de aplicativos, têm se voltado predominantemente ao manejo de fatores de risco isolados ou à insuficiência cardíaca, com menor atenção à SCC^16^ ^-^ ^18^. Ressalta-se que as pessoas com SCC enfrentam desafios adicionais para o engajamento às atividades de prevenção secundária, como: complexidade do tratamento, fadiga, dispneia e medo de novos eventos isquêmicos, fatores que podem desestimular a prática de atividade física e a adesão ao tratamento. Ainda, estes indivíduos frequentemente apresentam quadros de ansiedade e depressão, que interferem negativamente na autoeficácia percebida e, consequentemente, na adesão às medidas de prevenção secundária^3^ ^,^ ^16^. Ademais, somam-se a isso o baixo suporte social e a crença de que apenas a farmacoterapia é suficiente, o que compromete o autocuidado integral^3^ ^,^ ^16^.

Assim, torna-se necessário conhecer os aplicativos disponíveis, bem como os estudos que os analisaram, identificando as melhores fundamentações teóricas para a autogestão do cuidado, como a Teoria do Comportamento Planejado e o Modelo Transteórico, além das funcionalidades e da usabilidade relacionadas à melhora da adesão às medidas de prevenção secundária^16^ ^-^ ^25^. Diante desse cenário, o objetivo deste estudo é combinar uma revisão sistemática da literatura, destinada a analisar as evidências sobre a eficácia de aplicativos na adoção e manutenção de medidas de prevenção secundária da SCC, com a análise de usabilidade das ferramentas que foram mais bem avaliadas e que estão disponíveis no Google Play^®^. Ao analisar simultaneamente efetividade e usabilidade, busca-se identificar os elementos que favorecem o engajamento e o autocuidado, ampliando o potencial de aplicação dessas tecnologias na prática clínica, particularmente na educação em saúde mediada pelo enfermeiro.

## Método

### Delineamento do estudo

Este estudo foi conduzido em duas etapas. A primeira consistiu em uma revisão sistemática de literatura de ensaios clínicos randomizados, seguindo as diretrizes do *Preferred Reporting Items for Systematic Reviews and Meta-Analyses* (PRISMA 2020 Statement)^26^. 

A segunda etapa envolveu a identificação dos aplicativos móveis disponíveis nas duas principais lojas virtuais do Brasil, destinados à promoção e à manutenção de hábitos saudáveis em indivíduos com SCC. Em seguida, foi realizada uma análise da usabilidade dos aplicativos mais bem avaliados pelos usuários no Google Play^®^, a fim de verificar sua adequação e facilidade de uso para essa população.

### Cenário em que aconteceu a coleta de dados

A revisão sistemática da literatura foi realizada nas bases de dados MEDLINE/PubMed, SciELO, CINAHL, LILACS e Embase, sem delimitação temporal, de faixa etária ou condições clínicas específicas da SCC. As estratégias de busca combinaram descritores controlados (DeCS/MeSH/Emtree) e termos livres relacionados à SCC, aos aplicativos móveis e à prevenção secundária, utilizando os operadores booleanos AND e OR para ampliar a sensibilidade e a especificidade da busca. Além das bases de dados, realizou-se uma busca em literatura cinzenta, incluindo teses, dissertações e relatórios técnicos, e uma busca manual nas listas de referências dos artigos selecionados, com o objetivo de identificar estudos adicionais potencialmente relevantes.

A identificação dos aplicativos móveis voltados à prevenção secundária da SCC foi realizada nas duas principais lojas virtuais do Brasil, Google Play^®^ e Apple Store^®^. Os aplicativos do Google Play^®^ selecionados foram posteriormente analisados quanto à sua usabilidade em um hospital público terciário, localizado na cidade de São Paulo.

### Período

A revisão sistemática da literatura foi conduzida inicialmente em 5 de fevereiro de 2024 e atualizada em 5 de novembro de 2025, abrangendo as bases eletrônicas previamente definidas. Esta busca foi complementada pela varredura manual das referências dos artigos selecionados e pela análise da literatura cinzenta, em 6 de novembro de 2025. A etapa subsequente, referente à identificação dos aplicativos e à análise de usabilidade, foi realizada no período de fevereiro a agosto de 2024.

### População

A seleção dos aplicativos nas lojas virtuais foi realizada por um dos pesquisadores, enquanto a análise de inclusão ou de exclusão foi conduzida de forma independente por dois outros pesquisadores. Em casos de discordância, um terceiro pesquisador avaliou e definiu a decisão final. Por fim, a usabilidade dos aplicativos selecionados foi analisada por pacientes com SCC.

### Critérios de seleção

A revisão foi orientada pela seguinte questão norteadora: “Quais são as evidências científicas disponíveis sobre o impacto dos aplicativos móveis na manutenção de comportamentos saudáveis voltados à prevenção secundária da doença em pacientes com síndrome coronariana crônica (SCC)?”.

Essa questão foi estruturada com base na estratégia PICO, sendo: P (Paciente) - indivíduos com síndrome coronariana crônica; I (Intervenção) - uso de aplicativos móveis; C (Comparação) - outras estratégias de educação em saúde; e O (Desfecho) - manutenção de comportamentos saudáveis voltados à prevenção secundária da doença.

Foram incluídos estudos publicados em inglês, espanhol e português, que avaliaram a eficácia de aplicativos móveis na promoção de comportamentos saudáveis voltados à prevenção secundária da SCC. As estratégias de busca combinaram descritores controlados (DeCS, MeSH e Emtree) e termos livres, associadas por operadores booleanos (AND, OR, NOT), conforme detalhado na Figura 1.

**Figura 1:** Estratégias de busca de acordo com a base de dados. São Paulo, SP, Brasil, 2024

Base de dados	Estratégia de busca
PubMed	Palavras-chave: (((mobile application[Title/Abstract])) AND ((Coronary Disease[Title/Abstract]) OR Coronary Artery Disease[Title/Abstract] OR Chronic Coronary Syndrome[Title/Abstract])). Descritores de assunto MEsh: (((mobile application[MeSH Terms]) OR (mobile health[MeSH Terms])) AND ((coronary occlusion[MeSH Terms]) OR (Coronary Thrombosis[MeSH Terms]) OR (Coronary Disease[MeSH Terms]) OR (Coronary Artery Disease[MeSH Terms])))
SciELO	Palavras-chave: (((Mobile Application) OR (Aplicativos Móveis) OR (Aplicaciones Móviles) OR (Mobile Health)) AND ((Coronary Disease) OR (Coronary Artery Disease) OR (Doença coronariana) OR (Doença Arterial Coronariana) OR (Enfermedad de la Arteria Coronaria) OR (Enfermedad Coronaria)))
CINAHL	Palavras-chave: (((Mobile Application) OR (Aplicativos Móveis) OR (Aplicaciones Móviles) OR (Mobile Health)) AND ((Coronary Disease) OR (Coronary Artery Disease) OR (Doença coronariana) OR (Doença Arterial Coronariana) OR (Enfermedad de la Arteria Coronaria) OR (Enfermedad Coronaria)))
MEDLINE/LILACS	(mh:”Mobile Applications” OR mh:”Telemedicine” OR tw:”Mobile Application” OR tw:”Mobile Applications” OR tw:”Aplicativo Móvel” OR tw:”Aplicativos Móveis” OR tw:”mHealth” OR tw:”mobile health” OR tw:”smartphone” OR tw:”mobile app”) AND (mh:”Coronary Disease” OR mh:”Coronary Artery Disease” OR tw:”Coronary Disease” OR tw:”Coronary Artery Disease” OR tw:”Doença Arterial Coronariana” OR tw:”Doença Coronariana”) AND (mh:”Secondary Prevention” OR mh:”Life Style” OR tw:”Secondary Prevention” OR tw:”Prevenção Secundária” OR tw:”Healthy Lifestyle” OR tw:”Estilo de Vida Saudável”)
Embase	(‘mobile application’:ab,ti OR ‘aplicativos móveis’:ab,ti) AND (‘coronary disease’:ab,ti OR ‘coronary artery disease’:ab,ti OR ‘chronic coronary syndrome’:ab,ti OR ‘doença coronariana’:ab,ti OR ‘doença arterial coronariana’:ab,ti OR ‘síndrome coronariana crônica’:ab,ti) AND (‘secondary prevention’:ab,ti OR ‘healthy lifestyle’:ab,ti OR ‘prevenção secundária’:ab,ti OR ‘estilo de vida saudável’:ab,ti) AND (‘clinical article’/de OR ‘clinical study’/de OR ‘clinical trial’/de OR ‘comparative effectiveness’/de OR ‘controlled study’/de OR ‘evidence based practice’/de OR ‘major clinical study’/de OR ‘multicenter study’/de OR ‘phase 1 clinical trial’/de OR ‘phase 2 clinical trial’/de OR ‘phase 3 clinical trial’/de OR ‘randomized controlled trial’/de OR ‘randomized controlled trial topic’/de)

Para a seleção dos aplicativos nas lojas virtuais, adotaram-se como critérios de inclusão: serem gratuitos, disponíveis em português e apresentarem funcionalidades para manutenção de comportamentos saudáveis voltados à prevenção secundária da SCC. Foram considerados aqueles que abordavam um ou mais tópicos recomendados pelas diretrizes brasileiras de prevenção secundária, incluindo: adesão ao tratamento medicamentoso, prática de atividade física, alimentação saudável, manejo do estresse, ansiedade e depressão, controle de fatores de risco (pressão arterial, glicemia, colesterol e obesidade), cessação do tabagismo, redução do consumo de álcool, vacinação contra influenza e orientações sobre atividade sexual^10^.

Para a busca, utilizaram-se os termos “doença arterial coronariana”, “síndrome coronariana crônica”, “infarto agudo do miocárdio”, “angina” e “prevenção de doenças cardiovasculares”, inseridos na categoria “Medicina” das lojas virtuais Google Play^®^ e Apple Store^®^.

Os critérios de inclusão dos pacientes que participaram da avaliação de usabilidade foram: ausência de déficits cognitivos e/ou visuais registrados em prontuário, alfabetização suficiente para leitura e compreensão das instruções e, por fim, internação em unidades de terapia intensiva ou enfermarias cardiológicas (clínicas ou cirúrgicas) de um hospital público localizado em São Paulo. 

### Definição da amostra

Foram incluídos na pesquisa todos os estudos selecionados na revisão sistemática da literatura e os aplicativos das lojas virtuais que apresentaram concordância entre dois pesquisadores quanto à elegibilidade.

A amostra utilizada para a análise de usabilidade foi definida por conveniência, composta por pacientes internados na unidade coronariana durante o período de coleta de dados. Esta escolha justifica-se pela necessidade de acessar pacientes em um momento crítico de transição do cuidado, o período pré-alta, etapa em que as orientações de prevenção secundária são intensificadas e a introdução de ferramentas de autogestão digital torna-se mais oportuna.

### Coleta de dados

A busca nas bases de dados foi conduzida por um pesquisador enfermeiro, doutor e docente de uma universidade pública brasileira, que realizou a exportação de todos os registros identificados para a plataforma Rayyan^®^ e executou a remoção automática e manual das duplicatas.

Posteriormente, dois revisores independentes, uma estudante do último ano do curso de Enfermagem e uma enfermeira doutoranda, realizaram a triagem dos estudos por meio da leitura dos títulos e resumos, avaliando sua elegibilidade com base nos critérios previamente definidos. Os artigos potencialmente relevantes seguiram para a leitura integral, etapa na qual os mesmos revisores decidiram pela inclusão ou exclusão dos estudos.

Em caso de divergência em qualquer fase do processo de seleção, um terceiro revisor, a mesma docente responsável pela triagem inicial, realizou a avaliação e determinou a decisão final, garantindo a confiabilidade do processo e reduzindo os vieses.

Os estudos incluídos tiveram seus dados extraídos por um dos pesquisadores, seguindo instrumento padronizado previamente elaborado. Os resultados foram sintetizados qualitativamente, assegurando a rastreabilidade das informações e a transparência metodológica.

Na segunda etapa, a busca por aplicativos nas lojas virtuais Google Play^®^ e Apple Store^®^ foi conduzida por um dos pesquisadores. Os aplicativos encontrados foram avaliados de forma independente por outros dois revisores, que definiram a inclusão conforme os critérios previamente estabelecidos. Em caso de discordâncias, um terceiro pesquisador analisou os aplicativos.

Os aplicativos elegíveis foram classificados de acordo com as recomendações presentes nas diretrizes brasileira de prevenção secundária da SCC, contemplando os seguintes domínios: adesão ao tratamento medicamentoso; prática de atividade física; alimentação adequada; redução da ansiedade; controle da obesidade e sobrepeso; cessação do tabagismo; controle da pressão arterial; controle da glicemia; redução do consumo de bebida alcoólica; vacinação contra influenza; educação para o conhecimento da doença cardíaca; e, por fim, controle do colesterol^10^. 

Após a categorização dos aplicativos, foram selecionados para a etapa de análise de usabilidade os dois aplicativos mais bem avaliados pelos usuários em cada uma dessas categorias, considerando simultaneamente a média de estrelas e o número de avaliadores. Essa estratégia foi adotada para reduzir vieses decorrentes de classificações infladas por baixa quantidade de avaliações. Ao final dessa triagem, foram identificados 24 aplicativos no Google Play^®^ e 22 na Apple Store^®^ que atendiam aos critérios definidos.

Os 24 aplicativos provenientes do Google Play^®^ foram encaminhados para a etapa subsequente do estudo, destinada à análise de usabilidade. A decisão de avaliar exclusivamente aplicativos dessa loja virtual foi fundamentada na predominância do sistema operacional Android entre usuários brasileiros, o que confere maior representatividade à amostra e maior aplicabilidade clínica dos resultados. Os aplicativos exclusivos da Apple Store^®^ foram mapeados, porém excluídos desta etapa devido à menor abrangência desse sistema operacional entre a população atendida e a limitações logísticas para acesso aos dispositivos eletrônicos necessários para a testagem.

A análise de usabilidade foi conduzida por um dos pesquisadores, junto a pacientes diagnosticados com SCC, que compuseram a população-alvo do estudo. Todos os participantes receberam instruções padronizadas referentes aos objetivos e procedimentos da pesquisa, assegurando uniformidade na aplicação e minimizando possíveis vieses decorrentes de diferenças de interpretação. Após a leitura e assinatura do Termo de Consentimento Livre e Esclarecido (TCLE), os pacientes foram convidados a manipular os aplicativos em um *tablet*, pelo tempo que julgassem necessário.

Na sequência, os participantes preencheram o instrumento de avaliação de usabilidade. Cada participante avaliou três aplicativos diferentes, e cada aplicativo foi analisado por seis pacientes, em consonância com recomendações da literatura, segundo as quais a avaliação por cinco a oito usuários é suficiente para identificar a maioria dos problemas de usabilidade, garantindo representatividade e viabilidade prática no contexto hospitalar^27^.

### Variáveis do estudo

As variáveis analisadas na revisão sistemática incluíram: nome do autor, ano e país de publicação, objetivo do estudo, tamanho da amostra, estratégia de educação utilizada no grupo controle, nome do aplicativo, desfechos analisados e principais resultados. Já para os aplicativos identificados nas lojas virtuais, as variáveis consideradas foram: título do aplicativo, relação com os tópicos das diretrizes de prevenção secundária da SCC, número de usuários e avaliação média atribuída pelos usuários. Na etapa final do estudo, a variável analisada foi a usabilidade dos aplicativos do Google Play^®^ com as melhores avaliações pelos usuários.

### Instrumentos utilizados para a coleta das informações

O instrumento utilizado para a extração dos dados dos artigos selecionados na revisão sistemática e dos aplicativos selecionados nas lojas virtuais foi elaborado pelos pesquisadores e contemplava as variáveis descritas anteriormente. 

A usabilidade dos aplicativos selecionados foi analisada por meio do *System Usability Scale* (SUS). A SUS, criada em 1986 por John Brooke, permite a avaliação de produtos e serviços, apresentando como vantagem a brevidade na sua aplicação, tanto para o usuário como para o pesquisador. Essa escala é subdividida em três pilares: 1) efetividade: o usuário consegue completar os objetivos; 2) eficiência: quanto esforço e recurso são necessários para completar os objetivos; 3) satisfação: mede se a experiência foi satisfatória. É dividida em 10 perguntas, com avaliações positivas e negativas, utilizando-se uma escala Likert de cinco pontos, sendo 1 (discordo totalmente) a 5 (concordo totalmente)^28^ ^-^ ^29^. O escore final é calculado da seguinte forma: para as afirmações ímpares, é subtraído 1 ponto do escore que o usuário respondeu e, para as afirmações pares, é subtraído o valor do escore da resposta do usuário de 5. Por exemplo, se o usuário assinalou 4 para a pergunta 1 (afirmação ímpar), o valor para essa questão será de 3 e se assinalou 4 para a pergunta 2 (afirmação par), o escore a se considerar para essa resposta será de 1. Após a obtenção do escore individual de cada questão, os valores obtidos são somados e o resultado é multiplicado por 2,5. A usabilidade é classificada em pior imaginável (até 20,5), pobre (de 21 a 38,5), mediano (de 39 a 52,5), bom (de 53 a 73,5), excelente (de 74 a 85,5) ou melhor imaginável (de 86 a 100)^28^ ^-^ ^29^. A tradução e adaptação transcultural da SUS para o português do Brasil foi realizada em 2021 por um grupo de pesquisadores da Universidade Estadual de Campinas (UNICAMP), que constatou adequadas propriedades psicométricas^28^ ^-^ ^29^.

### Tratamento e análise dos dados

Os dados extraídos dos artigos selecionados foram organizados em uma planilha do Microsoft Excel^®^, contendo as variáveis previamente descritas. A análise foi conduzida de forma qualitativa, com o objetivo de identificar padrões e tendências sobre a eficácia do uso de aplicativos móveis na manutenção de comportamentos saudáveis voltados à prevenção secundária da SCC. A avaliação da qualidade metodológica e do risco de viés dos estudos incluídos foi realizada utilizando o sistema *Grading of Recommendations Assessment, Development and Evaluation* (GRADE), que classifica a confiança na evidência com base em critérios de consistência, precisão, direcionalidade e aplicabilidade dos resultados. A avaliação foi realizada de forma independente por dois revisores, e eventuais discordâncias foram solucionadas por consenso. A aplicação do sistema GRADE permitiu graduar a confiança na evidência dos desfechos avaliados como alta, moderada, baixa ou muito baixa, considerando critérios como risco de viés, inconsistência, evidência indireta, imprecisão e viés de publicação. Essa etapa conferiu maior transparência, rigor metodológico e confiabilidade à síntese dos resultados^30^. 

Os estudos foram categorizados de acordo com a estratégia de educação utilizada, o tipo de aplicativo e os desfechos avaliados. A seguir, as informações referentes aos aplicativos foram igualmente organizadas em planilha do Excel^®^, sendo realizadas análises descritivas e quantitativas para verificar sua popularidade, com base no número de usuários e na avaliação média atribuída pelos usuários. Além disso, os aplicativos foram analisados qualitativamente e classificados segundo os tópicos de prevenção secundária da SCC que abordavam.

Para a etapa de análise de usabilidade, os escores obtidos pela SUS foram organizados em planilha do Microsoft Excel^®^ e submetidos a estatística descritiva, incluindo cálculo de médias, desvios-padrão, frequências e porcentagens das categorias de classificação da usabilidade. Adicionalmente, foi realizada uma análise de correlação no Excel, com o objetivo de verificar a relação entre os escores da SUS e as características dos aplicativos avaliados, especificamente: categoria do aplicativo, avaliação média atribuída pelos usuários nas lojas virtuais e número de avaliações registradas. Todas as análises seguiram um nível de significância de 5% (p<0,05).

### Aspectos éticos

O protocolo desta revisão foi registrado em 24 de outubro de 2025, na plataforma PROSPERO (*International Prospective Register of Systematic Reviews*), sob o número de registro CRD420251176142, com o título: “*Effectiveness of Mobile Applications in the Secondary Prevention of Chronic Coronary Syndrome: A Systematic Review and Usability Analysis*”. O registro retrospectivo ocorreu devido à necessidade de atualização do protocolo, incluindo o detalhamento das estratégias de busca e dos critérios de inclusão. Ressalta-se que a revisão foi atualizada após o registro para, assim, garantir a inclusão dos estudos mais recentes e a consistência com o protocolo revisado. Esses ajustes tiveram caráter exclusivamente organizacional e metodológico, sem alterar os desfechos principais, a condução das análises ou a interpretação dos resultados. Dessa forma, o registro tardio reflete a versão final do protocolo adotada para a execução da revisão, assegurando transparência e rigor metodológico.O projeto de pesquisa foi submetido e aprovado pelo Comitê de Ética em Pesquisa (CEP) com Seres Humanos da Universidade Federal de São Paulo, sob parecer nº 6.620.843. A coleta de dados foi iniciada apenas após essa aprovação. 

Os participantes que aceitaram integrar o estudo assinaram o TCLE, em conformidade com as diretrizes éticas vigentes.

## Resultados

### Primeira etapa - revisão sistemática da literatura

*Identificação e seleção dos estudos*

A busca foi realizada nas bases MEDLINE/PubMed, SciELO, CINAHL, LILACS e Embase, complementada por repositórios de registro e busca manual nas referências dos estudos incluídos. A triagem dos estudos foi conduzida na Plataforma Rayyan^®^ de forma independente por dois revisores, por meio da leitura de títulos, resumos e textos completos. O processo de identificação, seleção e inclusão dos estudos está apresentado no fluxograma PRISMA (Figura 2).

**Figura 2:** Fluxograma da seleção dos artigos para a revisão de literatura sistemática. São Paulo, SP, Brasil, 2025

*Características dos estudos incluídos*

A Figura 3 apresenta a síntese das principais características dos estudos incluídos nesta revisão sistemática, contemplando ano de publicação, objetivo, país de realização, delineamento metodológico, tamanho da amostra, intervenções implementadas, desfechos relacionados à prevenção secundária da SCC e principais resultados identificados.

**Figura 3:** Síntese das principais características dos estudos incluídos na revisão sistemática. São Paulo, SP, Brasil, 2025

Autor/Ano	Objetivo	País	Tipo de estudo	Amostra	Intervenções	Desfecho(s)	Principal resultado
Santo K, et al., 2019^31^	Avaliar a eficácia de aplicativos de lembrete de medicação para melhorar a adesão ao tratamento em pacientes com DAC*.	Austrália	Ensaio clínico randomizado com três braços	156 pacientes com DAC* (56 no GC^†^, 54 no GI^‡^1, 53 no GI^‡^ 2). *Idade Média:* 57,9 anos *Sexo:* 87,7% masculino *Comorbidade:* DAC*.	GC^†^: cuidado usual sem aplicativo. GI^‡^1: aplicativo com lembrete básico (lembranças diárias, com funcionalidades simples). GI^‡^2: aplicativo com lembrete avançado (recursos interativos, customizáveis).	Desfechos *- Adesão ao tratamento medicamentoso:* MMAS-8. *- PA* ^*§*^ sistólica: registro digital em repouso, sentado, média de duas medições. *- PA* ^*§*^ diastólica: registro digital em repouso, sentado, média de duas medições. *- LDL-C* ^*∥*^: amostra de sangue em jejum. *- Colesterol total:* amostra de sangue em jejum. Momentos de coleta: *Baseline* e 3 meses após a randomização.	*- Adesão ao tratamento medicamentoso:* o escore médio da MMAS-8 foi significativamente maior (representando maior adesão à medicação) nos grupos experimentais em comparação com o GC^†^ (diferença média 0,47; IC95% 0,12 a 0,82; p=0,008); *- PA* ^*§*^ sistólica: sem diferença entre os grupos (diferença média -0,89; IC95% -6,97 a 5,20; p=0,773); *- PA* ^*§*^ diastólica: sem diferença entre os grupos (diferença média -0,87; IC95% -4,07 a 2,30; p=0,592); *- LDL-C* ^*∥*^: sem diferença entre os grupos (diferença média 0,90; IC95% 0,73 a 1,10; p=0,320); *- Colesterol total:* sem diferença entre os grupos (diferença média 0,00; IC95% -0,31 a 0,30; p=0,990).
Lunde P, et al., 2020^32^	Avaliar o efeito de acompanhamento individualizado via aplicativo móvel por um ano após a reabilitação cardíaca sobre a capacidade de exercício e outros desfechos em pacientes com doença cardíaca.	Noruega	Ensaio clínico randomizado, multicêntrico, com cegamento dos avaliadores	113 pacientes (57 no GI^‡^ e 56 no GC^†^) *Idade Média:* não reportada *Sexo:* não reportado *Comorbidade:* DAC*.	GC^†^: cuidado usual. GI^‡^: aplicativo de *smartphone* com acompanhamento individualizado por 1 ano após reabilitação cardíaca.	Desfechos - *Prática de atividade física*: desempenho do exercício físico, avaliado como tempo até a exaustão, inclinação máxima (%) e velocidade máxima (km/h), pico de VO2^¶^ e a percepção do esforço pela escala Borg no teste cardiopulmonar; hábitos de exercício avaliado por meio de avaliação da média de sessões de exercício por semana no último ano. Uma sessão de exercício foi definida como atividade estruturada com duração mínima de 30 min., que causasse suor e falta de ar e que deixasse o paciente com sensação de que precisaria tomar banho depois. - *Peso corporal:* aferido sem sapatos, usando roupas de exercício, antes do teste cardiopulmonar. *- PA* ^*§*^: aferida em repouso, por meio de medição manual, preferencialmente no braço esquerdo. *- Perfil lipídico*: coleta de amostra de sangue. Momentos de coleta: basal e após um ano da reabilitação cardíaca.	*- Prática de atividade física:* diferença estatisticamente significativa em benefício ao GI^‡^ no pico de VO₂^¶^ entre os grupos após 1 ano (diferença média de +2,2 ml/kg/min; IC95% 0,9-3,5; p<0,001); tempo até exaustão (diferença média 41segundos; IC95% 9-73; p=0,013) e inclinação máxima (diferença média 1,3%; IC95% 0,3-2,4; p=0,014); hábito de exercício (diferença média +0,9 sessões/semana (IC95% 0,4-1,4; p<0,001). - Não houve diferenças estatísticas significativas entre os grupos em peso corporal, PA^§^, perfil lipídico ou outros desfechos*.*
Song Y, et al., 2020^33^	Avaliar a aplicação e o efeito da reabilitação por exercício telemonitorada por *smartphone* entre pacientes com DAC* na China.	China	Ensaio clínico controlado e randomizado, de centro único.	112 Indivíduos com doença arterial coronariana, com idade inferior a 75 anos, ausência de distúrbios físicos ou mentais (56 no GI^‡^ e 56 no GC^†^). *Idade Média:* 54 ± 9 anos *Sexo:* 87% masculino *Comorbidade:* hipertensão, dislipidemia e diabetes mellitus.	GC^†^: tratamento de rotina. GI^‡^: aplicativo de telemonitoramento (MEMRS-CRS) e contato semanal com os pacientes, por meio de mensagens de texto e ligações telefônicas, fornecendo *feedback* sobre o desempenho nos exercícios, dúvidas sobre o uso do dispositivo e ajustes na prescrição de exercícios.	Desfechos - *Prática de atividade física:* avaliado por meio de questionário sobre o hábito regular da prática, a frequência e a duração da sessão e o tipo de exercício, em que foi observado o atendimento das recomendações da 10ª Diretrizes do *American College of Sports Medicine - Valores da PA* ^*§*^: medidas com esfigmomanômetro (manual ou automático) em repouso, antes do teste de exercício. *- Níveis de glicemia*: coleta de amostra de sangue. *- Níveis de colesterol*: coleta de amostra de sangue. Momentos de coleta: basal e após 6 meses de acompanhamento.	- *Prática de atividade física:* favorável ao GE (93,8% versus 77,1%, p=0,021). *- Valores da PA* ^*§*^: não apresenta os dados estatísticos, mas descreve que não houve diferença entre os grupos. *- Níveis de glicemia*: não apresenta os dados estatísticos, mas descreve que não houve diferença entre os grupos. *- Níveis de colesterol*: não apresenta os dados estatísticos, mas descreve que não houve diferença entre os grupos.
Yu C, et al., 2020^34^	Avaliar a eficácia de um aplicativo para *smartphone* no aumento da adesão a medicamentos de prevenção secundária entre pacientes após revascularização coronariana.	China	Ensaio clínico randomizado, controlado e multicêntrico	1000 participantes que se submeteram a revascularização do miocárdio (n=501 no GI^‡^, n=499 no GC^†^) *Idade Média:* 57,28 ± 9,09 anos *Sexo:* 85,5% masculino *Comorbidade:* DAC*.	GC^†^: cuidado usual sem o aplicativo. GI^‡^: acesso a um aplicativo para smartphone desenvolvido especificamente para o estudo, com lembretes de medicação, educação em saúde cardíaca e de fatores de risco e automonitoramento, durante 6 meses.	Desfechos *- Adesão ao tratamento medicamentoso:* MMAS-8 *- PA* ^*§*^: aferida presencialmente; *- IMC* ^**^: obtido a partir do peso e altura; *- Status de tabagismo:* autorrelatado*.* Momentos de coleta: *Baseline* e aos 6 meses após randomização.	*- Adesão ao tratamento medicamentoso:* aumento da proporção de participantes com baixa aderência, mas sem diferença entre os grupos (11,8% no GI^‡^ e 11,7% no GC^†^ (RR=1,005, IC 95% 0,682-1,480; p=1,000). - Não houve diferenças significativas nos desfechos secundários clínicos (PA^§^, IMC**, tabagismo) entre os grupos.
Li Y, et al., 2022^35^	Investigar o efeito de uma intervenção de terapia digital (DT^††^) baseada em aplicativo móvel de autogestão no uso a longo prazo de medicamentos de prevenção secundária em pacientes com doença coronariana.	China	Ensaio clínico piloto randomizado, aberto e unicêntrico	290 pacientes hospitalizados com DAC* (143 no GI^‡^ e 147 no GC^†^) *Idade Média:* não reportada *Sexo:* não reportado *Comorbidade:* DAC*.	GC^†^: seguimento convencional pós-alta (cuidados usuais de hospitalização/ambulatorial) sem o aplicativo. GI^‡^: acompanhamento regular associado a utilização de um aplicativo móvel de autogestão (plano de medicação, plano de intervenção em estilo de vida, protocolo de seguimento, materiais de educação ao paciente e permitia automonitoramento em casa.	Desfechos *- Adesão ao tratamento medicamentoso:* porcentagem de uso de todas as medicações recomendadas por diretriz para prevenção secundária aos 12 meses; porcentagem de adesão aos medicamentos de prevenção secundária aos 6 meses. *- Níveis da PA* ^*§*^: taxa de controle da PA^§^. *- Níveis de colesterol:* coleta de amostra de sangue aos 6 meses e 12 meses. Momentos de coleta: *Baseline*, 6 meses, 12 meses.	*- Adesão ao tratamento medicamentoso:* aos 12 meses, o GI apresentou melhor proporção de utilização de todas as medicações recomendadas comparado ao controle (RR=1,34; IC 95% 1,121,61; p=0,001) *- Níveis da PA* ^*§*^: o GI apresentou maior proporção de participantes que alcançou o controle de PA^§^ aos 12 meses (RR=1,45; IC 95% 1,221,72; p < .001) *- Níveis de colesterol:* o GI^‡^ apresentou maior proporção de participantes que alcançou o LDLC^∥^ < 1,8 mmol/L aos 12 meses (RR=1,40; IC 95% 1,111,75; p=0,004).
Ni Z, et al., 2022^36^	Avaliar a eficácia de uma intervenção de saúde móvel (*mHealth* ^*‡‡*^ ) usando dois aplicativos móveis para melhorar a adesão à medicação e os desfechos de saúde (PA^§^, FC^§§^) em pacientes com DAC*.	China	Ensaio clínico randomizado, paralelo, não-cego, com dois braços	Recrutados: 230 pacientes com doença cardíaca coronária e analisados 196 (103 no GI^‡^ e 93 no GC^†^). *Idade Média:* 61 ± 11 anos *Sexo:* 80,1 % masculino *Comorbidade:* DAC*.	GC^†^: recebeu apenas materiais educativos via aplicativo (*WeChat*) sem lembretes personalizados. GI^‡^: uso de dois *apps*: *WeChat* para receber materiais educativos e o *Message Express* para envio de lembretes personalizados de medicação.	Desfechos *- Adesão ao tratamento medicamentoso: Voils Extent Scale - Níveis da PA* ^*§*^: aferição pessoalmente. Momentos de coleta: *Baseline*, aos 60 dias (fim da intervenção) e aos 90 dias (30 dias após término da intervenção).	*- Adesão ao tratamento medicamentoso:* escore de não adesão medicamentosa apresentou redução significativa no GI^‡^ tanto aos 60 dias (p=0,04) quanto aos 90 dias (p<0,001). - *PA* ^*§*^: A diferença na taxa de variação da PA^§^ sistólica (β=-0,08, p<0,001) e da PA^§^ diastólica (β=-0,05, p=0,004) entre os dois grupos foi estatisticamente significativa, favorecendo o GI^‡^. A diferença na mudança da proporção de participantes com PA sistólica normal entre os dois grupos foi estatisticamente significativa (β=0,01, p=0,02), mas a diferença entre os grupos não foi estatisticamente significativa para a proporção de participantes com PA^§^ diastólica normal (β=0,006, p=0,32).
Boszko M, et al., 2023^37^	Avaliar a eficácia de um aplicativo móvel de saúde para apoiar pacientes após ICP^∥∥^, com foco na redução de ansiedade e estresse, promoção de autocuidado e adesão ao monitoramento clínico.	Polônia	Ensaio clínico randomizado e controlado	67 pacientes pós-ICP^∥∥^ (34 no GI^‡^ e 33 no controle) *Idade Média:* 61 anos *Sexo:* 65 % masculino *Comorbidade:* DAC*, insuficiência cardíaca.	GC^†^: cuidado usual, com orientações presenciais e folheto impresso. GI^‡^: uso do aplicativo móvel *Cardio Patient*, contendo módulos educativos sobre doença coronariana, instruções após-procedimento de ICP^∥∥^, lembretes para autocuidado e consultas, monitoramento de sintomas, diário de saúde e mensagens motivacionais.	Desfechos *- Nível de ansiedade, depressão e estresse:* DASS 21 *Questionnaire*. Momentos de coleta: *Baseline* (antes da alta hospitalar),30 dias e 90 dias após o procedimento.	*- Nível de ansiedade, depressão e estresse:* Não houve diferenças significativas entre os grupos em nenhum dos momentos avaliados.
Beckie TM, et al. 2024^38^	Avaliar os efeitos de uma intervenção *mHealth* ^*‡‡*^ (*HerBeat*) em mulheres com DAC*, comparando-a ao cuidado educacional usual em termos de capacidade de exercício e desfechos relatados pelas pacientes.	Estados Unidos da América	Ensaio clínico piloto e randomizado	47 mulheres com DAC* (23 no grupo *HerBeat*, 24 no grupo de cuidado educacional) *Idade Média:* 61,2 ± 9,1 anos *Sexo:* 100% feminino *Comorbidade:* DAC*.	GC^†^: manual padronizado de reabilitação cardíaca. GI^‡^: *HerBeat*: *smartphone*, associado ao *smartwatch* e *health coach* (intervenção de mudança de comportamento)	Desfechos *- Ansiedade:* autorrelatada; *- Confiança na alimentação:* autoeficácia de comer saudável; *- PA* ^*§*^: aferida presencialmente; *- Circunferência da cintura:* aferida presencialmente; *- Peso corporal:* aferido presencialmente; *- IMC* ^**^: calculado a partir do peso e altura dos participantes; *- Nível de atividade física:* capacidade do exercício por meio do Teste de Caminhada de 6 minutos Momentos de coleta: *Baseline* e após 3 meses de intervenção.	- O GI teve melhora significativa no 6MWT de *baseline* para 3 meses (p= 0,016; tamanho de efeito d=0,558). A diferença entre os grupos aos 3 meses foi de 38 metros, mas não estatisticamente significativa. Do início aos 3 meses, o GI^‡^ apresentou melhorias significativas em: - Nível de ansiedade (p=0,021); - Confiança alimentar (p=0,028); - PA^§^ diastólica (p=0,03) - Circunferência da cintura (p=0,008).
Bernal-Jiménez MÁ, et al; 2024^39^	Avaliar a eficácia de um aplicativo móvel interativo (EVITE) em pacientes com DAC* que passaram por ICP^∥∥^ na adesão ao estilo de vida saudável, adesão terapêutica e controle de fatores de risco cardiovascular.	Espanha	Ensaio clínico randomizado, controlado, com paralelo, cego para estatístico e analisadores, aberto para pacientes devido à natureza da intervenção.	134 pacientes com DAC* submetidos à ICP^∥∥^ com implante de *stent* (67 no GI^‡^ e 67 no controle). *Idade Média:* 59,49 ± 8,97 anos *Sexo:* 71,9% masculino *Comorbidade:* DAC*, hipertensão, diabetes mellitus.	GC^†^: Cuidados usuais, aconselhamento sobre dieta mediterrânea, atividade física, cessação de tabagismo e adesão medicamentosa. GI: Uso do aplicativo por no mínimo 15 min/dia, por 36 semanas. Funcionalidades: educação em estilo de vida saudável e metas terapêuticas baseadas em diretrizes; autocontrole e monitoramento de atividade física, alimentação, PA^§^, tabagismo, peso, glicemia e adesão medicamentosa; motivação através de lembretes automáticos, mensagens personalizadas, gráficos de progresso e cores indicativas de cumprimento de meta.	Desfechos *- Dieta adequada:* adesão à dieta mediterrânea avaliada por meio do *Mediterranean Diet Adherence Score*; frequência de consumo de alimentos (autorrelato). - *Atividade física: Minnesota Physical Activity Survey*, min/semana. - *Tabagismo*: autorrelato; nível de dependência avaliado por meio do teste de dependência de nicotina Fagerström. *- Nível de conhecimento sobre os fatores de risco e estilo de vida saudável*: instrumento validado e elaborado pelos pesquisadores. - *Adesão ao tratamento medicamentoso:* Morisky Green & Levine 4 itens. Momentos de coleta: *Baseline* e 36 semanas após intervenção. Questionários administrados por telefone devido à pandemia de COVID-19.	Os desfechos foram favoráveis ao GI. *- Dieta adequada:* adesão à dieta mediterrânea (escore médio de 11,83±1,74 versus 10,14±2,02, p<0,001); proporção de pacientes com boa adesão à dieta (91% versus 75%; p=0,02). *- Atividade física:* média de 619±318 min/semana versus 472±261 min/semana; p=0,007). *- Cessação do tabagismo:* 76% (GI) versus 42% (GC^†^); p=0,01; dependência de nicotina, avaliada pelo teste de Fagerström foi menor no experimental (0,93±2,29 versus 2,27±2,82; p=0,02). *- Conhecimento de estilo de vida saudável:* também foi superior no GI^‡^ (118,7±2,65 versus 111,25± 9,05; p< 0,001). *- Adesão ao tratamento medicamentoso:* melhora semelhante entre os grupos (84% no *mHealth* ^*‡‡*^ versus 79% no controle; não significativa).
Cruz-Cobo C, et al., 2024^40^	Comparar a eficácia de uma intervenção de saúde móvel (*mHealth* ^*‡‡*^ ), utilizando o aplicativo eMOTIVA, com a dos cuidados habituais, em relação à adesão às diretrizes de reabilitação cardíaca, considerando os aspectos de estilo de vida, fatores de risco cardiovascular e satisfação entre pacientes com síndrome coronariana aguda.	Espanha	Ensaio clínico randomizado e controlado, com desenho de grupos paralelos.	300 pacientes com DAC* submetidos à ICP^∥∥^ com implante de *stent* após um episódio de síndrome coronariana aguda (150 no GI^‡^ e 150 no controle). *Idade Média:* 62,5 ± 8,7 anos *Sexo:* 69,0% masculino *Comorbidade:* DAC*.	GC^†^: Cuidados usuais. GI^‡^: acesso ao *mHealth* ^*‡‡*^ (e-MOTIVA *app*). O aplicativo possui informações científicas para orientar o usuário na conquista das metas terapêuticas recomendadas nas diretrizes clínicas e na manutenção de um estilo de vida saudável.	Desfechos *- Prática de atividade física: International Physical Activity Questionnaire* (IPAQ) e Teste de caminhada de 6 minutos; *- Manutenção de alimentação saudável: Adherence to the Mediterranean diet* e questionário de frequência de consumo na última semana. *- Nível de conhecimento dos fatores de risco* (estresse e ansiedade; PA^§^ elevada; glicemia; colesterol; obesidade): escala elaborada e validada pelos pesquisadores, contendo 24 itens - *Cessação do tabagismo:* Autorrelato e Teste de Fagerström *- Peso corporal e altura para cálculo do índice de massa corporal - IMC* ^**^: aferição presencial; *- Circunferência da cintura:* aferição presencial; *- PA* ^*§*^ sistólica e diastólica e frequência cardíaca: aferição presencial; *- Valores de lipídios (colesterol total, HDL-C* ^*¶¶*^ , LDL-C ^*∥*^ e triglicerídeos), HbA1c ^***^ e glicemia: coleta de amostras de sangue. Momentos de coleta: basal e após 3 e 6 meses de acompanhamento.	Para todos os desfechos com diferença estatisticamente significativa, o GI^‡^ apresentou desempenho significativamente melhor do que o GC^†^. *- Prática de atividade física:* atividade física (min/sem) após 3 meses de seguimento (578,10 +326,14 versus 443,46+278,11, p<0,001) e após seis meses (614,51+332,26 versus 408,40 +274,49, p<0,001); atividade física (METs/semana) após 3 meses de seguimento (1991,74 +1176,71 versus 1490,48 +925,89, p<0,001) e após seis meses (2112,66+1196,67 versus 1372,60 +944,62, p<0,001); horas sentado/semana após 3 meses de seguimento (8,57 +1,89 versus 9,34 +2,13, p=0,002) e após seis meses (8,38 +1,88 versus 9,59 +2,09, p<0,001); teste de caminhada de seis minutos após seis meses (473,49 +102,28 versus 447,25 +93,68, p=0,04). *- Manutenção de alimentação saudável:* adesão a dieta mediterrânea após 3 meses de seguimento (11,63+1,70 versus 9,32+2,55, p<0,001) e após seis meses (11,92+1,70 versus 8,92+2,66, p<0,001); boa adesão a dieta recomendada após 3 meses de seguimento (93,8% versus 67,6%, p<0,001) e após seis meses (94,4% versus 60,3%, p<0,001).
Cruz-Cobo C, et al., 2024^40^	Comparar a eficácia de uma intervenção de saúde móvel (*mHealth* ^*‡‡*^ ), utilizando o aplicativo eMOTIVA, com a dos cuidados habituais, em relação à adesão às diretrizes de reabilitação cardíaca, considerando os aspectos de estilo de vida, fatores de risco cardiovascular e satisfação entre pacientes com síndrome coronariana aguda.	Espanha	Ensaio clínico randomizado e controlado, com desenho de grupos paralelos.	300 pacientes com DAC* submetidos à ICP^∥∥^ com implante de *stent* após um episódio de síndrome coronariana aguda (150 no GI^‡^ e 150 no controle). *Idade Média:* 62,5 ± 8,7 anos *Sexo:* 69,0% masculino *Comorbidade:* DAC*.	GC^†^: Cuidados usuais. GI^‡^: acesso ao *mHealth* ^*‡‡*^ (e-MOTIVA *app*). O aplicativo possui informações científicas para orientar o usuário na conquista das metas terapêuticas recomendadas nas diretrizes clínicas e na manutenção de um estilo de vida saudável.	Desfechos *- Prática de atividade física: International Physical Activity Questionnaire* (IPAQ) e Teste de caminhada de 6 minutos; *- Manutenção de alimentação saudável: Adherence to the Mediterranean diet* e questionário de frequência de consumo na última semana. *- Nível de conhecimento dos fatores de risco* (estresse e ansiedade; PA^§^ elevada; glicemia; colesterol; obesidade): escala elaborada e validada pelos pesquisadores, contendo 24 itens - *Cessação do tabagismo:* Autorrelato e Teste de Fagerström *- Peso corporal e altura para cálculo do índice de massa corporal - IMC* ^**^: aferição presencial; *- Circunferência da cintura:* aferição presencial; *- PA* ^*§*^ sistólica e diastólica e frequência cardíaca: aferição presencial; *- Valores de lipídios (colesterol total, HDL-C* ^*¶¶*^ , LDL-C ^*∥*^ e triglicerídeos), HbA1c ^***^ e glicemia: coleta de amostras de sangue. Momentos de coleta: basal e após 3 e 6 meses de acompanhamento.	*- Nível de conhecimento dos fatores de risco:* após 3 meses de seguimento (116,14+4,23 versus 111,02+6,94, p<0,001) e após seis meses (117,85+3,83 versus 111,00+7,11, p<0,001). - *Cessação do tabagismo:* após 3 meses de seguimento (62,3% versus 53,2%, p=0,35) e após seis meses (65,3% versus 46,8%, p=0,06); dependência à nicotina após 3 meses de seguimento (2,30+2,27 versus 4,14+2,96, p=0,03) e após seis meses (2,18+2,37 versus 3,64+3,02, p=0,10). *- IMC* ^**^: após 3 meses de seguimento (28,33+4,48 versus 28,24+4,40, p=0,86) e após seis meses (28,22+4,69 versus 28,33+4,74, p=0,69). *- Circunferência da cintura:* após 3 meses de seguimento (102,56+11,53 versus 102,22 + 10.35, p=0,80) e após seis meses (101,59+11,87 versus 102,09 +10,80, p=0,72). *- PA* ^*§*^ sistólica: após 3 meses de seguimento (128,96 +15,87 versus 133,27+14,85, p<0,001) e após seis meses (130,00+21,90 versus 135,78 +16,73, p<0,001). *- PA* ^*§*^ diastólica: após 3 meses de seguimento (73,68±9,76 versus 76,05±11,42; *p*=0,06) e após seis meses (117,85+3,83 versus 111,00+7,11, p<0,001). *- FC* ^*§§*^: após 3 meses de seguimento (66,75±8,91 versus 71,93±9,86; *p*<0,001) e após seis meses (68,20±10,13 versus 70,43±10,80; *p*=0,07). *- Perfil lipídico (colesterol total, HDL-C* ^*¶¶*^ , LDL-C ^*∥*^ e triglicerídeos): apresentaram reduções expressivas em ambos os grupos, mas sem diferenças significativas entre eles em nenhum momento*:* após 3 meses de seguimento. *- HbA1c* ^***^: após 3 meses de seguimento (6,61±1,40% versus 6,19±0,84%; *p*=0,25) e após seis meses (6,44±1,01% versus 6,69±1,02%; *p*=0,36). *- Glicemia:* após 3 meses de seguimento (110,58±35,92 mg/dL versus 112,44±34,38 mg/dL; *p*=0,48) e após seis meses (116,48±45,55 mg/dL versus 128,06±55,69 mg/dL; *p*=0,02).
Ghavami M, et al., 2024^17^	Avaliar se o aplicativo móvel *Green Heart*, com módulos de gerenciamento de PA^§^, DLP^†††^ e cessação de tabagismo, melhora do controle de PA^§^ e de lipídios em pacientes com DAC*.	Irã	Ensaio clínico randomizado, paralelo, cegado para os avaliadores de resultados (*singleblinded*), com dois braços	Total de elegíveis: 1.590 pacientes com DAC*, incluindo 1.114 hipertensos e 1.488 com dislipidemia. - Para análise da hipertensão, participaram 545 indivíduos no GC^†^ e 546 no GI^‡^; para a dislipidemia, foram incluídos 728 no controle e 714 no experimental. *Idade Média:* não reportada *Sexo:* não reportado *Comorbidade:* DAC*.	GC^†^: educação tradicional em papel sobre os mesmos tópicos. GE: uso do aplicativo, com três módulos principais (controle de PA^§^ e da DLP^†††^ e a cessação de tabagismo) e a presença de local para automonitoramento, mensagens motivacionais, lembretes, *feedback* automático e educação.	Desfechos *- Controle da PA* ^*§*^: Sucesso no controle da PA^§^ em hipertensos; *- Controle da DLP* ^*†††*^: proporção de pacientes com dislipidemia e que apresentavam o com LDLC^∥^ <70 mg/dL. Momentos de coleta: *baseline* e 6 meses após a análise dos desfechos	*- Controle da PA* ^*§*^: 88,6% dos participantes do GI^‡^ tiveram a PA^§^ controlada versus 78,5% no GC^†^ (OR=2,13, IC 95%: 1,51-3,03, p<0,001). *- Controle da DLP* ^*†††*^: 24,8% dos participantes do GI^‡^ tiveram a PA^§^ controlada versus 16,1% no GC^†^ (OR=1,72, IC 95%: 1,32-2,26, p<0,001).
Ghavami M, et al., 2024^41^	Avaliar o impacto do módulo de cessação de tabagismo do aplicativo móvel “*Green Heart*” sobre mudanças no comportamento de fumantes atuais com DAC*.	Irã	Ensaio clínico randomizado paralelo, com avaliadores cegados (*singleblinded*) e com dois braços	1.590 indivíduos com DAC* elegíveis, dos quais 668 eram fumantes atuais. Foram analisados 332 do GC^†^ e 102 do controle. *Idade Média:* não reportada *Sexo:* não reportado *Comorbidade:* DAC*.	GC^†^: educação tradicional em papel sobre os mesmos tópicos. (tabagismo, risco cardiovascular) e sem o uso do aplicativo. GE: uso do aplicativo “*Green Heart*”, que dispõe de um módulo específico para cessação do tabagismo, incluindo avaliação automatizada da dependência à nicotina, elaboração de um plano personalizado para abandono do tabaco, conteúdos educativos, mensagens motivacionais e *feedback* em tempo real para apoiar a adesão do paciente.	Desfechos *- Cessação do tabagismo:* abstinência contínua por pelo menos 4 semanas durante o acompanhamento; redução do consumo de tabaco (número de cigarros) ou mudança no status de fumante. Momentos de coleta: Basal e 24 semanas após o início das intervenções.	Na análise por intenção de tratar, o GI^‡^ teve taxas significativamente mais altas de cessação do tabagismo e reduções do uso de tabaco comparado ao controle (OR=2,14, IC 95%: 1,163,97; p=0,015).
Waranski M, et al., 2024^42^	Avaliar a eficácia do programa *eHealth* ^*‖*^ personalizado “RehaPlus+” para promover atividade física regular em pacientes com DAC* após fase II de reabilitação.	Alemanha	Ensaio controlado com grupos paralelos	169 pacientes com DAC* pós-*stent* ou revascularização, após reabilitação de fase II (44 no grupo RehaPlus+; 61 no grupo *usual care*) *Idade Média:* não reportada *Sexo:* não reportado *Comorbidade:* DAC*.	GC^†^: cuidado convencional ambulatorial (24 sessões de treinamento de força e resistência de 90 minutos durante 6 meses). GI^‡^: programa eHealth “RehaPlus+” por 24 semanas, mensagens motivacionais duas vezes por semana + suporte de *case manager*.	Desfechos *- Atividade física (minutos/semana) e ADL* ^*‡‡‡*^: questionário *Bewegungs- und Sportaktivität*) Momentos de coleta: basal e seis meses após o seguimento.	- Atividade física (minutos/semana): após 6 meses o grupo RehaPlus+ teve média de 182±208 minutos/semana de atividade física versus 119±175 minutos/semana no controle (p=0,15). - Nível de ADL^‡‡‡^: 442,79±469,1 minutos/semana no grupo RehaPlus+ versus 308,27± 411,52 no controle (p= 0,20).
Xu L, et al., 2024^43^	Explorar se uma intervenção de gamificação baseada em *smartphone* poderia aumentar a participação em atividade física e outros desfechos físicos e psicológicos em pacientes com DAC*.	China	Ensaio clínico randomizado, controlado e com três braços.	108 pacientes com DAC*, alocados em três grupos: 34 participantes no GC^†^, 36 no GI^‡^ individual gamificado e 38 no GI^‡^ equipe (gamificação associado a interação social). *Idade Média:* não reportada *Sexo:* não reportado *Comorbidade:* DAC*.	GC^†^: intervenção padrão (definição de metas de passos via aplicativo, sem gamificação extra) por 12 semanas. GI individual: uso individual do aplicativo com gamificação (metas de passos, recompensa, *feedback*) por 12 semanas. GI^‡^ em equipe: uso da mesma gamificação do individual, associado ao componente social de equipe por 12 semanas.	Desfechos - *Nível de atividade física:* mudança no número de passos diários; proporção de dias em que a meta de passos foi alcançada. *- IMC* ^**^ e circunferência da cintura: aferidos pessoalmente. Momentos de coleta: Basal e após 12 semanas das intervenções.	No GI^‡^ individual, a intervenção gamificada aumentou significativamente os passos diários em comparação ao GC^†^ (+988 passos; IC 95%: 259-1717; p<0,01), efeito que se manteve no seguimento (+819 passos; IC 95%: 24-1613; p<0,01). Além disso, foram observadas diferenças estatisticamente significativas em IMC^**^ e circunferência da cintura entre o GC^†^ e o grupo individual. No GI^‡^ equipe, em que a gamificação incluiu interação social, não houve aumento significativo no nível de atividade física em relação ao GC^†^.

*DAC = Doença Arterial Coronariana; ^†^GC = Grupo Controle; ^‡^GI = Grupo Intervenção; ^§^PA = Pressão Arterial; ^∥^LDL-C = *Low-density Lipoprotein Cholesterol*; ^¶^VO₂ = Consumo máximo de oxigênio; ^**^IMC = Índice de Massa Corporal; ^††^DTx = Terapia Digital; ^‡‡^ *mHealth* = *Mobile Health*; ^§§^FC = Frequência Cardíaca; ^∥∥^ICP = Intervenção Coronária Percutânea; ^¶¶^HDL-C = *High-density Lipoprotein Cholesterol*; ^***^HbA1c = Hemoglobina Glicada; ^†††^DLP = Dislipidemia; ^‡‡‡^ADL = Atividades de Vida Diária

A síntese dos 14 ensaios clínicos randomizados incluídos nesta revisão sistemática evidenciou ampla heterogeneidade quanto às intervenções digitais, desfechos avaliados e métodos de mensuração, porém com tendência geral de resultados positivos em componentes específicos da prevenção secundária da SCC. Observou-se eficácia estatística favorável ao *mHealth* no controle de fatores de risco clínico (54,5%), na adesão ao tratamento medicamentoso (60%) e, de forma mais expressiva, na promoção da atividade física (71,4%), na cessação do tabagismo (100%) e na adesão a dietas saudáveis (100%). Em contrapartida, os desfechos de saúde mental apresentaram resultados mais modestos e incertos (50%). É importante destacar que, dentre as recomendações previstas nas diretrizes brasileiras, três tópicos não foram abordados em nenhum dos estudos analisados: redução do consumo de álcool, vacinação contra influenza e orientações relacionadas à atividade sexual, o que revela lacunas relevantes no desenvolvimento de aplicativos destinados à população com SCC.

A síntese da certeza da evidência para cada desfecho analisado, fundamentada nos domínios do sistema GRADE, está detalhada na Figura 4.

**Figura 4:** Síntese da qualidade da evidência por desfecho segundo metodologia GRADE. São Paulo, SP, Brasil, 2025

Desfecho	Risco de viés	Inconsistência	Evidência indireta	Imprecisão	Viés de publicação	Certeza global
Controle de fatores de risco^17^ ^,^ ^31^ ^-^ ^36^ ^,^ ^38^ ^-^ ^40^ ^,^ ^43^	🟡 Sério*	🟢 Não sério	🟢 Não sério	🟢 Não sério	🟢 Não suspeito	🟡 Moderada
Adesão medicamentosa^31^ ^,^ ^34^ ^-^ ^36^ ^,^ ^39^	🟡 Sério*	🟢 Não sério	🟢 Não sério	🟢 Não sério	🟢 Não suspeito	🟡 Moderada
Atividade física^32^ ^-^ ^33^ ^,^ ^38^ ^-^ ^40^ ^,^ ^42^ ^-^ ^43^	🟡 Sério*	🟡 Sério^†^	🟢 Não sério	🟡 Sério^‡^	🟡 Suspeito^§^	🔴 Baixa
Cessação do tabagismo^34^ ^,^ ^39^ ^-^ ^41^	🟡 Sério*	🟢 Não sério	🟡 Sério^||^	🟢 Não sério	🟢 Não suspeito	🔴 Baixa
Alimentação saudável^39^ ^-^ ^40^	🟡 Sério*	🟢 Não sério	🟡 Sério^||^	🟡 Sério^‡^	🟢 Não suspeito	🔴 Baixa
Ansiedade/depressão/estresse^37^ ^-^ ^38^	🔴 Muito sério^¶^	🟡 Sério^†^	🟡 Sério^||^	🟡 Sério^‡^	🟡 Suspeito^§^	🔴 Baixa
Redução do consumo de álcool	-	-	-	-	-	-
Vacinação contra influenza	-	-	-	-	-	-
Orientações sobre atividade sexual	-	-	-	-	-	-

*Rebaixado pela natureza aberta (não cega) da intervenção *mHealth*; ^†^Rebaixado devido à alta heterogeneidade estatística e clínica (diferentes métodos de intervenção e medida); ^‡^Rebaixado pela inclusão de estudos-piloto com amostras pequenas (n<50) e intervalos de confiança amplos; ^§^Suspeito em desfechos comportamentais devido à falta de registros de protocolos para estudos com resultados positivos em amostras pequenas; ^||^Rebaixado pelo uso de desfechos baseados apenas em autorrelato (*self-report*) sem validação bioquímica ou observação direta; ^¶^Rebaixado em dois níveis devido à combinação de falta de cegamento em desfechos subjetivos e altas taxas de atrito (>20%)

A certeza da evidência, avaliada conforme a metodologia GRADE, variou de moderada a baixa entre os desfechos analisados, conforme sintetizado na Figura 4. O controle de fatores de risco e a adesão medicamentosa apresentaram certeza moderada, sendo rebaixados apenas no domínio do risco de viés devido à natureza aberta (não cega) intrínseca às intervenções de *mHealth*. Em contrapartida, os desfechos de atividade física, cessação do tabagismo, alimentação saudável e saúde mental (ansiedade, depressão e estresse) foram classificados como de baixa certeza.

Esse rebaixamento, por sua vez, deveu-se à alta heterogeneidade (inconsistência), ao uso de medidas por autorrelato, sem utilização de dados quantificáveis, como uso de pedômetro (evidência indireta), e à imprecisão decorrente de estudos piloto com amostras reduzidas. No desfecho de saúde mental, o risco de viés foi considerado muito sério devido à combinação de falta de cegamento e elevadas taxas de atrito (>20%). A análise de cada um dos artigos, por desfecho, pode ser visualizada no material suplementar (disponível em https://doi.org/10.48331/SCIELODATA.CR4I8U).

### Segunda etapa - análise da usabilidade dos aplicativos selecionados

Nesta etapa, inicialmente foi realizada a busca dos aplicativos no Google Play^®^ e na Apple Store^®^, seguindo o método de busca previamente padronizado. Para cada categoria da prevenção secundária da SCC, foi selecionado o aplicativo com melhor avaliação, considerando simultaneamente o número de avaliações dos usuários e a média de estrelas, de modo a reduzir vieses decorrentes de classificações superestimadas ou com poucas avaliações. 

Os aplicativos identificados abordaram, de forma fragmentada, diferentes domínios da prevenção secundária da SCC, incluindo adesão ao tratamento medicamentoso, prática de atividade física, alimentação adequada, controle da pressão arterial e glicemia, cessação do tabagismo, redução do consumo de bebidas alcoólicas, manejo da ansiedade, controle do peso corporal e vacinação contra influenza. De modo geral, os aplicativos relacionados a atividade física, adesão medicamentosa e alimentação saudável apresentaram maior número de avaliações e melhores escores médios em ambas as plataformas.

Destacaram-se aplicativos amplamente utilizados, como *MyTherapy* - Lembrete de Remédios, Alarme de Medicamentos, Strava e *MyFitnessPal*, refletindo maior disseminação e aceitação entre os usuários. Em contraste, domínios como cessação do tabagismo, controle do consumo de álcool, controle da pressão arterial e controle da glicemia apresentaram menor número de aplicativos e menor volume de avaliações, especialmente na Apple Store^®^. No domínio da vacinação contra influenza, identificaram-se predominantemente aplicativos institucionais, como Meu SUS Digital e e-saúde SP.

Não foram identificados aplicativos específicos voltados à atividade sexual e tampouco aplicativos que abordassem, de forma integrada, todos os domínios da prevenção secundária da SCC, evidenciando que as soluções digitais disponíveis se concentram em comportamentos ou fatores de risco isolados.

Após a seleção, os aplicativos disponíveis no Google Play^®^ com as melhores avaliações dos usuários em cada categoria (*My Therapy*; Treino em Casa & Exercícios; Alimente-se - Dieta e Nutrição; Rootd - Alívio da Ansiedade; Treino para Mulheres, Fitness; Parar de Fumar Assistente; Rastreador de Pressão Arterial; *mySugar* - Controle a Diabetes; Balcão de Sobriedade - *EasyQuit*; Meu SUS Digital; Dicas de Saúde e Alimentação; e, por fim, *WeightWar* - Rastreador de Peso) foram analisados, quanto à usabilidade, por 24 pacientes internados com diagnóstico médico de SCC.

Metade dos participantes desta etapa era do sexo masculino, e a maioria (83%) encontrava-se internada por infarto agudo do miocárdio. Além disso, mais da metade (70%) era ex-tabagista, apresentava hipertensão arterial sistêmica (HAS) (83%) e histórico familiar de doença cardiovascular (87%). Por outro lado, a minoria era etilista (7%), tabagista atual (23%) e portadora de DM (30%).

Os aplicativos foram distribuídos aleatoriamente entre os 24 participantes. A avaliação de usabilidade, realizada por meio da SUS, revelou ampla variação nos escores, como demonstrado na Tabela 1.

**Tabela 1:** Escore mínimo e máximo, média, desvio-padrão e a classificação da usabilidade dos aplicativos. São Paulo, SP, Brasil, 2024

Aplicativo	Pontuação mínima e máxima	Média	Desvio-padrão	Classificação SUS*
*My Therapy*	34 - 60	50,0	26,41	Mediano
Treino em Casa & Exercícios	87,5 - 55	69,58	14,78	Bom
Alimente-se - Dieta e nutrição	90 - 47,5	53,33	18,82	Bom
Rootd - Alívio da Ansiedade	90 - 15	54,58	25,02	Bom
Treino para Mulheres, Fitness	100 - 37,5	77,50	27,48	Excelente
Parar de Fumar Assistente	85 - 52,5	72,08	12,39	Bom
Rastreador de Pressão arterial	90 - 57,5	68,33	12,31	Bom
*mySugr* - Controle a diabetes	87,5 - 42,5	69,17	18,55	Bom
Balcão de sobriedade - *EasyQuit*	92,5 - 55	70,0	15,08	Bom
Meu SUS digital	100 - 82,5	89,58	6,60	Melhor imaginável
Dicas de Saúde e Alimentação	100 - 82,5	90,0	10,12	Melhor imaginável
*WeightWar* - Rastreador de peso	100 - 35	79,17	23,96	Excelente

*SUS = *System Usability Scale*

Os pontos fortes da usabilidade dos aplicativos estavam relacionados ao desejo de uso do sistema com frequência e a confiança em usar o sistema. Em contrapartida, a principal fragilidade identificada estava relacionada à complexidade do sistema.

Ao realizar a análise de correlação entre os escores da SUS e as características dos aplicativos (categoria do aplicativo, avaliação média pelos usuários nas lojas virtuais e número de avaliações), observou-se que não houve correlação significativa entre a avaliação média dos usuários e os escores SUS (r=0,12; p=0,58), bem como que o número de avaliações também não apresentou correlação significativa (r=0,08; p=0,69). Por outro lado, a categoria do aplicativo influenciou parcialmente a percepção de usabilidade, sendo que aplicativos de atividade física e alimentação apresentaram escores médios mais altos (aproximadamente 70-80) em comparação com aplicativos de controle de glicemia ou adesão medicamentosa (aproximadamente 50-60). Esses achados indicam que a usabilidade percebida pelos pacientes depende mais das funcionalidades e do *design* do aplicativo do que da popularidade ou avaliação geral nas lojas virtuais.

Além disso, foi realizada uma análise de subgrupos para verificar se características dos participantes influenciavam a percepção de usabilidade. Para isso, foram comparadas as médias dos escores da SUS entre homens e mulheres, entre participantes com e sem hipertensão arterial sistêmica, diabetes mellitus e histórico familiar de doença cardiovascular, bem como entre pacientes internados por infarto agudo do miocárdio e aqueles com outras formas de síndrome coronariana crônica.

Por fim, a análise não identificou diferenças significativas entre os grupos, indicando que as características demográficas e clínicas dos participantes não influenciaram a percepção de usabilidade. Esses achados sugerem que os aplicativos avaliados apresentam desempenho consistente entre diferentes perfis de usuários.

## Discussão

O presente estudo foi conduzido em duas etapas complementares: uma revisão sistemática de ensaios clínicos randomizados (ECR) e uma avaliação de usabilidade de aplicativos disponíveis na loja Google Play^®^, que contemplassem recomendações para a prevenção secundária da síndrome coronariana crônica (SCC). A análise integrada entre as evidências da literatura e as funcionalidades das ferramentas móveis é fundamental para assegurar a efetividade e a segurança das intervenções em saúde digital nesta população.

Apesar do crescimento significativo no número de aplicativos voltados à saúde cardiovascular, observa-se que muitos carecem de fundamentação científica robusta, oferecendo funcionalidades cuja eficácia não possui comprovação clínica. Tal fato foi evidenciado no presente estudo, no qual se constatou que, embora diversos aplicativos enfatizem a adoção de comportamentos saudáveis, poucos ensaios clínicos publicados na literatura validam efetivamente o impacto dessas ferramentas no desfecho clínico.

Além do mais, a revisão sistemática, composta por 14 ECRs, mostrou que, apesar da heterogeneidade quanto às funcionalidades, desfechos avaliados e duração das intervenções, há uma tendência global favorável ao uso de aplicativos para a modificação de comportamentos de saúde e o controle de fatores de risco cardiovasculares. Ainda, observa-se que as intervenções digitais são consistentemente eficazes em domínios de autogestão, como a adesão medicamentosa e a prática de atividade física. Estudos mostram que o uso de lembretes personalizados e planos de autocuidado via aplicativo, não apenas melhora a pontuação em escalas de adesão, mas também reflete em uma maior proporção de pacientes que atingem metas pressóricas e lipídicas em 12 meses^36^. Essa correlação sugere que o suporte via aplicativo pode preencher uma lacuna crítica no período pós-alta, oferecendo um acompanhamento contínuo que o modelo de cuidado tradicional, muitas vezes episódico, não consegue sustentar.

Esses resultados são corroborados por metanálises recentes, como a que avaliou a eficácia de aplicativos na redução de readmissões hospitalares não planejadas em pacientes pós-infarto agudo do miocárdio ou após intervenção coronariana percutânea. Os resultados mostraram que tais intervenções reduziram significativamente as readmissões em comparação com cuidado padrão (RR=0,45; IC 95%: 0,23-0,89; p=0,0219), com benefícios mais expressivos em estudos de seguimento prolongado^44^. Outra metanálise, envolvendo 34 ensaios clínicos, evidenciou que aplicativos móveis podem reduzir a incidência de eventos cardíacos adversos maiores (RR=0,68; p=0,03), a taxa de readmissão hospitalar (RR=0,56; p<0,001), o colesterol total (DMP= -0,19; p=0,03), triglicerídeos (DMP= -0,24; p<0,001) e a circunferência abdominal (DMP = -1,92; p=0,01), além de promover melhorias em escores de ansiedade e depressão. Benefícios adicionais incluíram o aumento da fração de ejeção do ventrículo esquerdo, do VO₂ máximo, da distância percorrida no teste de caminhada de seis minutos e das taxas de adesão medicamentosa^45^.

Apesar desses impactos positivos, a presente revisão evidenciou que a eficácia da ferramenta não é universal para todos os desfechos. Enquanto variáveis comportamentais apresentaram melhorias expressivas, indicadores antropométricos, como o índice de massa corporal e a circunferência da cintura, mostraram-se mais resistentes à intervenção isolada via aplicativo. Assim, na prática clínica, o enfermeiro e a equipe multiprofissional devem utilizar o dispositivo como um suporte que requer supervisão humana, integrando-o ao aconselhamento nutricional e físico presencial. Adicionalmente, deve-se considerar a associação de estratégias como a gamificação para ampliar os resultados. Um estudo que analisou uma intervenção de 12 semanas com elementos de gamificação, incluindo metas de passos, recompensas e *feedbacks*, demonstrou que essa abordagem foi significativamente superior no incremento do número de passos diários em comparação ao grupo que utilizou apenas o aplicativo convencional. Isso sugere, portanto, que a interatividade e o reforço positivo são essenciais para sustentar o engajamento do paciente^43^.

Ressalta-se que a SCC é uma condição complexa, na qual pacientes deparam-se com as múltiplas prescrições e a necessidade de mudanças substanciais no estilo de vida. Essa complexidade contribui para a baixa adesão ao tratamento, tornando fundamental que ferramentas digitais sejam elaboradas com base em teorias de mudança comportamental. Contudo, poucos estudos relatam tal fundamentação teórica; entre os que o fazem, destacam-se as teorias cognitivo-social, do estabelecimento de metas, da autoeficácia e da autodeterminação^46^. A literatura aponta que intervenções mais eficazes são aquelas construídas de forma interativa, culturalmente adaptadas e capazes de fornecer conteúdo educacional e motivacional robusto^47^ ^-^ ^50^.

Outro ponto relevante identificado, tanto na revisão sistemática quanto na análise dos aplicativos disponíveis, é que nenhum deles aborda de forma integral todos os domínios da prevenção secundária da SCC. Ferramentas centradas apenas em um ou dois domínios podem fragmentar o cuidado, deixando de contemplar comportamentos críticos. A maioria dos aplicativos e dos estudos revisados concentra-se em fatores quantificáveis, como pressão arterial e atividade física, por serem comportamentos fáceis de monitorar e mensurar. Essa eficácia em domínios mensuráveis é corroborada por evidências que indicam melhorias na capacidade de exercício e na adesão medicamentosa^40^ ^,^ ^51^. Por outro lado, domínios como alimentação saudável, manejo do estresse e cessação do tabagismo foram explorados com menor frequência e apresentaram resultados inconsistentes, o que pode estar relacionado à complexidade desses comportamentos, que frequentemente exigem suporte clínico presencial ou terapias comportamentais individualizadas^51^ ^-^ ^52^. A falta desses módulos indica que muitos aplicativos ainda não incorporaram as recomendações das diretrizes mais recentes de forma integral, o que pode comprometer a autogestão.

Ademais, quanto à usabilidade analisada na segunda etapa do estudo, observou-se que a maioria dos aplicativos apresentou desempenho de bom a excelente, com *design* amigável e navegação intuitiva. Os problemas técnicos e a complexidade do sistema foram identificados como fatores de abandono, enquanto o *feedback* automático se associou a melhores resultados^31^ ^-^ ^32^. Já a análise de correlação indicou que a avaliação média dos usuários nas lojas virtuais não se correlacionou significativamente com os escores da SUS, sugerindo que a percepção de usabilidade técnica depende mais das funcionalidades e do *design* do que da popularidade do aplicativo. Entre os avaliados, o *Connect* SUS e o Dicas de Saúde e Alimentação obtiveram os maiores escores, destacando-se pela simplicidade, enquanto o *My Therapy* apresentou o menor desempenho relativo. Foi identificada, ainda, uma lacuna na abordagem da atividade sexual, refletindo desconforto e despreparo dos profissionais de saúde em tratar o tema^39^ ^,^ ^41^.

Os achados desta pesquisa estão em consonância com a literatura sobre *mHealth*, evidenciando o potencial de aplicativos em melhorar a autogestão e o engajamento^30^ ^-^ ^32^ ^,^ ^35^ ^,^ ^53^ ^-^ ^55^. Além disso, os resultados podem ser interpretados sob a ótica de teorias como o Modelo Transteórico e o Modelo de Aceitação Tecnológica (TAM), que sugerem que a facilidade de uso e a utilidade percebida influenciam diretamente a adesão^44^ ^,^ ^56^. Do ponto de vista prático, a recomendação de um aplicativo deve ser precedida por uma curadoria rigorosa, pois interfaces complexas podem desmotivar pacientes idosos ou com menor letramento digital. Ainda, a baixa integração desses sistemas com prontuários eletrônicos também constitui uma barreira para a continuidade do cuidado, pois sem a comunicação bidirecional entre o registro do paciente e a visualização da equipe, o aplicativo permanece como um recurso isolado, perdendo a oportunidade de servir como sistema de alerta precoce para complicações cardiovasculares.

Enfim, ao considerar as limitações da etapa de revisão sistemática deste estudo, é preciso reconhecer a acentuada heterogeneidade das intervenções e o viés de seleção inerente ao uso de tecnologias. A maioria dos estudos incluiu populações com acesso prévio a smartphones, o que limita a generalização dos achados para pacientes com menor letramento digital ou maior fragilidade clínica. Além disso, o tempo de seguimento, que em grande parte não ultrapassou os seis meses, impede a conclusão definitiva sobre a manutenção desses novos hábitos a longo prazo. A variação nas funcionalidades dos aplicativos, que vão desde simples lembretes de SMS até sistemas complexos de gamificação e *coaching*, dificulta a identificação do componente tecnológico essencial para o sucesso da prevenção secundária.

As limitações da segunda etapa do estudo incluem a utilização de uma amostra por conveniência, o que acarreta um viés de amostragem inerente a essa estratégia, além do reduzido número de participantes. A restrição da amostra a pacientes exclusivamente internados pode subestimar barreiras de usabilidade e desafios de engajamento enfrentados por pacientes ambulatoriais no contexto do cotidiano domiciliar. Adicionalmente, a ausência de testes com dispositivos do sistema iOS (Apple Store^®^) e a seleção restrita aos aplicativos melhor avaliados nas lojas virtuais impedem a representação da totalidade de opções disponíveis no mercado. Além disso, o período curto de uso pode não refletir o uso a longo prazo. Fatores externos, como familiaridade prévia com tecnologia, também podem ter interferido nas respostas. 

Por fim, pesquisas futuras devem incluir ensaios clínicos com avaliações de custo-efetividade e estudos de implementação prática, visando identificar ferramentas mais seguras e sustentáveis para a autogestão cardiovascular.

## Conclusão

Em síntese, as evidências mostram que as tecnologias móveis constituem uma estratégia coadjuvante de elevado potencial na prevenção secundária da SCC, apresentando resultados favoráveis na promoção da adesão medicamentosa, no controle de fatores de risco e na modificação de estilos de vida. O emprego dessas ferramentas fortalece a autogestão e o monitoramento contínuo, preenchendo o hiato assistencial entre as consultas presenciais. Contudo, a acentuada heterogeneidade das funcionalidades e o predomínio de seguimentos de curto prazo indicam que a tecnologia não deve ser considerada como uma solução isolada para a redução de desfechos clínicos complexos. Para a prática clínica, conclui-se que o sucesso da implementação do *mHealth* depende de sua integração a modelos híbridos de cuidado, nos quais o dispositivo digital atua como suporte à vigilância e à educação em saúde. Essa integração permite que a equipe multiprofissional direcione intervenções presenciais mais intensivas aos pacientes que não atingem as metas terapêuticas sinalizadas pelo sistema, otimizando a segurança e a personalização do cuidado.

## Data Availability

Declaração de Disponibilidade de Dados: O conjunto de dados deste artigo está disponível na página da RLAE no repositório SciELO Data, no link https://doi.org/10.48331/SCIELODATA.CR4I8U

# Translation: Eficacia de aplicaciones móviles en la prevención secundaria del Síndrome Coronario Crónico: revisión sistemática y análisis sobre su facilidad de uso^*^

**DOI:** 10.1590/1518-8345.8117.4981

**Authors:** Taís Lins Severo da Silva, Gabriela Aparecida de Andrade Silva, Ligia Cristina Câmara Cunha, Vinicius Batista Santos, Juliana de Lima Lopes

**Affiliations:** 1 Universidade Federal de São Paulo, Escola Paulista de Enfermagem, São Paulo, SP, Brazil.

**Keywords:** Aplicaciones Móviles, Accesibilidad a los Servicios de Salud, Autocuidado, Enfermedad Coronaria, Estilo de Vida Saludable, Prevención Secundaria.

## Abstract

**Objetivo:**

identificar la evidencia sobre la eficacia de utilizar aplicaciones móviles como estrategia para mantener comportamientos saludables en pacientes con Síndrome Coronario Crónico y analizar la facilidad de uso de las aplicaciones mejor evaluadas en Google Play^®^, utilizando la *System Usability Scale*.

**Método:**

estudio metodológico realizado en dos etapas. La primera consistió en una revisión sistemática que siguió las recomendaciones del JBI. Se utilizó la herramienta GRADE para analizar la calidad de la evidencia de los desenlaces. En la segunda etapa se identificaron las aplicaciones móviles disponibles en las dos tiendas virtuales más importantes de Brasil y se realizó un análisis sobre la facilidad de uso de las aplicaciones mejor evaluadas por los usuarios en Google Play^®^, luego de la debida aprobación de un comité de ética en investigación.

**Resultados:**

once de los 14 artículos (79%) demostraron la eficacia de las aplicaciones en promover comportamientos saludables para la prevención secundaria. La calidad metodológica y el riesgo de sesgo de los estudios se consideraron de moderados a bajos, dependiendo del desenlace evaluado. En relación con la facilidad de uso, solo una aplicación fue clasificada como promedio, mientras que las demás fueron evaluadas como buenas, excelentes o de lo mejor que pueda imaginarse.

**Conclusión:**

a pesar de la escasez de estudios sobre la eficacia de estas aplicaciones, la mayoría presentó resultados positivos, lo que refuerza su potencial como estrategia para la prevención secundaria.

## Destacados

**(1)** Las aplicaciones móviles demostraron ser eficaces en la prevención secundaria del SCC.

**(2)** La facilidad de uso favorece una mayor aceptación de las aplicaciones entre los usuarios.

**(3)** Las aplicaciones con *feedback* y navegación intuitiva obtuvieron mejores resultados.

**(4)** La ausencia de pautas orientadoras sobre actividad sexual es un déficit en las aplicaciones analizadas.

**(5)** La mayoría de las aplicaciones fueron evaluadas como buenas, excelentes o como de lo mejor que pueda imaginarse.

## Introducción

Las enfermedades crónicas no transmisibles (ECNT), incluidas las enfermedades cardiovasculares (ECV), los neoplasmas, la *Diabetes Mellitus* (DM) y las enfermedades respiratorias crónicas, constituyen la principales causas de morbimortalidad a nivel global, generando significativos efectos sobre la calidad de vida y elevados costos para los sistemas de salud^1^. Se estima que las ECNT son responsables de cerca de 41 millones de muertes al año, lo que corresponde a aproximadamente el 70% de los casos a nivel mundial y a más de 700.000 fallecimientos en Brasil^1^ ^-^ ^2^.

Entre las ECNT, las ECV se destacan como la principal causa de internaciones y muertes, respondiendo por aproximadamente 17,9 millones de fallecimientos por año en todo el mundo^3^ ^-^ ^4^. En el contexto de Brasil, las ECV fueron responsable de casi 1,3 millones de internaciones en el Sistema Único de Salud (SUS) y de cerca de 107.000 muertes en 2023, consumiendo importantes recursos del sistema público^5^ ^-^ ^7^.

A fin de hacer frente a este problema, la Organización Mundial de la Salud (OMS) elaboró el Plan de Acción Global para Enfermedades no Transmisibles (2013-2030), con la intención de reducir en un tercio las muertes prematuras por ECNT antes del año 203, además de ampliar el acceso a servicios de prevención, tratamiento y rehabilitación^8^. Entre las metas específicas se destacan las siguientes: aumento del 30% en el tratamiento medicamentoso y el asesoramiento para la prevención secundaria de las ECV, al igual que una expansión del 50% en la cobertura de programas de asesoramiento para enfermedades cardiovasculares y pulmonares^8^. Dichas metas convergen con los Objetivos de Desarrollo Sostenible (ODS) de la Organización de la Naciones Unidas (ONU), que priorizan la promoción de la salud y el bienestar por medio de la prevención y de tratamientos eficaces^9^.

Una de las enfermedades de mayor impacto sobre la mortalidad prematura por ECNT es el Síndrome Coronario Crónico (SCC), definido como la manifestación estable de la enfermedad arterial coronaria^10^. El SCC está asociado a diversos factores de riesgo; eso convierte a la prevención secundaria, fundamentada en el control de la presión arterial y de los lípidos, en adhesión a las terapias y adopción de hábitos saludables, algo esencial para reducir la cantidad de complicaciones isquémicas^3^ ^,^ ^10^. Actualmente, el desarrollo de tecnologías digitales dirigidas a la educación para la salud y al autocuidado ha demostrado ser promisorio en la prevención secundaria de las ECNT. Entre ellas, las aplicaciones móviles se destacan como herramientas eficaces para controlar la salud y promover comportamientos saludables^11^ ^-^ ^18^. Sin embargo, la mayor parte de estas soluciones se concentra en patologías como DM, obesidad y asma, en las que demostraron un efecto positivo en materia de compromiso activo y cambio conductual^11^ ^,^ ^14^ ^-^ ^18^.

En el ámbito de las enfermedades cardiovasculares, las intervenciones de *mHealth* (incluidas las aplicaciones) se han volcado predominantemente al manejo de factores de riesgo aislados o a la insuficiencia cardíaca, prestando menor atención al SCC^16^ ^-^ ^18^. Se destaca que las personas con SCC enfrentan desafíos adicionales para comprometerse activamente con las actividades de prevención secundaria, a saber: complejidad de los tratamientos, fatiga, disnea y temor a nuevos eventos isquémicos, factores que pueden desmotivar la práctica de actividad física y la adhesión a los tratamientos. Además, estos individuos frecuentemente presentan cuadros de ansiedad y depresión que interfieren negativamente en la autoeficacia percibida y, en consecuencia, en su adhesión a las medidas de prevención secundaria^3^ ^,^ ^16^. También se suma a lo anterior el escaso apoyo social y la creencia de que la farmacoterapia es suficiente, lo que pone en riesgo el autocuidado integral^3^ ^,^ ^16^.

De esta manera, resulta necesario conocer las aplicaciones disponibles (al igual que los estudios que las analizan), identificando las mejores fundamentaciones teóricas para el automanejo del cuidado, como la Teoría del Comportamiento Planificado y el Modelo Transteórico, además de las funcionalidad y facilidad de uso relacionadas con mejorar la adhesión a las medidas de prevención secundaria^16^ ^-^ ^25^. Dado este escenario, el objetivo de este estudio es combinar una revisión sistemática de la literatura (destinada a analizar las evidencias sobre la eficacia de aplicaciones en la adopción y el mantenimiento de medidas de prevención secundaria para el SCC) con un análisis sobre la facilidad de uso de las herramientas mejor evaluadas disponibles en Google Play^®^. Al analizar simultáneamente la efectividad y la facilidad de uso, se pretende identificar los elementos que favorecen el compromiso activo y el autocuidado, ampliando el potencial de aplicación de estas tecnologías en la práctica clínica, particularmente en la educación sanitaria mediada por enfermeros.

## Método

### Diseño del estudio

Este estudio se condujo en dos etapas. La primera consistió en una revisión sistemática de la literatura con ensayos clínicos aleatorizados siguiendo las pautas especificadas en *Preferred Reporting Items for Systematic Reviews and Meta-Analyses* (Declaración PRISMA 2020)^26^.

La segunda etapa implicó identificar las aplicaciones móviles disponibles en las dos tiendas virtuales más importantes de Brasil, destinadas a promover y mantener hábitos saludables en personas con SCC. Subsiguientemente, se analizó la facilidad de uso de las aplicaciones mejor evaluadas por los usuarios en Google Play^®^, a fin de verificar su adecuación y practicidad de uso en esa población.

### Escenario en el que se recolectaron los datos

La revisión sistemática de la literatura se realizó en las bases de datos MEDLINE/PubMed, SciELO, CINAHL, LILACS y Embase, sin restricciones de tiempo, grupo etario o condiciones clínicas específicas del SCC. Las estrategias de búsqueda combinaron descriptores controlados (DeCS/MeSH/Emtree) y términos libres relacionados con el SCC, con las aplicaciones móviles y con la prevención secundaria, utilizando los operadores booleanos AND y OR para mejorar la sensibilidad y especificidad de las búsquedas. Además de las bases de datos, se realizó una búsqueda en la literatura gris, incluyendo tesis, disertaciones e informes técnicos; también se condujo una búsqueda manual en las listas de referencias de los artículos seleccionados con el objetivo de identificar estudios adicionales potencialmente relevantes.

Las aplicaciones móviles dirigidas a la prevención secundaria del SSC se identificaron en las dos tiendas virtuales más importantes de Brasil: Google Play^®^ y Apple Store^®^. Las aplicaciones de Google Play^®^ seleccionadas fueron analizadas posteriormente en cuanto a su facilidad de uso en un hospital público de nivel terciario situado en la ciudad de São Paulo.

### Período

La revisión sistemática de la literatura se condujo inicialmente el 5 de febrero de 2024 y se actualizó el 5 de noviembre de 2025, abarcando las bases de datos electrónicas previamente definidas. Esta búsqueda se complementó el 6 de noviembre de 2025 con una exploración manual de las referencias de los artículos seleccionados y con el análisis de la literatura gris. La etapa subsiguiente (referida a la identificación de aplicaciones y al análisis sobre su facilidad de uso) se realizó de febrero a agosto de 2024.

### Población

Uno de los investigadores seleccionó las aplicaciones en las tiendas virtuales, mientras que el análisis de inclusión o exclusión fue realizado de forma independiente por otros dos investigadores. En caso de desacuerdo, un tercer investigador evaluó la situación y tomó la decisión final. Finalmente, diversos pacientes con SCC analizaron la facilidad de uso de las aplicaciones seleccionadas.

### Criterios de selección

La revisión se basó en la siguiente pregunta orientadora: “¿Cuáles son las evidencias científicas disponibles sobre el efecto de las aplicaciones móviles para mantener comportamientos saludables dirigidos a la prevención secundaria en pacientes con Síndrome Coronario Crónico (SCC)?”.

Esta pregunta se estructuró sobre la base de la estrategia PICO, a saber: P (Pacientes) - individuos con Síndrome Coronario Crónico; I (Intervención) - uso de aplicaciones móviles; C (Comparación) - otras estrategias de educación sanitaria; y O (Desenlace, del inglés “*Outcome*”) - mantener comportamientos saludables dirigidos a la prevención secundaria de la enfermedad.

Se incluyeron estudios publicados en inglés, español y portugués que evaluaran la eficacia de aplicaciones móviles para promover comportamientos saludables dirigidos a la prevención secundaria del SCC. Las estrategias de búsqueda combinaron descriptores controlados (DeCS, MeSH y Emtree) y términos libres, asociados por medio de operadores booleanos (AND, OR, NOT), tal como se detalla en la Figura 1.

**Figura 1:** Estrategias de búsqueda para cada base de datos. São Paulo, SP, Brasil, 2024

Base de datos	Estrategia de búsqueda
PubMed	Palabras clave: (((mobile application[Title/Abstract])) AND ((Coronary Disease[Title/Abstract]) OR Coronary Artery Disease[Title/Abstract] OR Chronic Coronary Syndrome[Title/Abstract])). Descriptores de asunto MeSH: (((mobile application[MeSH Terms]) OR (mobile health[MeSH Terms])) AND ((coronary occlusion[MeSH Terms]) OR (Coronary Thrombosis[MeSH Terms]) OR (Coronary Disease[MeSH Terms]) OR (Coronary Artery Disease[MeSH Terms])))
SciELO	Palabras clave: (((Mobile Application) OR (Aplicativos Móveis) OR (Aplicaciones Móviles) OR (Mobile Health)) AND ((Coronary Disease) OR (Coronary Artery Disease) OR (Doença coronariana) OR (Doença Arterial Coronariana) OR (Enfermedad de la Arteria Coronaria) OR (Enfermedad Coronaria)))
CINAHL	Palabras clave: (((Mobile Application) OR (Aplicativos Móveis) OR (Aplicaciones Móviles) OR (Mobile Health)) AND ((Coronary Disease) OR (Coronary Artery Disease) OR (Doença coronariana) OR (Doença Arterial Coronariana) OR (Enfermedad de la Arteria Coronaria) OR (Enfermedad Coronaria)))
MEDLINE/ LILACS	(mh:”Mobile Applications” OR mh:”Telemedicine” OR tw:”Mobile Application” OR tw:”Mobile Applications” OR tw:”Aplicativo Móvel” OR tw:”Aplicativos Móveis” OR tw:”mHealth” OR tw:”mobile health” OR tw:”smartphone” OR tw:”mobile app”) AND (mh:”Coronary Disease” OR mh:”Coronary Artery Disease” OR tw:”Coronary Disease” OR tw:”Coronary Artery Disease” OR tw:”Doença Arterial Coronariana” OR tw:”Doença Coronariana”) AND (mh:”Secondary Prevention” OR mh:”Life Style” OR tw:”Secondary Prevention” OR tw:”Prevenção Secundária” OR tw:”Healthy Lifestyle” OR tw:”Estilo de Vida Saudável”)
Embase	(‘mobile application’:ab,ti OR ‘aplicativos móveis’:ab,ti) AND (‘coronary disease’:ab,ti OR ‘coronary artery disease’:ab,ti OR ‘chronic coronary syndrome’:ab,ti OR ‘doença coronariana’:ab,ti OR ‘doença arterial coronariana’:ab,ti OR ‘síndrome coronariana crônica’:ab,ti) AND (‘secondary prevention’:ab,ti OR ‘healthy lifestyle’:ab,ti OR ‘prevenção secundária’:ab,ti OR ‘estilo de vida saudável’:ab,ti) AND (‘clinical article’/de OR ‘clinical study’/de OR ‘clinical trial’/de OR ‘comparative effectiveness’/de OR ‘controlled study’/de OR ‘evidence based practice’/de OR ‘major clinical study’/de OR ‘multicenter study’/de OR ‘phase 1 clinical trial’/de OR ‘phase 2 clinical trial’/de OR ‘phase 3 clinical trial’/de OR ‘randomized controlled trial’/de OR ‘randomized controlled trial topic’/de)

Se adoptaron los siguientes criterios de inclusión para seleccionar aplicaciones en las tiendas virtuales: debían ser gratuitas, estar disponibles en portugués y presentar funcionalidades para mantener comportamientos saludables dirigidos a la prevención secundaria del SCC. Se consideraron las que trataban al menos uno de los temas recomendados por las pautas brasileñas de prevención secundaria, a saber: adhesión al tratamiento medicamentoso; práctica de actividad física; alimentación saludable; manejo del estrés, la ansiedad y la depresión; control de factores de riesgo (presión arterial, glicemia, colesterol y obesidad); dejar de fumar; reducir el consumo de alcohol; vacunación contra la gripe; y pautas sobre actividad sexual^10^.

Para la búsqueda se utilizaron los siguientes términos: “*doença arterial coronariana*” (“enfermedad de las arterias coronarias”), “*síndrome coronariana crônica*” (“síndrome coronario crónico”), “*infarto agudo do miocárdio*” (“infarto agudo de miocardio”), “angina” y “*prevenção de doenças cardiovasculares*” (“prevención de enfermedades cardiovasculares”), todos incluidos en la categoría “Medicina” de las tiendas virtuales Google Play^®^ y Apple Store^®^.

A su vez, los criterios de inclusión para los pacientes que participaron en la evaluación de la facilidad de uso fueron los siguientes: ausencia de déficits cognitivos y/o visuales registrados en la historia clínica; suficiente nivel de alfabetización para leer y comprender las instrucciones; e internación en unidades de cuidados intensivos o salas de Cardiología (clínicas o quirúrgicas) de un hospital público situado en São Paulo.

### Definición de la muestra

En el trabajo de investigación se incluyeron todos los estudios seleccionados en la revisión sistemática de la literatura y las aplicaciones de las tiendas virtuales que presentaran concordancia entre dos investigadores en cuanto a su elegibilidad.

La muestra utilizada para el análisis sobre la facilidad de uso se definió por conveniencia y estuvo compuesta por pacientes internados en la unidad coronaria durante el período de recolección de datos. Esta elección se justificó por la necesidad de acercarse a pacientes en un momento crítico del cuidado de transición, el período previo al alta, una etapa en la que se intensifican las pautas de prevención secundaria y resulta más oportuno incorporar herramientas digitales de automanejo.

### Recolección de datos

La búsqueda en las bases de datos estuvo a cargo de un enfermero investigador, con Doctorado y docente de una universidad pública brasileña, que exportó todos los registros identificados a la plataforma Rayyan^®^ y eliminó automática y manualmente los elementos duplicados.

Posteriormente, dos revisores independientes (una estudiante del último año de la carrera de Enfermería y una enfermera que cursaba el Doctorado) cribaron los estudios leyendo sus títulos y resúmenes, evaluando su elegibilidad sobre la base de los criterios previamente definidos. Los artículos potencialmente relevantes avanzaron a la fase de lectura completa, etapa en la que los mismos revisores decidieron si debían ser incluidos o excluidos.

En caso de desacuerdo en cualquier fase del proceso de selección, una tercera revisora (la misma docente responsable por el cribado inicial) evaluó cada situación y tomó la decisión final, garantizando así la confiabilidad del proceso y reduciendo los sesgos.

Uno de los investigadores extrajo los datos de los estudios incluidos, siguiendo un instrumento estandarizado elaborado previamente. Los resultados se sintetizaron cualitativamente, asegurando la rastreabilidad de toda la información y la transparencia metodológica.

En la segunda etapa, uno de los investigadores buscó aplicaciones en las tiendas virtuales Google Play^®^ y Apple Store^®^. Las aplicaciones encontradas fueron evaluadas de forma independiente por otros dos revisores, que definieron su inclusión conforme a los criterios previamente establecidos. En caso de desacuerdo, un tercer investigador analizó las aplicaciones.

Las aplicaciones elegibles se clasificaron de acuerdo con las recomendaciones incluidas en las pautas brasileñas para la prevención secundaria del SCC, contemplando los siguientes dominios: adhesión al tratamiento medicamentoso; práctica de actividad física; alimentación adecuada; reducción de la ansiedad; control de la obesidad y el sobrepeso; dejar de fumar; control de la presión arterial; control de la glicemia; reducir el consumo de bebidas alcohólicas; vacunación contra la gripe; educación sobre enfermedades cardíacas; y control del colesterol^10^.

Después de categorizar las aplicaciones, las dos aplicaciones mejor evaluadas por los usuarios en cada una de esas categorías fueron seleccionadas para la etapa del análisis sobre la facilidad de uso, considerando simultáneamente el promedio de estrellas y la cantidad de evaluadores. Se adoptó esta estrategia para reducir sesgos resultantes de calificaciones sobrestimadas debido a la escasa cantidad de evaluaciones. Al finalizar este cribado se identificaron 24 aplicaciones en Google Play^®^ y 22 en Apple Store^®^ que cumplían con los criterios definidos.

Por lo tanto, las 24 aplicaciones provenientes de Google Play^®^ avanzaron a la etapa subsiguiente del estudio, destinada al análisis sobre la facilidad de uso. La decisión de evaluar exclusivamente aplicaciones de esta tienda virtual se fundamentó en el predominio del sistema operativo Android entre usuarios brasileños, lo que confiere mayor representatividad a la muestra y mejor aplicabilidad clínica de los resultados. Se mapearon las aplicaciones exclusivas de Apple Store^®^; sin embargo, se las excluyó de esta etapa por el menor alcance de este sistema operativo entre la población en cuestión y debido a limitaciones logísticas para acceder a los dispositivos electrónicos necesarios para las pruebas.

Además, el análisis sobre la facilidad de uso estuvo a cargo de uno de los investigadores, quien lo aplicó a pacientes diagnosticados con SCC que conformaron la población objetivo del estudio. Todos los participantes recibieron instrucciones estandarizadas referidas a los objetivos y procedimientos de la investigación, garantizando así uniformidad en la aplicación y minimizando posibles sesgos resultantes de diferencias interpretativas. Una vez leído y firmado el Formulario de Consentimiento Libre e Informado (FCLI), se invitó a los pacientes a que manipularan las aplicaciones en una *tablet* durante el tiempo que creyeran necesario.

Subsiguientemente, los participantes completaron el instrumento de evaluación de la facilidad de uso. Cada participante evaluó tres aplicaciones diferentes, y cada aplicación fue analizada por seis pacientes, en consonancia con las recomendaciones de la literatura, que indican que una evaluación realizada por cinco a ocho usuarios es suficiente para identificar la mayoría de los problemas relacionados con la facilidad de uso, garantizando representatividad y viabilidad práctica en el contexto hospitalario^27^.

### Variables del estudio

Las variables analizadas en la revisión sistemática fueron las siguientes: nombre del autor; año y país de publicación; objetivo del estudio; tamaño de la muestra; estrategia educativa utilizada en el Grupo Control; nombre de la aplicación; desenlaces analizados; y resultados principales. A su vez, en el caso de las aplicaciones identificadas en las tiendas virtuales, las variables consideradas fueron: título de la aplicación; relación con los temas incluidos en las pautas para la prevención secundaria del SCC; cantidad de usuarios; y calificación promedio asignada por los usuarios. La variable que se analizó en la etapa final del estudio fue la facilidad de uso de las aplicaciones de Google Play^®^ mejor evaluadas por los usuarios.

### Instrumentos utilizados para recolectar la información

El instrumento empleado para extraer los datos de los artículos seleccionados en la revisión sistemática y de las aplicaciones seleccionadas en las tiendas virtuales fue elaborado por los investigadores e incluía las variables que se acaban de describir.

La facilidad de uso de las aplicaciones seleccionadas se analizó por medio de la *System Usability Scale* (SUS). Creada en 1986 por John Brooke, la escala SUS permite evaluar productos y servicios, con la ventaja de que su aplicación no requiere mucho tiempo, tanto para usuarios como para investigadores. Esta escala está dividida en tres pilares: 1) Efectividad: el usuario logra completar los objetivos; 2) Eficiencia: el esfuerzo y los recursos necesarios para completar los objetivos; y 3) Satisfacción: mide si la experiencia fue satisfactoria. Está dividida en 10 preguntas, con evaluaciones positivas y negativas y siguiendo una escala Likert de cinco puntos: de 1 (Totalmente en desacuerdo) a 5 (Totalmente de acuerdo)^28^ ^-^ ^29^. La puntuación final se calcula de la siguiente manera: en el caso de las afirmaciones impares se resta 1 punto de la puntuación indicada por el usuario; en cambio, para las afirmaciones pares se resta el valor de la puntuación en la respuesta del usuario de 5. Por ejemplo: si un usuario asigna 4 puntos a la pregunta 1 (afirmación impar), el valor para esa pregunta será 3; en cambio, si indica 4 puntos en la pregunta 2 (afirmación par), la puntuación que se considerará para esa respuesta será 1. Una vez obtenida la puntuación individual de cada pregunta, se suman los valores y se multiplica el resultado por 2,5. La facilidad de uso se clasifica como de lo peor que pueda imaginarse (hasta 20,5), pobre (de 21 a 38,5), promedio (de 39 a 52,5), buena (de 53 a 73,5), excelente (de 74 a 85,5) o de lo mejor que pueda imaginarse (de 86 a 100)^28^ ^-^ ^29^. El proceso de traducción y adaptación transcultural de la escala SUS al portugués de Brasil fue realizada en el año 2021 por un grupo de investigadores de la *Universidade Estadual de Campinas* (UNICAMP), verificándose que sus propiedades psicométricas eran adecuadas^28^ ^-^ ^29^.

### Procesamiento y análisis de los datos

Los datos extraídos de los artículos seleccionados se organizaron en una planilla de Microsoft Excel^®^ que contenía las variables antes descritas. El análisis se realizó de forma cualitativa con el objetivo de identificar patrones y tendencias sobre la eficacia de utilizar aplicaciones móviles para mantener comportamientos saludables dirigidos a la prevención secundaria del SCC. La calidad metodológica y el riesgo de sesgo de los estudios incluidos se realizó utilizando el sistema *Grading of Recommendations Assessment, Development and Evaluation* (GRADE), que clasifica la confianza en la evidencia sobre la base de criterios de uniformidad, precisión, direccionalidad y aplicabilidad de los resultados. La evaluación estuvo a cargo de dos revisores que trabajaron de manera independiente y cualquier desacuerdo eventual fue solucionado por consenso. Haber aplicado el sistema GRADE permitió graduar la confianza en la evidencia de los desenlaces evaluados como alta, moderada, baja o muy baja, considerando criterios como riesgo de sesgo, inconsistencia, evidencia indirecta, imprecisión y sesgo de publicación. Esta etapa confirió mayor transparencia, rigor metodológico y confiabilidad a la síntesis de los resultados^30^.

También cabe destacar que los estudios se categorizaron de acuerdo con la estrategia educativa utilizada, el tipo de aplicación y los desenlaces evaluados. Además, toda la información referente a las aplicaciones también se organizó en una planilla Excel^®^, ejecutando análisis descriptivos y cuantitativos para verificar su popularidad sobre la base de la cantidad de usuarios y, en consecuencia, de la calificación promedio asignada por ellos. Las aplicaciones también se analizaron cualitativamente y clasificaron de acuerdo con los temas de prevención secundaria para el SCC que trataban.

Para la etapa del análisis sobre la facilidad de uso, las puntuaciones obtenidas en la escala SUS se organizaron en otra planilla de Microsoft Excel^®^ para someterlos a estadística descriptiva, que incluyó cálculo de valores medios, desviaciones estándar, frecuencias y porcentajes de las categorías de clasificación correspondientes a la facilidad de uso. Además, se realizó un análisis de correlación en Excel con el objetivo de verificar la relación entre las puntuaciones de la escala SUS y las características de las aplicaciones evaluadas, a saber: categoría de la aplicación; calificación promedio asignada por los usuarios en las tiendas virtuales; y cantidad de evaluaciones registradas. En todos los análisis se adoptó un nivel de significancia del 5% (p<0,05).

### Aspectos éticos

El protocolo de esta revisión se registró el 24 de octubre de 2025 en la plataforma PROSPERO (*International Prospective Register of Systematic Reviews*), con código de registro CRD420251176142 y el siguiente título: “*Effectiveness of Mobile Applications in the Secondary Prevention of Chronic Coronary Syndrome: A Systematic Review and Usability Analysis*”. El registro retrospectivo se debió a la necesidad de actualizar el protocolo, incluyendo un detalle de las estrategias de búsqueda y de los criterios de inclusión. Se destaca que la revisión se actualizó después del registro para garantizar que se incluyeran los estudios más recientes y asegurar la consistencia con el protocolo revisado. Estos ajustes tuvieron un carácter exclusivamente organizacional y metodológico, sin alterar los desenlaces principales, el desarrollo de los análisis ni la interpretación de los resultados. De esta manera, el registro tardío refleja la versión final de protocolo que se adoptó para llevar a cabo la revisión, garantizando así transparencia y rigor metodológico.

El proyecto de investigación fue presentado ante y aprobado por el Comité de Ética en Investigación (*Comitê de Ética em Pesquisa*, CEP) con Seres Humanos de la *Universidade Federal de São Paulo*, con número de opinión 6.620.843. La recolección de datos solo se inició una vez obtenida dicha aprobación.

Los participantes que aceptaron integrar el estudio firmaron el FCLI, conforme a las pautas éticas vigentes.

## Resultados

### Primera etapa - Revisión sistemática de la literatura

*Identificación y selección de estudios*

La búsqueda se realizó en las bases de datos MEDLINE/PubMed, SciELO, CINAHL, LILACS y Embase, y se la complementó con repositorios de registros y con una búsqueda manual en las referencias de los estudios incluidos. Dos revisores cribaron los estudios de forma independiente en la Plataforma Rayyan^®^ leyendo sus títulos, resúmenes y textos completos. El proceso de identificación, selección e inclusión de los estudios se presenta en el diagrama de flujo PRISMA (Figura 2).

**Figura 2:** Diagrama de flujo en el que se muestra la selección de artículos para la revisión sistemática de la literatura. São Paulo, SP, Brasil, 2025

*Características de los estudios incluidos*

En la Figura 3 se presenta una síntesis de las principales características de los estudios incluidos en esta revisión sistemática, incluyendo lo siguiente: año de publicación, objetivo, país, diseño metodológico, tamaño de la muestra, intervenciones implementadas, desenlaces relacionados con la prevención secundaria del SCC y principales resultados identificados.

**Figura 3:** Síntesis de las principales características de los estudios incluidos en la revisión sistemática. São Paulo, SP, Brasil, 2025

Autor/Año	Objetivo	País	Tipo de estudio	Muestra	Intervenciones	Desenlace(s)	Resultado principal
Santo K, et al., 2019^31^	Evaluar la eficacia de aplicaciones con recordatorios de medicación para mejorar la adhesión al tratamiento en pacientes con EAC*.	Australia	Ensayo clínico aleatorizado con tres ramas	156 pacientes con EAC* (56 en el GC^†^, 54 en el GI^‡^1, 53 en el GI^‡^2). *Media de edad:* 57,9 años *Sexo:* 87,7% masculino *Comorbilidad:* EAC*.	GC^†^: cuidados usuales sin aplicaciones. GI^‡^1: aplicación con recordatorios básicos (recordatorios diarios, con funciones simples). GI^‡^2: aplicación con recordatorios avanzados (recursos interactivos y personalizables).	Desenlaces *- Adhesión al tratamiento medicamentoso* MMAS-8. *- PA* ^*§*^ sistólica: registro digital en reposo, sentado, promedio de ambas mediciones. *- PA* ^*§*^ diastólica: registro digital en reposo, sentado, promedio de ambas mediciones. *- LDL-C* ^*∥*^: muestra de sangre en ayunas. *- Colesterol total:* muestra de sangre en ayunas. Momentos de recolección: *Baseline* y 3 meses después de la aleatorización.	*- Adhesión al tratamiento medicamentoso* la puntuación media de MMAS-8 fue significativamente más alta (representando mayor adhesión a la medicación) en los grupos experimentales en comparación con el GC^†^ (diferencia media: 0,47; IC95%: 0,12; 0,82; p=0,008); *- PA* ^*§*^ sistólica: sin diferencia entre los grupos (diferencia media: -0,89; IC95%: -6,97; 5,20; p=0,773); *- PA* ^*§*^ diastólica: sin diferencia entre los grupos (diferencia media: -0,87; IC95%: -4,07; 2,30; p=0,592); *- LDL-C* ^*∥*^: sin diferencia entre los grupos (diferencia media: 0,90; IC95%: 0,73; 1,10; p=0,320); *- Colesterol total:* sin diferencia entre los grupos (diferencia media: 0,00; IC95%: -0,31; 0,30; p=0,990).
Lunde P, et al., 2020^32^	Evaluar el efecto ejercido por un seguimiento individualizado a través de una aplicación móvil durante un año después de la rehabilitación cardíaca sobre la capacidad de ejercicio físico y otros desenlaces en pacientes con enfermedades cardíacas.	Noruega	Ensayo clínico aleatorizado, multicéntrico, con cegamiento de evaluadores.	113 pacientes (57 en el GI^‡^ y 56 en el GC^†^) *Media de edad:* no informada *Sexo:* no informado *Comorbilidad:* EAC*.	GC^†^: cuidados usuales. GI^‡^: aplicación para *smartphones* con seguimiento individualizado por 1 año después de la rehabilitación cardíaca.	Desenlaces - *Práctica de actividad física*: rendimiento en el ejercicio físico, evaluado como tiempo transcurrido hasta que la persona se agota, inclinación máxima (%) y velocidad máxima (km/h), pico de VO_2_^¶^ y percepción de esfuerzo según la escala Borg en la prueba cardiopulmonar; hábitos de ejercicio físico evaluados por medio de la media de sesiones de ejercicio por semana en el último año. Cada sesión de ejercicio físico se definió como una actividad estructurada con una duración mínima de 30 minutos, que haga sudar a la persona y deje al paciente con la sensación que deberá darse un baño. - *Peso corporal:* se midió descalzo, con prendas deportivas y antes de la prueba cardiopulmonar. *- PA* ^*§*^: medida en reposo y de forma manual, preferentemente en el brazo izquierdo. *- Perfil de lípidos*: recolección de una muestra de sangre. Momentos de recolección: basal y después de un año de la rehabilitación cardíaca.	*- Práctica de actividad física:* diferencia estadísticamente significativa en beneficio del GI^‡^ en el pico de VO_2_^¶^ entre los grupos después de 1 año (diferencia media de +2,2 ml/kg/minuto; IC95%: 0,9; 3,5; p<0,001); tiempo hasta que la persona se agota (diferencia media: 41 segundos; IC95%: 9; 73; p=0,013) e inclinación máxima (diferencia media: 1,3%; IC95%: 0,3; 2,4; p=0,014); hábito de ejercicio físico (diferencia media: +0,9 sesiones/semana; IC95%: 0,4; 1,4; p<0,001). - No se registraron diferencias estadísticamente significativas entre los grupos en el peso corporal, la PA^§^, el perfil de lípidos u otros desenlaces*.*
Song Y, et al., 2020^33^	Evaluar la aplicación y el efecto de la rehabilitación con ejercicios físicos telemonitorizada por medio de un *smartphone* entre pacientes con EAC* en China.	China	Ensayo clínico controlado y aleatorizado, con un solo centro.	112 personas con enfermedad de las arterias coronarias y menos de 75 años de edad, sin disturbios físicos o mentales (56 en el GI^‡^ y 56 en el GC^†^). *Media de edad:* 54±9 años *Sexo:* 87% masculino *Comorbilidad:* hipertensión, dislipidemia y diabetes *mellitus*.	GC^†^: tratamiento de rutina. GI^‡^: aplicación de telemonitorización (MEMRS-CRS) y contacto semanal con los pacientes por medio de mensajes de texto y llamadas telefónicas, proporcionado *feedback* sobre sobre el desempeño en los ejercicios físicos, subsanando dudas sobre cómo usar el dispositivo e implementando ajustes en la prescripción de ejercicios.	Desenlaces - *Práctica de actividad física:* se evaluó por medio de un cuestionario sobre el hábito periódico, la frecuencia y la duración cada sesión y el tipo de ejercicio, observándose que se cumplía con las recomendaciones de la 10ª edición de las pautas del *American College of Sports Medicine - Valores de PA* ^*§*^: se midieron con un tensiómetro (manual o automático) en reposo, antes de la prueba física. *- Niveles de glicemia*: recolección de muestra de sangre. *- Niveles de colesterol*: recolección de muestra de sangre. Momentos de recolección: basal y después de 6 meses de seguimiento.	- *Práctica de actividad física:* favorable al GE (93,8% versus 77,1%; p=0,021). *- Valores de PA* ^*§*^: no se presentan datos estadísticos, solo se describe que no hubo diferencia entre los grupos. *- Niveles de glicemia*: no se presentan datos estadísticos, solo se describe que no hubo diferencia entre los grupos. *- Niveles de colesterol*: no se presentan datos estadísticos, solo se describe que no hubo diferencia entre los grupos.
Yu C, et al., 2020^34^	Evaluar la eficacia de una aplicación para *smartphones* en mejorar la adhesión a medicamentos de prevención secundaria entre pacientes después de procedimientos de revascularización coronaria.	China	Ensayo clínico aleatorizado, controlado y multicéntrico.	1000 participantes sometidos a revascularización del miocardio (n=501 en el GI^‡^, n=499 en el GC^†^) *Media de edad:* 57,28±9,09 años *Sexo:* 85,5% masculino*Comorbilidad:* EAC*.	GC^†^: cuidados usuales sin aplicaciones. GI^‡^: acceso a una aplicación para *smartphones* desarrollada específicamente para el estudio, con recordatorios de medicación, educación en salud cardíaca y de factores de riesgo y autoseguimiento, durante 6 meses.	Desenlaces *- Adhesión al tratamiento medicamentoso* MMAS-8 *- PA* ^*§*^: se midió en persona; *- IMC* ^**^: se obtuvo a partir del peso y la altura; *- Tabaquismo:* autoinformado*.* Momentos de recolección: *Baseline* y 6 meses después de la aleatorización.	*- Adhesión al tratamiento medicamentoso* mayor proporción de participantes con escasa adhesión, aunque sin diferencia entre los grupos (11,8% en el GI^‡^ y 11,7% en el GC^†^ (RR=1,005; IC 95%: 0,682; 1,480; p=1,000). - No se registraron diferencias significativas en los desenlaces clínicos secundarios (PA^§^, IMC^**^, tabaquismo) entre los grupos.
Li Y, et al., 2022^35^	Investigar el efecto ejercido por una intervención de terapia digital (DTx^††^) basada en una aplicación móvil de automanejo sobre el consumo a largo plazo de medicamentos de prevención secundaria en pacientes con enfermedad coronaria.	China	Ensayo clínico piloto aleatorizado, abierto y con un solo centro.	290 pacientes hospitalizados con EAC* (143 en el GI^‡^ y 147 en el GC^†^) *Media de edad:* no informada *Sexo:* no informado*Comorbilidad:* EAC*.	GC^†^: seguimiento convencional posterior al alta (cuidados usuales de internación/ambulatorios) sin aplicaciones. GI^‡^: seguimiento periódico asociado a utilizar una aplicación móvil de automanejo (plan de medicación, plan de intervención en estilo de vida, protocolo de seguimiento, materiales de educación para el paciente y con posibilidad de automomonitorización en el hogar).	Desenlaces *- Adhesión al tratamiento medicamentoso:* porcentaje de uso de todos los medicamentos recomendados por las pautas de prevención secundaria a los 12 meses; Porcentaje de adhesión a los medicamentos de prevención secundaria a los 6 meses. *- Niveles de PA* ^*§*^: tasa de control de la PA^§^. *- Niveles de colesterol:* recolección de muestras de sangre a los 6 y 12 meses. Momentos de recolección: *Baseline*, 6 meses, 12 meses.	*- Adhesión al tratamiento medicamentoso* a los 12 meses, el GI presentó una mayor proporción de uso de todos los medicamentos recomendados en comparación con el Grupo Control (RR=1,34; IC95%: 1,121; 0,61; p=0,001) *- Niveles de PA* ^*§*^: el GI presentó una mayor proporción de participantes que lograron controlar la PA^§^ a los 12 meses (RR=1,45; IC95%: 1,22; 1,72; p<0,001) *- Niveles de colesterol:* el GI^‡^ presentó una mayor proporción de participantes que alcanzaron valores de LDLC^∥^ <1,8 mmol/L a los 12 meses (RR=1,40; IC95%: 1,11; 1,75; p=0,004).
Ni Z, et al., 2022^36^	Evaluar la eficacia de una intervención de salud móvil (*mHealth* ^*‡‡*^ ) usando dos aplicaciones móviles para mejorar la adhesión a la medicación y los resultados de salud (PA^§^, FC^§§^) en pacientes con EAC*.	China	Ensayo clínico aleatorizado, paralelo, no ciego y con dos ramas.	Sujetos reclutados: 230 pacientes con enfermedad cardíaca coronaria, de los cuales se analizaron 196 (103 en el GI^‡^ y 93 en el GC^†^). *Media de edad:* 61±11 años *Sexo:* 80,1% masculino*Comorbilidad:* EAC*.	GC^†^: solamente recibió materiales educativos a través de la aplicación (*WeChat*), sin recordatorios personalizados. GI^‡^: se usaron dos aplicaciones: *WeChat* para recibir materiales educativos y *Message Express* para enviar recordatorios de medicación personalizados.	Desenlaces *- Adhesión al tratamiento medicamentoso: Voils Extent Scale - Niveles de PA* ^*§*^: se midieron en persona. Momentos de recolección: *Baseline*, a los 60 días (fin de la intervención) y a los 90 días (30 días después de finalizada la intervención.	*- Adhesión al tratamiento medicamentoso* la puntuación correspondiente a la no adhesión a los medicamentos presentó una reducción significativa en el GI^‡^, tanto a los 60 días (p=0,04) como a los 90 días (p<0,001). - *PA* ^*§*^: La diferencia en la tasa de variación de la PA^§^ sistólica (β=-0,08; p<0,001) y de la PA^§^ diastólica (β=-0,05; p=0,004) entre ambos grupos fue estadísticamente significativa, favoreciendo al GI^‡^. La diferencia en el cambio con respecto a la proporción de participantes con PA sistólica normal entre ambos grupos fue estadísticamente significativa (β=0,01; p=0,02); sin embargo, la diferencia entre los grupos no fue estadísticamente significativa para el porcentaje de participantes con PA^§^ diastólica normal (β=0,006; p=0,32).
Boszko M, et al., 2023^37^	Evaluar la eficacia de una aplicación de salud móvil para brindar apoyo a pacientes después de una ICP^∥∥^, enfocándose en reducir la ansiedad y el estrés y en promover el autocuidado y la adhesión a la monitorización clínica.	Polonia	Ensayo clínico aleatorizado y controlado.	67 pacientes ya sometidos a una ICP^∥∥^ (34 en el GI^‡^ y 33 en el Grupo Control) *Media de edad:* 61 años *Sexo:* 65 % masculino*Comorbilidad:* DAC*, insuficiencia cardíaca.	GC^†^: cuidados usuales, con pautas orientadoras en persona y folleto impreso. GI^‡^: se usó la aplicación móvil *Cardio Patient*, que contenía módulos educativos sobre enfermedades coronarias, instrucciones para después del procedimiento de ICP^∥∥^, recordatorios para autocuidado y consultas, monitorización de síntomas, diario de salud y mensajes motivacionales.	Desenlaces *- Niveles de ansiedad, depresión estrés:* Cuestionario DASS-21. Momentos de recolección: *Baseline* (antes del alta hospitalaria),30 y 90 días después del procedimiento.	*- Niveles de ansiedad, depresión estrés:* No se registraron diferencias significativas entre los grupos en ninguno de los momentos evaluados.
Beckie TM, et al. 2024^38^	Evaluar los efectos ejercidos por una intervención *mHealth* ^*‡‡*^ (*HerBeat*) en mujeres con EAC*, comparándola con los cuidados educativos usuales en términos de capacidad de ejercicio físico y desenlaces informados por las pacientes.	Estados Unidos de América	Ensayo clínico piloto y aleatorizado.	47 mujeres con EAC* (23 en el grupo *HerBeat*, 24 en el grupo de cuidados educativos). *Media de edad:* 61,2±9,1 años *Sexo:* 100% femenino*Comorbilidad:* EAC*.	GC^†^: manual estandarizado de rehabilitación cardíaca. GI^‡^: *HerBeat*: *smartphone*, asociada a *smartwatch* y a *health coach* (intervención de cambio conductual).	Desenlaces *- Ansiedad:* autoinformada; *- Confianza en la alimentación:* autoeficacia para comer sano; *- PA* ^*§*^: se midió en persona; *- Circunferencia de la cintura:* se midió en persona; *- Peso corporal:* se midió en persona; *- IMC* ^**^: se calculó a partir del peso y la altura de los participantes; *- Nivel de actividad física:* capacidad de ejercicio físico por medio de la Prueba de caminata de 6 minutos. Momentos de recolección: *Baseline* y después de 3 meses de intervención.	- En el GI se registró una mejora significativa en 6MWT del momento *baseline* a los 3 meses (p=0,016; tamaño del efecto d=0,558). Aunque no fue estadísticamente significativa, la diferencia entre los grupos a los 3 meses fue de 38 metros. Desde el inicio hasta los 3 meses, el GI^‡^ presentó mejoras significativas en los siguientes aspectos: - Nivel de ansiedad (p=0,021); - Confianza en la alimentación (p=0,028); - PA^§^ diastólica (p=0,03) - Circunferencia de la cintura (p=0,008).
Bernal-Jiménez MÁ, et al; 2024^39^	Evaluar la eficacia de una aplicación móvil interactiva (EVITE) en pacientes con EAC* ya sometidos a una ICP^∥∥^ en adoptar un estilo de vida saludable, en la adhesión terapéutica y en el control de factores de riesgo cardiovascular.	España	Ensayo clínico aleatorizado, controlado, paralelo, con estadístico y analizadores cegados y abierto para los pacientes debido a la naturaleza de la intervención.	134 pacientes con EAC* ya sometidos a una ICP^∥∥^ con implante de *stent* (67 en el GI^‡^ y 67 en el Grupo Control). *Media de edad:* 59,49±8,97 años *Sexo:* 71,9% masculino*Comorbilidades:* EAC*, hipertensión, diabetes *mellitus*.	GC^†^: cuidados usuales, asesoramiento sobre la Dieta Mediterránea, actividad física, dejar de fumar y adhesión al tratamiento medicamentoso. GI: se usó la aplicación durante un mínimo de 15 minutos/día, por 36 semanas. Funcionalidades: instrucción sobre estilos de vida saludables y metas terapéuticas basadas en pautas; autocontrol y monitorizacion de la actividad física, la alimentación, la PA^§^, el hábito de fumar, el peso, la glicemia y la adhesión al tratamiento medicamentoso; motivación a través de recordatorios automáticos, mensajes personalizados, gráficos de progreso y colores que indicaban que se había cumplido las metas.	Desenlaces *- Dieta adecuada:* adhesión a la Dieta Mediterránea evaluada por medio del *Mediterranean Diet Adherence Score*; frecuencia de consumo de alimentos (autoinformada. - *Actividad física: Minnesota Physical Activity Survey*, minutos/semana. - *Tabaquismo*: autoinformado; nivel de dependencia evaluado por medio de la prueba de adicción a la nicotina de Fagerström. *- Nivel de conocimiento sobre factores de riesgo y estilos de vida saludables*: instrumento validado y elaborado por los investigadores. - *Adhesión al tratamiento medicamentoso: Morisky Green & Levine* 4 ítems. Momentos de recolección: *Baseline* y 36 semanas después de la intervención. Los cuestionarios se aplicaron por teléfono debido a la pandemia de COVID-19.	Los desenlaces fueron favorables al GI. *- Dieta adecuada:* adhesión a la Dieta Mediterránea (puntuación media de 11,83±1,74 versus 10,14±2,02, p<0,001); proporción de pacientes con buena adhesión a la dieta (91% versus 75%; p=0,02). *- Actividad física:* media de 619±318 minutos/semana versus 472±261 minutos/semana; p=0,007). *- Dejar de fumar:* 76% (GI) versus 42% (GC^†^); p=0,01; evaluada con la prueba Fagerström, la adicción a la nicotina fue menor en el Grupo Experimental (0,93±2,29 versus 2,27±2,82; p=0,02). *- Conocimientos sobre estilos de vida saludables:* también fue superior en el GI^‡^ (118,7±2,65 versus 111,25±9,05; p<0,001). *- Adhesión al tratamiento medicamentoso* la mejora fue similar en ambos grupos (84% en *mHealth* ^*‡‡*^ versus 79% en el Grupo Control, no significativa).
Cruz-Cobo C, et al., 2024^40^	Comparar la eficacia de una intervención de salud móvil (*mHealth* ^*‡‡*^ ) utilizando la aplicación eMOTIVA con la de los cuidados habituales, en relación a seguir las pautas de rehabilitación cardíaca y considerando aspectos de estilo de vida, factores de riesgo cardiovascular y satisfacción entre pacientes con síndrome coronario agudo.	España	Ensayo clínico aleatorizado y controlado, con diseño de grupos paralelos.	300 pacientes con EAC* ya sometidos a una ICP^∥∥^ con implante de *stent* después de un episodio de síndrome coronario agudo (150 en el GI^‡^ y 150 en el Grupo Control). *Media de edad:* 62,5±8,7 años *Sexo:* 69,0% masculino*Comorbilidad:* EAC*.	GC^†^ *:* cuidados usuales. GI^‡^: acceso a *mHealth* ^*‡‡*^ (aplicación e-MOTIVA). La aplicación incluye diversa información científica para orientar al usuario en sus esfuerzos por alcanzar las metas terapéuticas recomendadas en las pautas clínicas y a mantener un estilo de vida saludable.	Desenlaces *- Práctica de actividad física: International Physical Activity Questionnaire* (IPAQ) y Prueba de caminata de 6 minutos; *- Mantener una alimentación sana: Adherence to the Mediterranean Diet* y cuestionario de frecuencia de consumo en la última semana. *- Nivel de conocimiento sobre factores de riesgo* (estrés y ansiedad; PA^§^ elevada; glicemia; colesterol; obesidad): escala elaborada y validada por los investigadores, con 24 ítems. - *Dejar de fumar:* autoinforme y prueba de Fagerström *- Peso corporal y altura para calcular el Índice de Masa Corporal - IMC* ^**^: se midió en persona; *- Circunferencia de la cintura:* se midió en persona; *- PA* ^*§*^ sistólica y diastólica y frecuencia cardíaca: se midieron en persona; *- Valores de lípidos (colesterol total, HDL-C* ^*¶¶*^ , LDL-C ^*∥*^ y triglicéridos), HbA1c ^***^ y glicemia: recolección de muestra de sangre. Momentos de recolección: Basal y después de 3 y 6 meses de seguimiento.	El GI^‡^ presentó un desempeño significativamente mejor que el GC^†^ para todos los desenlaces con diferencias estadísticamente significativas. *- Práctica de actividad física:* actividad física (minutos/semana) después de 3 meses de seguimiento (578,10+326,14 versus 443,46+278,11; p<0,001) y después de seis meses (614,51+332,26 versus 408,40+274,49; p<0,001); actividad física (METs/semana) después de 3 meses de seguimiento (1991,74+1176,71 versus 1490,48+925,89; p<0,001) y después de seis meses (2112,66+1196,67 versus 1372,60+944,62; p<0,001); horas sentado/semana después de 3 meses de seguimiento (8,57+1,89 versus 9,34+2,13; p=0,002) y después de seis meses (8,38+1,88 versus 9,59+2,09; p<0,001); Prueba de caminata de seis minutos después de seis meses (473,49+102,28 versus 447,25+93,68; p=0,04). *- Mantener una alimentación sana:* adhesión a la Dieta Mediterránea después de 3 meses de seguimiento (11,63+1,70 versus 9,32+2,55; p<0,001) y después de seis meses (11,92+1,70 versus 8,92+2,66; p<0,001); buena adhesión a la dieta recomendada después de 3 meses de seguimiento (93,8% versus 67,6%; p<0,001) y después de seis meses (94,4% versus 60,3%; p<0,001). *- Nivel de conocimiento sobre factores de riesgo:* después de 3 meses de seguimiento (116,14+4,23 versus 111,02+6,94; p<0,001) y después de seis meses (117,85+3,83 versus 111,00+7,11; p<0,001). - *Dejar de fumar:* Después de 3 meses de seguimiento (62,3% versus 53,2%; p=0,35) y después de seis meses (65,3% versus 46,8%; p=0,06); adicción a la nicotina después de 3 meses de seguimiento (2,30+2,27 versus 4,14+2,96; p=0,03) y después de seis meses (2,18+2,37 versus 3,64+3,02; p=0,10). *- IMC* ^**^: después de 3 meses de seguimiento (28,33+4,48 versus 28,24+4,40; p=0,86) y después de seis meses (28,22+4,69 versus 28,33+4,74; p=0,69).
Cruz-Cobo C, et al., 2024^40^	Comparar la eficacia de una intervención de salud móvil (*mHealth* ^*‡‡*^ ) utilizando la aplicación eMOTIVA con la de los cuidados habituales, en relación a seguir las pautas de rehabilitación cardíaca y considerando aspectos de estilo de vida, factores de riesgo cardiovascular y satisfacción entre pacientes con síndrome coronario agudo.	España	Ensayo clínico aleatorizado y controlado, con diseño de grupos paralelos.	300 pacientes con EAC* ya sometidos a una ICP^∥∥^ con implante de *stent* después de un episodio de síndrome coronario agudo (150 en el GI^‡^ y 150 en el Grupo Control). *Media de edad:* 62,5±8,7 años *Sexo:* 69,0% masculino*Comorbilidad:* EAC*.	GC^†^ *:* cuidados usuales. GI^‡^: acceso a *mHealth* ^*‡‡*^ (aplicación e-MOTIVA). La aplicación incluye diversa información científica para orientar al usuario en sus esfuerzos por alcanzar las metas terapéuticas recomendadas en las pautas clínicas y a mantener un estilo de vida saludable.	Desenlaces *- Práctica de actividad física: International Physical Activity Questionnaire* (IPAQ) y Prueba de caminata de 6 minutos; *- Mantener una alimentación sana: Adherence to the Mediterranean Diet* y cuestionario de frecuencia de consumo en la última semana. *- Nivel de conocimiento sobre factores de riesgo* (estrés y ansiedad; PA^§^ elevada; glicemia; colesterol; obesidad): escala elaborada y validada por los investigadores, con 24 ítems. - *Dejar de fumar:* autoinforme y prueba de Fagerström *- Peso corporal y altura para calcular el Índice de Masa Corporal - IMC* ^**^: se midió en persona; *- Circunferencia de la cintura:* se midió en persona; *- PA* ^*§*^ sistólica y diastólica y frecuencia cardíaca: se midieron en persona; *- Valores de lípidos (colesterol total, HDL-C* ^*¶¶*^ , LDL-C ^*∥*^ y triglicéridos), HbA1c ^***^ y glicemia: recolección de muestra de sangre. Momentos de recolección: Basal y después de 3 y 6 meses de seguimiento.	*- Circunferencia de la cintura:* después de 3 meses de seguimiento (102,56+11,53 versus 102,22+10.35; p=0,80) y después de seis meses (101,59+11,87 versus 102,09+10,80; p=0,72). *- PA* ^*§*^ sistólica: después de 3 meses de seguimiento (128,96+15,87 versus 133,27+14,85; p<0,001) y después de seis meses (130,00+21,90 versus 135,78+16,73; p<0,001). *- PA* ^*§*^ diastólica: después de 3 meses de seguimiento (73,68±9,76 versus 76,05±11,42; p=0,06) y después de seis meses (117,85+3,83 versus 111,00+7,11; p<0,001). *- FC* ^*§§*^: después de 3 meses de seguimiento (66,75±8,91 versus 71,93±9,86; p<0,001) y después de seis meses (68,20±10,13 versus 70,43±10,80; p=0,07). *- Perfil de lípidos (colesterol total, HDL-C* ^*¶¶*^ , LDL-C ^*∥*^ y triglicéridos): presentaron reducciones manifiestas en ambos grupos, aunque sin diferencias significativas entre ellos en ningún momento*:* después de 3 meses de seguimiento. *- HbA1c* ^***^: después de meses de seguimiento (6,61±1,40% versus 6,19±0,84%; p=0,25) y después de seis meses (6,44±1,01% versus 6,69±1,02%; p=0,36). *- Glicemia:* después 3 meses de seguimiento (110,58±35,92 mg/dL versus 112,44±34,38 mg/dL; p=0,48) y después de seis meses (116,48±45,55 mg/dL versus 128,06±55,69 mg/dL; p=0,02).
Ghavami M, et al., 2024^17^	Evaluar si la aplicación móvil “*Green Heart*” (con módulos de manejo de PA^§^, DLP^†††^ y para dejar de fumar) mejora el control de la PA^§^ y de los lípidos en pacientes con EAC*.	Irán	Ensayo clínico aleatorizado, paralelo, cegado para los evaluadores de resultados (*single-blinded*) y con dos ramas.	Total de sujetos elegibles: 1590 pacientes con EAC*, incluyendo 1114 hipertensos y 1488 personas con dislipidemia. - En el análisis de la hipertensión participaron 545 individuos en el GC^†^ y 546 en el GI^‡^; para la dislipidemia se incluyeron 728 en el Grupo Control y 714 en el Grupo Experimental. *Media de edad:* no informada *Sexo:* no informado*Comorbilidad:* EAC*.	GC^†^: instrucción tradicional sobre los mismos temas en formato impreso. GE: se usó una aplicación con tres módulos principales (control de la PA^§^ y de la DLP^†††^ y para dejar de fumar), además de incluir un lugar para la automonitorización, mensaje motivacionales, recordatorios, *feedback* automático e instrucción.	Desenlaces *- Control de la PA* ^*§*^: buenos resultados en materia de control de la PA^§^ en hipertensos; *- Control de la DLP* ^*†††*^: proporción de pacientes con dislipidemia y que presentaron valores de LDLC^∥^<70 mg/dL. Momentos de recolección: *Baseline* y 6 meses después de analizar los desenlaces.	*- Control de la PA* ^*§*^: el 88,6% de los participantes del GI^‡^ lograron controlar su PA^§^ versus 78,5% en el GC^†^ (OR=2,13, IC95%: 1,51; 3,03; p<0,001). *- Control de la DLP* ^*†††*^: el 24,8% de los participantes del GI^‡^ lograron controlar su PA^§^ versus 16,1% no GC^†^ (OR=1,72, IC95%: 1,32; 2,26; p<0,001).
Ghavami M, et al., 2024^41^	Evaluar el efecto ejercido por el módulo para dejar de fumar de la aplicación móvil “*Green Heart*” sobre cambios conductuales en personas con EAC* que fuman.	Irán	Ensayo clínico aleatorizado y en paralelo, con cegamiento de evaluadores (*single-blinded*) y dos ramas.	1590 individuos con EAC* elegibles, de los cuales 668 eran fumadores. Se analizaron 332 del GC^†^ y 102 del Grupo Control. *Media de edad:* no informada *Sexo:* no informado*Comorbilidad:* EAC*.	GC^†^: instrucción tradicional sobre los mismos temas (tabaquismo, riesgo cardiovascular), en formato impreso y sin utilizar la aplicación. GE: se usó la aplicación “*Green Heart*”, que dispone de un módulo específico para dejar de fumar, incluida una evaluación automatizada de la adicción a la nicotina, elaboración de un plan personalizado para dejar de consumir tabaco, contenidos educativos, mensajes motivacionales y *feedback* en tiempo real para brindar apoyo a la adhesión del paciente.	Desenlaces *- Dejar de fumar:* abstinencia continua por al menos 4 semanas durante el seguimiento; reducción en el consumo de tabaco (cantidad de cigarrillos) o cambio en la condición de fumador. Momentos de recolección: Basal y 24 semanas después de iniciadas las intervenciones.	En el análisis por intención de tratar, el GI^‡^ alcanzó índices significativamente más altos en cuanto a dejar de fumar y reducciones en el consumo de tabaco, en comparación con el Grupo Control (OR=2,14, IC95%: 1,16; 3,97; p=0,015).
Waranski M, et al., 2024^42^	Evaluar la eficacia del programa personalizado “*RehaPlus+*” de *eHealth* ^*‖*^ para promover la actividad física periódica en pacientes con EAC* después de la fase II de rehabilitación.	Alemania	Ensayo controlado con grupos paralelos.	169 pacientes con EAC* después de implante de *stent* o revascularización, luego de la rehabilitación de fase II (44 en el grupo *RehaPlus+*; 61 en el grupo *usual care*). *Media de edad:* no informada *Sexo:* no informado*Comorbilidad:* EAC*.	GC^†^: cuidados convencionales ambulatorios (24 sesiones de entrenamiento de fuerza y resistencia de 90 minutos durante 6 meses). GI^‡^: programa “*RehaPlus+*” de *eHealth* por 24 semanas, mensajes motivacionales dos veces por semana+ apoyo de un *case manager*.	Desenlaces *- Actividad física (minutos/semana) y AVD* ^*‡‡‡*^: cuestionario *Bewegungs- und Sportaktivität*) Momentos de recolección: Basal y seis meses después del seguimiento.	- Actividad física (minutos/semana): después de 6 meses, el grupo *RehaPlus+* registró una media de 182±208 minutos/semana de actividad física versus 119±175 minutos/semana en el Grupo Control (p=0,15). - Nivel de AVD^‡‡‡^: 442,79±469,1 minutos/semana en el grupo *RehaPlus+* versus 308,27± 411,52 en el Grupo Control (p= 0,20).
Xu L, et al., 2024^43^	Investigar si una intervención de gamificación basada en *smartphones* podría mejorar el índice de participación en actividades físicas y otros desenlaces físicos y psicológicos en pacientes con EAC*.	China	Ensayo clínico aleatorizado, controlado y con tres ramas.	108 pacientes con EAC*, asignados a tres grupos: 34 participantes en el GC^†^, 36 en el GI^‡^ individual gamificado y 38 en el GI^‡^ en equipo (gamificación asociada a interacción social). *Media de edad:* no informada *Sexo:* no informado*Comorbilidad:* EAC*.	GC^†^: intervención estándar (definición de metas de pasos en la aplicación, sin gamificación adicional) por 12 semanas. GI individual: se usó la aplicación con gamificación de forma individual (metas de pasos, recompensas, *feedback*) por 12 semanas. GI^‡^ en equipo: se usó la misma gamificación que en el grupo individual, pero asociada al componente social de un equipo, por 12 semanas.	Desenlaces - *Nivel de actividad física:* cambio en la cantidad de pasos diarios; proporción de días en los que se alcanzó la meta de pasos. *- IMC* ^**^ y circunferencia de la cintura: se midieron en persona. Momentos de recolección: Basal y después de 12 semanas de las intervenciones.	En el GI^‡^ individual, la intervención gamificada aumentó significativamente la cantidad de pasos diarios en comparación con el GC^†^ (+988 pasos; IC95%: 259; 1717; p<0,01), efecto que se mantuvo en el seguimiento (+819 pasos; IC95%: 24; 1613; p<0,01). Además, se observaron diferencias estadísticamente significativas en el IMC^**^ y la circunferencia de la cintura entre el GC^†^ y el grupo individual. En el GI^‡^ en equipo (donde la gamificación incluía interacción social), no se registró un aumento significativo en el nivel de actividad física en relación con el GC^†^.

*EAC = Enfermedad de las Arterias Coronarias; ^†^GC = Grupo Control; ^‡^GI = Grupo Intervención; ^§^PA = Presión Arterial; ^∥^LDL-C = *Low-Density Lipoprotein Cholesterol*; ^¶^VO_2_ = Consumo máximo de oxígeno; ^**^IMC = Índice de Masa Corporal; ^††^DTx = Terapia Digital; ^‡‡^ *mHealth* = *Mobile Health*; ^§§^FC = Frecuencia Cardíaca; ^∥∥^ICP = Intervención Coronaria Percutánea; ^¶¶^HDL-C = *High-Density Lipoprotein Cholesterol*; ^***^HbA1c = Hemoglobina Glicada; ^†††^DLP = Dislipidemia; ^‡‡‡^AVD = Actividades de la Vida Diaria

La síntesis de los 14 ensayos clínicos aleatorizados que se incluyeron en esta revisión sistemática evidenció amplia heterogeneidad en cuanto a las intervenciones digitales, los desenlaces evaluados y los métodos de medición, aunque con una tendencia general de resultados positivos en componentes específicos de la prevención secundaria del SCC. Se observó eficacia estadística a favor de *mHealth* en el control de factores de riesgo clínico (54,5%), en la adhesión al tratamiento medicamentoso (60%) y, de forma más expresiva, en la promoción de actividad física (71,4%), en dejar de fumar (100%) y en la adhesión a dietas saludables (100%). En contrapartida, los desenlaces de salud mental presentaron resultados más modestos e inciertos (50%). Es importante destacar que, entre las recomendaciones previstas en las pautas brasileñas, no se trataron tres temas en ninguno de los estudios analizados: reducción en el consumo de alcohol, vacunación contra la gripe y pautas orientadoras relacionadas con la actividad sexual, lo que revela vacíos relevantes en el desarrollo de aplicaciones dirigidas a la población con SCC.

La síntesis correspondiente a la certeza de la evidencia para cada desenlace analizado (fundamentada en los dominios del sistema GRADE) se detalla en la Figura 4.

**Figura 4:** Síntesis correspondiente a la calidad de la evidencia por desenlaces, de acuerdo con la metodología GRADE. São Paulo, SP, Brasil, 2025

Desenlace	Riesgo de sesgo	Inconsistencia	Evidencia indirecta	Imprecisión	Sesgo de publicación	Certeza global
Control de factores de riesgo^17^ ^,^ ^31^ ^-^ ^36^ ^,^ ^38^ ^-^ ^40^ ^,^ ^43^	🟡 Grave*	🟢 No grave	🟢 No grave	🟢 No grave	🟢 No sospechado	🟡 Moderada
Adhesión a tratamientos medicamentosos^31^ ^,^ ^34^ ^-^ ^36^ ^,^ ^39^	🟡 Grave*	🟢 No grave	🟢 No grave	🟢 No grave	🟢 No sospechado	🟡 Moderada
Actividad física^32^ ^-^ ^33^ ^,^ ^38^ ^-^ ^40^ ^,^ ^42^ ^-^ ^43^	🟡 Grave*	🟡 Grave^†^	🟢 No grave	🟡 Grave^‡^	🟡 Sospechado^§^	🔴 Baja
Dejar defumar^34^ ^,^ ^39^ ^-^ ^41^	🟡 Grave*	🟢 No grave	🟡 Grave^||^	🟢 No grave	🟢 No sospechado	🔴 Baja
Alimentación saludable^39^ ^-^ ^40^	🟡 Grave*	🟢 No grave	🟡 Grave^||^	🟡 Grave^‡^	🟢 No sospechado	🔴 Baja
Ansiedad/Depresión/Estrés^37^ ^-^ ^38^	🔴 Muy grave^¶^	🟡 Grave^†^	🟡 Grave^||^	🟡 Grave^‡^	🟡 Sospechado^§^	🔴 Baja
Reducción en el consumo de alcohol	-	-	-	-	-	-
Vacunación contra la gripe	-	-	-	-	-	-
Pautas sobre actividad sexual	-	-	-	-	-	-

*Degradado por la naturaleza abierta (sin cegamiento) de la intervención *mHealth*; ^†^Degradado debido a la alta heterogeneidad estadística y clínica (distintos métodos de intervención y medición); ^‡^Degradado por haber incluido estudios piloto con muestras pequeñas (n<50) e intervalos de confianza amplios; ^§^Sospechado en desenlaces conductuales debido a la ausencia de registros de protocolos para estudios con resultados positivos en muestras pequeñas; ^||^Degradado por haber utilizado desenlaces basados en autoinforme (*self-report*) sin validación bioquímica ni observación directa; ^¶^Degradado en dos niveles debido a la combinación de ausencia de cegamiento en desenlaces subjetivos y altos índices de desgaste (>20%)

Evaluada conforme a la metodología GRADE, la certeza de la evidencia varió de moderada a baja entre los desenlaces evaluados, tal como se sintetiza en la Figura 4. El control de factores de riesgo y la adhesión a tratamientos medicamentosos presentaron certeza moderada, solo degradada en el dominio de riesgo de sesgo debido a la naturaleza abierta (sin cegamiento) intrínseca de las intervenciones de *mHealth*. En contrapartida, los desenlaces de actividad física, dejar de fumar, alimentación saludable y salud mental (ansiedad, depresión y estrés) se clasificaron como de baja certeza.

A su vez, esta degradación se debió a la alta heterogeneidad (inconsistencia), al uso de medidas autoinformadas sin emplear datos cuantificables como utilizar un podómetro (evidencia indirecta), y a la imprecisión resultante de estudios piloto con muestras pequeñas. En el desenlace de salud mental, el riesgo de sesgo se consideró muy grave debido a la combinación de ausencia de cegamiento y elevados índices de desgaste (>20%). El análisis de cada uno de los artículos (por desenlace) se puede consultar en el material suplementario (disponible en https://doi.org/10.48331/SCIELODATA.CR4I8U).

### Segunda etapa - Análisis sobre la facilidad de uso de las aplicaciones seleccionadas

En esta etapa, el primer paso fue buscar aplicaciones en Google Play^®^ y en Apple Store^®^, siguiendo el método de búsqueda previamente estandarizado. Para cada categoría de prevención secundaria del SCC se seleccionó la aplicación mejor evaluada, considerando simultáneamente la cantidad de evaluaciones de los usuarios y el promedio de estrellas, a fin de reducir sesgos resultantes de clasificaciones sobrestimadas o con pocas evaluaciones.

Las aplicaciones identificadas trataron de manera fragmentada distintos dominios de la prevención secundaria del SCC, a saber: adhesión a tratamientos medicamentosos, práctica de actividad física, alimentación saludable, control de la presión arterial y de la glicemia, dejar de fumar, reducción en el consumo de bebidas alcohólicas, manejo de la ansiedad, control del peso corporal y vacunación contra la gripe. En general, las aplicaciones relacionadas con la actividad física, la adhesión a tratamientos medicamentosos y la alimentación saludable presentaron mayor cantidad de evaluaciones y mejores puntuaciones promedio en ambas plataformas.

En consecuencia, se destacaron aplicaciones ampliamente utilizadas como *MyTherapy - Lembrete de Remédios*, *Alarme de Medicamentos*, *Strava* y *MyFitnessPal*, reflejando mayor diseminación y aceptación entre los usuarios. En oposición, dominios como Dejar de fumar, Control del consumo de alcohol, Control de la presión arterial y Control de la glicemia presentaron menos aplicaciones y menor volumen de evaluaciones, especialmente en Apple Store^®^. En el dominio Vacunación contra la gripe se identificaron predominantemente aplicaciones institucionales como *Meu SUS Digital* y *e-saúde SP*.

Además, no se identificaron aplicaciones específicas dirigidas a la actividad sexual ni aplicaciones que trataran de forma integrada todos los dominios de la prevención secundaria del SCC, evidenciando que las soluciones digitales disponibles se concentran en comportamientos o factores de riesgo aislados.

Luego de la fase de selección, las aplicaciones disponibles en Google Play^®^ mejor evaluadas por los usuarios en cada categoría (*My Therapy*; *Treino em Casa & Exercícios*; *Alimente-se - Dieta e Nutrição*; *Rootd - Alívio da Ansiedade*; *Treino para Mulheres, Fitness*; *Parar de Fumar Assistente*; *Rastreador de Pressão Arterial*; *mySugar - Controle a Diabetes*; *Balcão de Sobriedade - EasyQuit*; *Meu SUS Digital*; *Dicas de Saúde e Alimentação*; y *WeightWar - Rastreador de Peso*) fueron analizadas en cuanto a su facilidad de uso por 24 pacientes internados con diagnóstico médico de SCC.

Se destaca que la mitad de los participantes de esta etapa pertenecía al sexo masculino y que la mayoría (83%) estaba internada por infartos agudos de miocardio. Además, más de la mitad (70%) eran ex fumadores y tenían Hipertensión Arterial Sistémica (HAS) (83%) y antecedentes familiares de enfermedades cardiovasculares (87%). Por otro lado, las personas alcohólicas (7%), fumadoras (23%) y portadoras de DM (30%) eran minoritarias.

Las aplicaciones se distribuyeron aleatoriamente entre los 24 participantes. Realizada por medio de la escala SUS, la evaluación referente a la facilidad de uso reveló amplia variación en las puntuaciones, tal como se indica en la Tabla 1.

**Tabla 1:** Puntuaciones mínima y máxima, valores medios, desviaciones estándar y clasificación correspondiente a la facilidad de uso de las aplicaciones. São Paulo, SP, Brasil, 2024

Aplicación	Puntuaciones mínima y máxima	Media	Desviación estándar	Clasificación SUS*
*My Therapy*	34 - 60	50,0	26,41	Promedio
*Treino em Casa & Exercícios*	87,5 - 55	69,58	14,78	Buena
*Alimente-se - Dieta e nutrição*	90 - 47,5	53,33	18,82	Buena
*Rootd - Alívio da Ansiedade*	90 - 15	54,58	25,02	Buena
*Treino para Mulheres, Fitness*	100 - 37,5	77,50	27,48	Excelente
*Parar de Fumar Assistente*	85 - 52,5	72,08	12,39	Buena
*Rastreador de Pressão arterial*	90 - 57,5	68,33	12,31	Buena
*mySugr - Controle a diabetes*	87,5 - 42,5	69,17	18,55	Buena
*Balcão de sobriedade - EasyQuit*	92,5 - 55	70,0	15,08	Buena
*Meu SUS digital*	100 - 82,5	89,58	6,60	De lo mejor que pueda imaginarse
*Dicas de Saúde e Alimentação*	100 - 82,5	90,0	10,12	De lo mejor que pueda imaginarse
*WeightWar - Rastreador de peso*	100 - 35	79,17	23,96	Excelente

*SUS = *System Usability Scale*

Los puntos fuertes que presentó la facilidad de uso de las aplicaciones estuvieron relacionados con el deseo de utilizar el sistema con frecuencia y con la confianza en hacerlo. En contrapartida, el déficit principal que se identificó estuvo relacionado con la complejidad del sistema.

Al realizar el análisis de correlación entre las puntuaciones de la escala SUS y las características de las aplicaciones (categoría de la aplicación, evaluación promedio asignada por los usuarios en las tiendas virtuales y cantidad de evaluaciones), se observó que no hubo ninguna correlación significativa entre la evaluación promedio de los usuarios y las puntuaciones obtenidas en la escala SUS (r=0,12; p=0,58), así como que la cantidad de evaluaciones tampoco presentó ninguna correlación significativa (r=0,08; p=0,69). Por otro lado, la categoría de las aplicaciones ejerció una influencia parcial sobre la facilidad de uso percibida: aplicaciones de actividad física y alimentación presentaron puntuaciones medias más altas (aproximadamente 70-80) en comparación con las de control de la glicemia o las relacionadas con la adhesión a tratamientos medicamentosos (aproximadamente 50-60). Estos hallazgos indican que la facilidad de uso percibida por los pacientes depende más de las funcionalidades y del diseño de cada aplicación que de su popularidad o evaluación general en las tiendas virtuales.

Además, se realizó un análisis de subgrupos para verificar si las características de los participantes ejercían alguna influencia sobre la facilidad de uso percibida. A tal fin, se compararon las medias de las puntuaciones SUS entre hombres y mujeres y entre participantes con y sin hipertensión arterial sistémica, diabetes *mellitus* y antecedentes familiares de enfermedades cardiovasculares, al igual que entre pacientes internados por infartos agudos de miocardio y aquellos con otras formas del síndrome coronario crónico.

Finalmente, en el análisis no se identificaron diferencias significativas entre los grupos, indicando que las características demográficas y clínicas de los participantes no influenciaron la facilidad de uso percibida. Estos hallazgos sugieren que las aplicaciones evaluadas presentan un desempeño consistente entre distintos perfiles de usuarios.

## Discusión

El presente estudio se condujo en dos etapas complementarias: una revisión sistemática de ensayos clínicos aleatorizados (ECA) y una evaluación con respecto a la facilidad de uso de aplicaciones disponibles en la tienda Google Play^®^, que incluyeran recomendaciones para la prevención secundaria del Síndrome Coronario Crónico (SCC). Es fundamental realizar un análisis integrado entre las evidencias de la literatura y las funcionalidades de las herramientas móviles para garantizar la efectividad y seguridad de las intervenciones de salud digital en esta población.

A pesar del significativo aumento en la cantidad de aplicaciones dirigidas a la salud cardiovascular, se observa que muchas de ellas carecen de una fundamentación científica sólida, ofreciendo funcionalidades cuya eficacia no posee comprobación clínica alguna. Eso quedó evidenciado en el presente estudio, donde se constató que, aunque diversas aplicaciones enfaticen la adopción de comportamientos saludables, pocos ensayos clínicos publicados en la literatura validan efectivamente el efecto ejercido por estas herramientas sobre los desenlaces clínicos.

Además, la revisión sistemática (compuesta por 14 ECA) demostró que, pese a la heterogeneidad en cuanto a funcionalidades, desenlaces evaluados y duración de las intervenciones, hay una tendencia global favorable a utilizar aplicaciones para modificar comportamientos de salud y controlar factores de riesgo cardiovasculares. También se observa que las intervenciones digitales son consistentemente eficaces en dominios de automanejo como la adhesión a tratamientos medicamentosos y la práctica de actividad física. Ciertos estudios demuestran que utilizar recordatorios personalizados y planes de autocuidado a través de aplicaciones no solo mejora las puntuaciones en escalas de adhesión sino que también se refleja en una mayor proporción de pacientes que alcanzan metas de presión arterial y niveles de lípidos en 12 meses^36^. Esta correlación sugiere que el apoyo a través de aplicaciones puede llenar un vacío crítico durante el período posterior al alta, ofreciendo un seguimiento continuo que el modelo de atención tradicional (muchas veces episódico) no logra sustentar.

Estos resultados son corroborados en diversos metaanálisis recientes, como el que evaluó la eficacia de diversas aplicaciones en reducir la cantidad de reingresos hospitalarios no planificados en pacientes que ya han sufrido un infarto agudo de miocardio o ya sometidos a una intervención coronaria percutánea. Los resultados demostraron que dichas intervenciones redujeron significativamente la cantidad de reinternaciones en comparación con los cuidados estándar (RR=0,45; IC95%: 0,23; 0,89; p=0,0219), con beneficios más manifiestos en estudios con seguimiento prolongado^44^. Otro metaanálisis (con 34 ensayos clínicos) evidenció que las aplicaciones móviles pueden reducir la incidencia de eventos cardíacos adversos de gran magnitud (RR=0,68; p=0,03), la tasa de reingresos hospitalarios (RR=0,56; p<0,001), el colesterol total (DMP=-0,19; p=0,03), los triglicéridos (DMP=-0,24; p<0,001) y la circunferencia abdominal (DMP=-1,92; p=0,01), además de promover mejoras en las puntuaciones de ansiedad y depresión. Entre los beneficios adicionales se incluyeron aumentos en la fracción de eyección del ventrículo izquierdo, del VO_2_ máximo, en la distancia recorrida en la Prueba de caminata de seis minutos y en los índices de adhesión a tratamientos medicamentosos^45^.

A pesar de estos efectos positivos, en la presente revisión se hizo evidente que la eficacia de las herramientas no es universal para todos los desenlaces. En lo referente a las variables conductuales, presentaron mejoras significativas; los indicadores antropométricos (como el índice de masa corporal y la circunferencia de la cintura) se mostraron más resistentes a la intervención aislada a través de la aplicación. De esta manera, en la práctica clínica tanto los enfermeros como los equipos multiprofesionales deben utilizar los dispositivos como elementos de apoyo que requieren supervisión humana, integrándolos a un asesoramiento nutricional y físico presencial. También se debe considerar la asociación de estrategias como la gamificación para ampliar los resultados. En un estudio que analizó una intervención de 12 semanas con elementos de gamificación (como metas de pasos, recompensas y *feedbacks*) se demostró que este enfoque fue significativamente superior al aumento en la cantidad de pasos diarios en comparación con el grupo en el que solo se utilizó la aplicación convencional. Por lo tanto, esto sugiere que la interactividad y el refuerzo positivo son esenciales para sustentar el compromiso activo de los pacientes^43^.

Se destaca que el SCC es una patología compleja en la que los pacientes se enfrentan a múltiples prescripciones y a la necesidad de implementar cambios sustanciales en su estilo de vida. Esta complejidad contribuye a la escasa adhesión a los tratamientos; eso redunda en que sea esencial que las herramientas digitales se creen sobre la base de teorías de cambio conductual. Sin embargo, pocos estudios informan dicha fundamentación teórica; entre los que sí lo hacen se destacan teorías cognitivo-sociales, de definición de metas, de autoeficacia y de autodeterminación^46^. La literatura indica que las intervenciones más eficaces son las que se diseñan de manera interactiva, están adaptadas culturalmente y son capaces de proporcionar sólidos contenidos educativos y motivacionales^47^ ^-^ ^50^.

Otro aspecto relevante que se identificó tanto en la revisión sistemática como en el análisis de las aplicaciones disponibles es que ninguna de ellas trata de forma integral todos los dominios de la prevención secundaria del SCC. Las herramientas centradas solamente en uno o dos dominios pueden fragmentar la atención, sin contemplar comportamientos críticos. La mayoría de las aplicaciones y de los estudios revisados se concentra en factores cuantificables como presión arterial y actividad física, puesto que son conductas fáciles de monitorizar y medir. Esta eficiencia en dominios mensurables es corroborada por diversas evidencias que indican mejoras en la capacidad de ejercicio físico y en la adhesión a tratamientos medicamentosos^40^ ^,^ ^51^. Por otro lado, dominios como Alimentación saludable, Manejo del estrés y Dejar de fumar se exploraron con menor frecuencia y presentaron resultados inconsistentes, lo que puede estar relacionado con la complejidad de estos comportamientos, que suelen exigir soporte clínico presencial o terapias conductuales individualizadas^51^ ^-^ ^52^. La ausencia de estos módulos indica que muchas aplicaciones siguen sin incorporar integralmente las recomendaciones estipuladas en las pautas más recientes, lo que puede poner en riesgo el automanejo.

Además, con respecto a la facilidad de uso que se analizó en la segunda etapa del estudio, se observó que la mayoría de las aplicaciones presentó un desempeño de bueno a excelente, con diseños amigables y navegación intuitiva. Los problemas técnicos y la complejidad de los sistemas se identificaron como factores de abandono, mientras que el *feedback* automático se asoció a mejores resultados^31^ ^-^ ^32^. A su vez, el análisis de correlación indicó que la evaluación promedio de los usuarios en las tiendas virtuales no se correlacionó significativamente con las puntuaciones obtenidas en la escala SUS; eso sugiere que la facilidad de uso técnica percibida depende más de las funcionalidades y del diseño que de la popularidad de cada aplicación. Entre las aplicaciones evaluadas, *Connect SUS* y *Dicas de Saúde e Alimentação* obtuvieron las puntuaciones más altas (destacándose por su simplicidad), mientras que *My Therapy* presentó el desempeño relativo más bajo. También se identificó un vacío en cuanto a explorar la actividad sexual, lo que refleja incomodidad y falta de preparación en los profesionales de salud al momento de tratar ese tema^39^ ^,^ ^41^.

En consecuencia, los hallazgos de esta investigación se condicen con la literatura sobre *mHealth*, evidenciando el potencial de aplicaciones para mejorar el automanejo y el compromiso activo^30^ ^-^ ^32^ ^,^ ^35^ ^,^ ^53^ ^-^ ^55^. Además, los resultados pueden interpretarse desde la perspectiva de teorías como el Modelo Transteórico o el Modelo de Aceptación Tecnológica (*Technology Acceptance Model*, TAM), que sugieren que la facilidad de uso y la utilidad percibida ejercen una influencia directa sobre la adhesión^44^ ^,^ ^56^. Desde el punto de vista práctico, para poder recomendar una aplicación en particular es necesario realizar una rigurosa selección previa, puesto que las interfaces complejas pueden desmotivar a pacientes de edad avanzada o con niveles de alfabetización digital más bajos. Además, la escasa integración de estos sistemas con historias clínicas electrónicas también constituye una barrera que obstaculiza la continuidad de la atención, puesto que sin comunicación bidireccional entre el registro de cada paciente y lo que pueden ver los equipos sanitarios, las aplicaciones quedan relegadas a ser un recurso aislado, perdiendo la oportunidad de servir como un sistema de alerta temprana para complicaciones cardiovasculares.

Finalmente, al considerar las limitaciones de la etapa de revisión sistemática de este estudio, es preciso reconocer la acentuada heterogeneidad de las intervenciones y el sesgo de selección inherente al uso de la tecnología. La mayoría de los estudios incluía poblaciones con acceso previo a *smartphones*; eso limita la posibilidad de generalizar los hallazgos a pacientes con niveles de alfabetización digital más bajos o con mayor debilidad clínica. Además, el tiempo de seguimiento (que mayormente no superó los seis meses) impide llegar a una conclusión definitiva sobre cómo mantener estos nuevos hábitos a largo plazo. Adicionalmente, la variación en las funcionalidades de las aplicaciones (que oscilan entre simples recordatorios pro SMS y complejos sistemas de gamificación y *coaching*) dificulta identificar cuál es el componente tecnológico esencial para lograr buenos resultados en materia de prevención secundaria.

Las limitaciones de la segunda etapa del estudio incluyen haber utilizado una muestra por conveniencia (lo que conlleva un sesgo de muestreo inherente a esta estrategia), además de la reducida cantidad de participantes. Es posible que haber restringido la muestra exclusivamente a pacientes internados haya subestimado barreras en cuanto a facilidad de uso y desafíos de compromiso activo enfrentados por pacientes ambulatorios en el contexto de la vida diaria en sus domicilios. Adicionalmente, la ausencia de pruebas con dispositivos del sistema iOS (Apple Store^®^) y la selección restricta a las aplicaciones mejor evaluadas n las tiendas virtuales impiden representar la totalidad de opciones disponibles en el mercado. Además, el breve período de uso quizás no refleje la utilización a largo plazo. También es posible que factores externos como ya conocer las tecnologías hayan interferido en las respuestas.

Finalmente, en futuros trabajos de investigación se deberán incluir ensayos clínicos con evaluaciones de costo-efectividad y estudios de implementación práctica, con la intención de identificar herramientas más seguras y sustentables para el automanejo cardiovascular.

## Conclusión

A modo de síntesis, las evidencias demuestran que las tecnologías móviles constituyen una estrategia coadyuvante de elevado potencial en la prevención secundaria del SCC, presentando resultados favorables en materia de promover la adhesión a tratamientos medicamentosos, controlar factores de riesgo y modificar estilos de vida. Emplear estas herramientas fortalece el automanejo y la monitorización continua, llenando el vacío asistencial entre consultas presenciales. No obstante, la acentuada heterogeneidad de las funcionalidades y el predominio de seguimientos a corto plazo indican que las tecnologías no deben considerarse como una solución aislada para mitigar desenlaces clínicos complejos. En el caso de la práctica clínica, se concluyó que la correcta implementación de *mHealth* depende de su integración con modelos híbridos de atención en los que los dispositivos digitales actúen como elemento de apoyo a la vigilancia y educación en salud. Esta integración permite que los equipos multiprofesionales apliquen intervenciones presenciales más intensivas a los pacientes que no alcanzan las metas terapéuticas señaladas por el sistema, optimizando así la seguridad y personalización de la atención.

## Data Availability

El conjunto de datos de este artículo está disponible en la página de la RLAE en el repositorio SciELO Data, en el enlace https://doi.org/10.48331/SCIELODATA.CR4I8U
